# Local c-di-GMP signaling, triggered by cross-regulation of cAMP-CRP and c-di-GMP, controls biofilm formation under nutrient limitation

**DOI:** 10.1073/pnas.2516964122

**Published:** 2025-08-25

**Authors:** Di Sun, Xiaobo Liu, Ying Zhang, Rui Shi, Yunrui Ru, Xuge Zhou, Ying Chen, Jing Yang, Jiawen Liu, Jingrong Zhu, Cong Liu, Weijie Liu

**Affiliations:** ^a^Department of Microbiology, School of Life Sciences, Jiangsu Normal University, Xuzhou 221116, China; ^b^Department of Biological Engineering, School of Environmental and Biological Engineering, Nanjing University of Science and Technology, Nanjing 210094, China; ^c^Department of Space Biology Research, School of Life Science, Beijing Institute of Technology, Beijing 100081, China

**Keywords:** nutrient limitation, biofilm, cross-regulation, cAMP-CRP, local c-di-GMP signaling

## Abstract

Bacteria have several nucleotide second messengers, most of which act as global regulators to control a wide range of bacterial physiological processes. Studies usually focus on a single second messenger, and the mechanisms and physiological significance of the cross-regulation between different nucleotide second messengers are often unclear. Here, we show that *Shewanella putrefaciens* can form biofilms in both nutrient-rich and nutrient-poor media. While both are controlled by c-di-GMP, the regulatory models differ. Under low nutrient conditions, cross-regulation of cAMP-CRP and c-di-GMP occurs at the transcriptional and posttranslational levels, thereby controlling biofilm development. During the early stages of biofilm development, cAMP-CRP directly promotes the transcription of a PDE gene, *lrbR*, by LrbA. Additionally, cAMP-CRP recruits LrbR to BpfD to suppress early biofilm formation via LrbR-dependent local degradation of c-di-GMP. Finally, as intracellular LrbR levels decrease, cAMP-CRP-BpfD enables a rapid shift to biofilm development and supports biofilm maintenance. Under high nutrient conditions, this cross-regulation does not occur, resulting in a positive correlation between global c-di-GMP levels and biofilm biomass. The identification of distinct modes of biofilm regulation in different nutrients will provide a theoretical basis for future targeted control of biofilm formation in different nutrient environments.

## Significance

Decades of research have established cAMP-CRP as a global transcription factor in bacteria. Here, we demonstrate that cross-regulation between cAMP-CRP and c-di-GMP occurs at both the transcriptional and posttranslational levels. Not only does cAMP-CRP regulate the transcription of *lrbR*, which encodes a phosphodiesterase (PDE), but it also interacts directly with LrbR and the c-di-GMP effector BpfD. This cross-regulation means that a moderate increase in intracellular c-di-GMP levels leads to a rapid increase in biofilm biomass, but only in a nutrient-limited environment. Since the proteins involved in this cross-regulation are conserved in many bacteria, this cross-regulation may be common to a wide range of bacteria in response to nutrient conditions.

As the nutrient status in the ecological niches of bacteria is often highly fluctuating, bacteria must adapt to different nutrient environments (1–3). Enterobacteria not only live in the nutrient-rich gut of mammals but also survive in nutrient-poor aquatic environments (2, 4). Marine bacteria can live in either the eutrophic coastal zone or the oligotrophic open sea (1, 5). Thus, the ability to adapt to nutrient transitions is critical for microbial fitness (6). Bacteria have several nucleotide second messengers, which are important molecules that regulate bacterial physiological processes to adapt to changing natural environments (7, 8). cAMP-CRP was first found in *Escherichia coli* to be involved in carbon catabolite repression (CCR), which is regulated by the phosphoenolpyruvate: carbohydrate phosphotransferase system (PTS) (9). However, this process is usually found in most Enterobacteriaceae, Vibrionales, and Firmicutes, but not in all bacteria, especially environmental bacteria, such as *Pseudomonas* and *Shewanella* (10–12). The optimal carbon sources of most environmental bacteria are organic acids or amino acids, rather than hexose sugars (10). Thus, in these bacteria, not only is cAMP-CRP not involved in CCR (10), but it is also unclear whether its regulation responds to the nutrient environment.

Biofilms are structural communities of sessile microbial cells embedded in a self-produced extracellular polymeric substance (EPS), which is the dominant lifestyle for bacterial survival in stressful environments (13–15). c-di-GMP is a critical second messenger that regulates bacterial biofilm development (16–18), which involves four stages: i) initial attachment, ii) microcolony formation, iii) biofilm maturation, and iv) dispersal (19, 20). The synthesis of the c-di-GMP is catalyzed by diguanylate cyclase (DGC) containing GGDEF domain, whereas the degradation of the c-di-GMP is catalyzed by phosphodiesterase (PDE) containing EAL or HD-GYP domain (16–18). The Lap system, which was first found in *Pseudomonas fluorescens,* plays an important role in biofilm development by responding to c-di-GMP signaling (21). This system has also been identified in some *Vibrio*, *Legionella*, *Bordetella*, and *Shewanella* bacteria (21). The Lap system in *Shewanella* is defined as BpfAGD system (22, 23). BpfA is an outer membrane adhesion protein that promotes cell attachment to solid surfaces, BpfG is a periplasmic protease, and BpfD is an inner membrane-spanning c-di-GMP effector containing degenerate GGDEF and EAL domains (23, 24). The BpfAGD system regulates biofilm development in response to intracellular c-di-GMP levels (23). In *Shewanella putrefaciens* CN32, cAMP-CRP is involved in the regulation of BpfAGD system at posttranslational level, which supports biofilm maintenance under limited intracellular c-di-GMP levels (24). However, the influence of this regulatory mechanism on early biofilm development is unclear.

cAMP and c-di-GMP are highly versatile signaling molecules, which both act as global regulators to control a wide range of bacterial physiological processes (7). Although most studies often focus on a single second messenger, cross-regulation of both has been identified in certain bacteria. Cross-regulation is a process that can be applied to any situation where the cAMP input alters the outcome of the c-di-GMP pathway, or vice versa. The present study showed that cross-regulation of cAMP-CRP and c-di-GMP occurs at transcriptional and posttranslational levels, thereby controlling biofilm development in nutrient-poor environments.

## Results

### cAMP-CRP Complex Negatively Regulates Biofilm Formation.

**Fig. 1.**
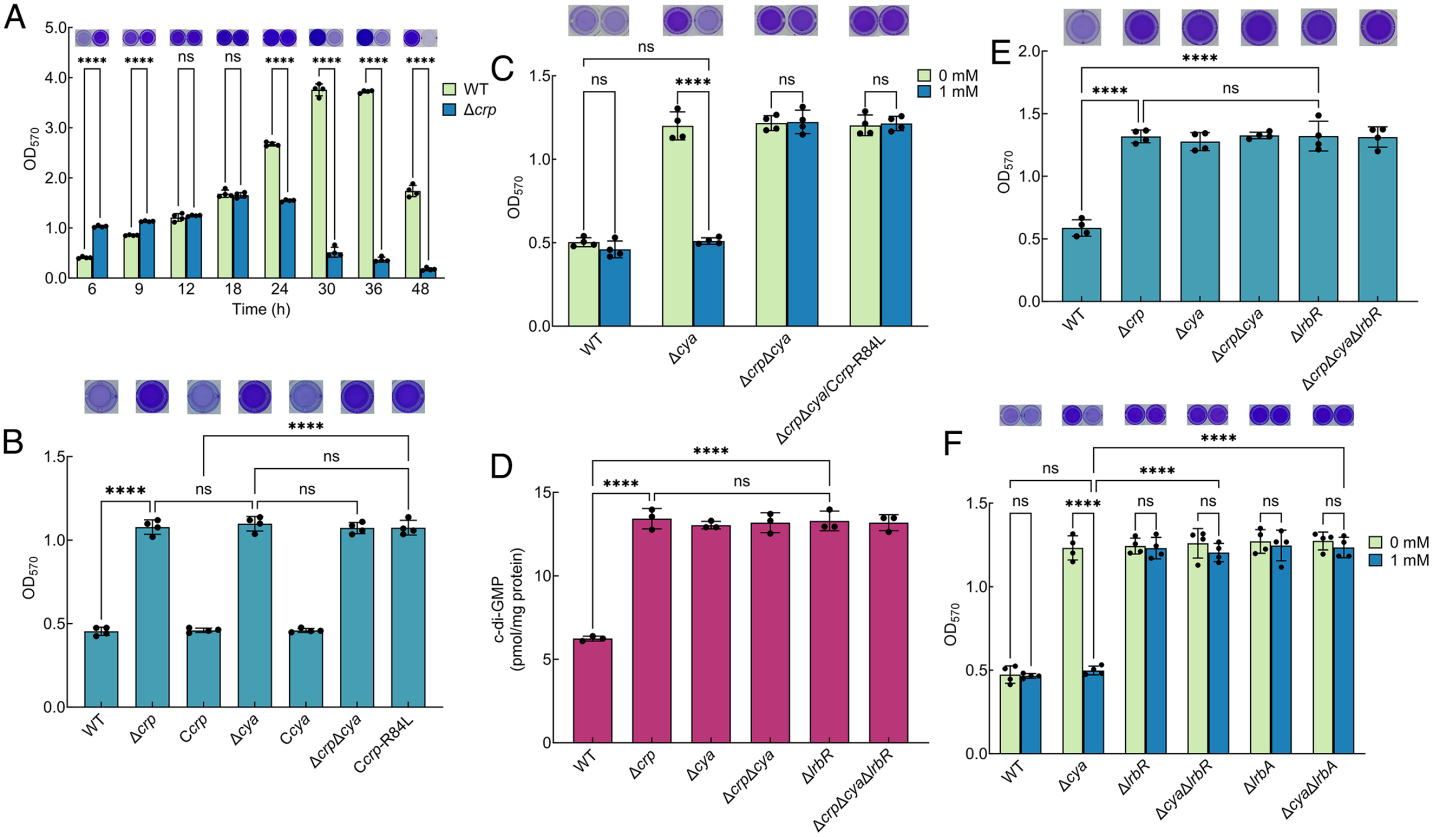
cAMP-CRP negatively regulates biofilm formation by controlling LrbR in *Shewanella putrefaciens* CN32. (*A*) Biofilm biomass (n = 4 independent samples). (*B*) Biofilm biomass at 6 h (n = 4 independent samples). (*C*) Biofilm biomass with the addition of 1 mM exogenous cAMP to the culture medium vs. control [no addition of exogenous cAMP (0 mM)] at 6 h (n = 4 independent samples). (*D*) Intracellular c-di-GMP concentration at 6 h (n = 3 independent samples). (*E*) Biofilm biomass at 6 h (n = 4 independent samples). (*F*) Biofilm biomass with the addition of 1 mM exogenous cAMP to the culture medium vs. control [no addition of exogenous cAMP (0 mM)] at 6 h (n = 4 independent samples). Insets in (*A*–*C*, *E*, and *F*) are the biofilm pictures of crystal violet dyeing. All strains used in (*A*–*F*) were cultured in MM1 medium. Data in (*A*–*F*) are shown as the mean ± SD. Two-way ANOVA (*A*, *C*, and *F*) and one-way ANOVA (*B*, *D*, and *E*) followed by Tukey’s multiple comparison tests were used to analyze the statistical significance, which was provided by GraphPad Prism 10 statistical software (ns: no significance, ****P < 0.0001).

A previous study found that WT and Δ*crp* had identical biofilms at 12 h, but the biofilm biomass of Δ*crp* was much lower than that of WT at 30 h, indicating that CRP plays an important role in biofilm maintenance (24) (Fig. 1*A*). In this study, we found that Δ*crp* had significantly higher biofilm biomass than WT at 6 h (Fig. 1*A*). The biofilm biomass of the complementation strain C*crp* was similar to that of WT at 6 h (Fig. 1*B*). The planktonic growth rate of Δ*crp* was slightly slower than that of WT and C*crp* before 12 h, but after 12 h it grew to a slightly higher cell density than that of the other two strains (*SI Appendix*, Fig. S1*A*). In addition, the ratio of biofilm biomass to cell growth (OD_570_/OD_600_) of Δ*crp* was still significantly higher than that of the WT and C*crp* at 6 h (*SI Appendix*, Fig. S1*B*), indicating that the increase in the biofilm biomass of Δ*crp* is not due to a change in cell growth. Thus, CRP negatively regulates the early biofilm development of *S. putrefaciens* CN32.

CRP is widely known as a cAMP-dependent transcription factor in most bacteria (25).The cAMP-CRP complex supports biofilm maintenance of *S. putrefaciens* CN32 (24).The biofilm biomass of cAMP-negative strain Δ*cya* was similar and significantly higher than that of WT at 6 h, whereas the biofilm biomass of C*cya* was restored to WT level at 6 h (Fig. 1*B*), indicating that cAMP, like CRP, negatively regulates early biofilm development. The addition of exogenous cAMP (1 mM) to the MM1 medium resulted in increased intracellular cAMP concentrations at 6 h in all strains studied (*SI Appendix*, Fig. S2*A*), but this increase only reduced the biofilm biomass of Δ*cya* at 6 h and had no effect on that of Δ*crp*Δ*cya* (Fig. 1*C*), indicating that the negative regulation of biofilm formation by cAMP requires the presence of CRP. In *S. putrefaciens* CN32, the site-directed mutant protein CRP-R84L loses the ability to bind cAMP (24). The biofilm biomass of C*crp*-R84L was significantly higher than that of WT and C*crp* and similar to that of Δ*crp* and Δ*cya* at 6 h (Fig. 1*B*). In addition, the intracellular cAMP concentrations of Δ*crp*Δ*cya*/C*crp*-R84L were significantly increased at 6 h after the addition of exogenous cAMP (*SI Appendix*, Fig. S2*A*), but this increase had no effect on the biofilm biomass (Fig. 1*C*). These results indicate that when CRP does not form a complex with cAMP, it loses the function of regulating biofilm formation. Thus, the early biofilm development of *S. putrefaciens* CN32 is negatively regulated by the cAMP-CRP complex.

### cAMP-CRP Negatively Regulates Biofilm Formation by Promoting *lrbR* Transcription.

A previous study has shown that cAMP-CRP supports biofilm maintenance (24) (Fig. 1*A*, 30 and 36 h). Then, we focused on investigating how cAMP-CRP negatively regulates early biofilm development (Fig. 1*A*, 6 h). The global intracellular c-di-GMP levels of Δ*crp*, Δ*cya*, and Δ*crp*Δ*cya* were similar, which were significantly higher than those of WT at 6 h (Fig. 1*D*), suggesting that cAMP-CRP regulates early biofilm development through the control of c-di-GMP signaling. *S. putrefaciens* CN32 has 47 GGDEF/EAL/HD-GYP domain proteins (24). As an in-frame deletion of Sputcn32_0498 could not be generated, the biofilm biomass of Δ*crp* and deletion mutants of 46 *dgc*/*pde* genes was compared at 6 h (*SI Appendix*, Fig. S3). The result showed that the biofilm biomass of Δ*crp* was similar to that of Δ*lrbR* at 6 h (*SI Appendix*, Fig. S3). LrbR has a typical EAL domain and negatively regulates early biofilm development by decreasing the intracellular c-di-GMP levels (26). The intracellular c-di-GMP levels and the biofilm biomass of WT, Δ*crp*, Δ*cya*, Δ*crp*Δ*cya*, Δ*lrbR*, and Δ*crp*Δ*cya*Δ*lrbR* were similar and significantly higher than those of WT at 6 h (Fig. 1 *D* and *E*), suggesting that cAMP-CRP and LrbR are involved in the same signaling pathway. Moreover, although the addition of exogenous cAMP increased intracellular cAMP concentrations of all strains (*SI Appendix*, Fig. S2*B*), it only restored the biofilm biomass of Δ*cya* to WT levels but had no effect on that of Δ*cya*Δ*lrbR* (Fig. 1*F*), indicating that LrbR is the downstream target of cAMP-CRP.

**Fig. 2.**
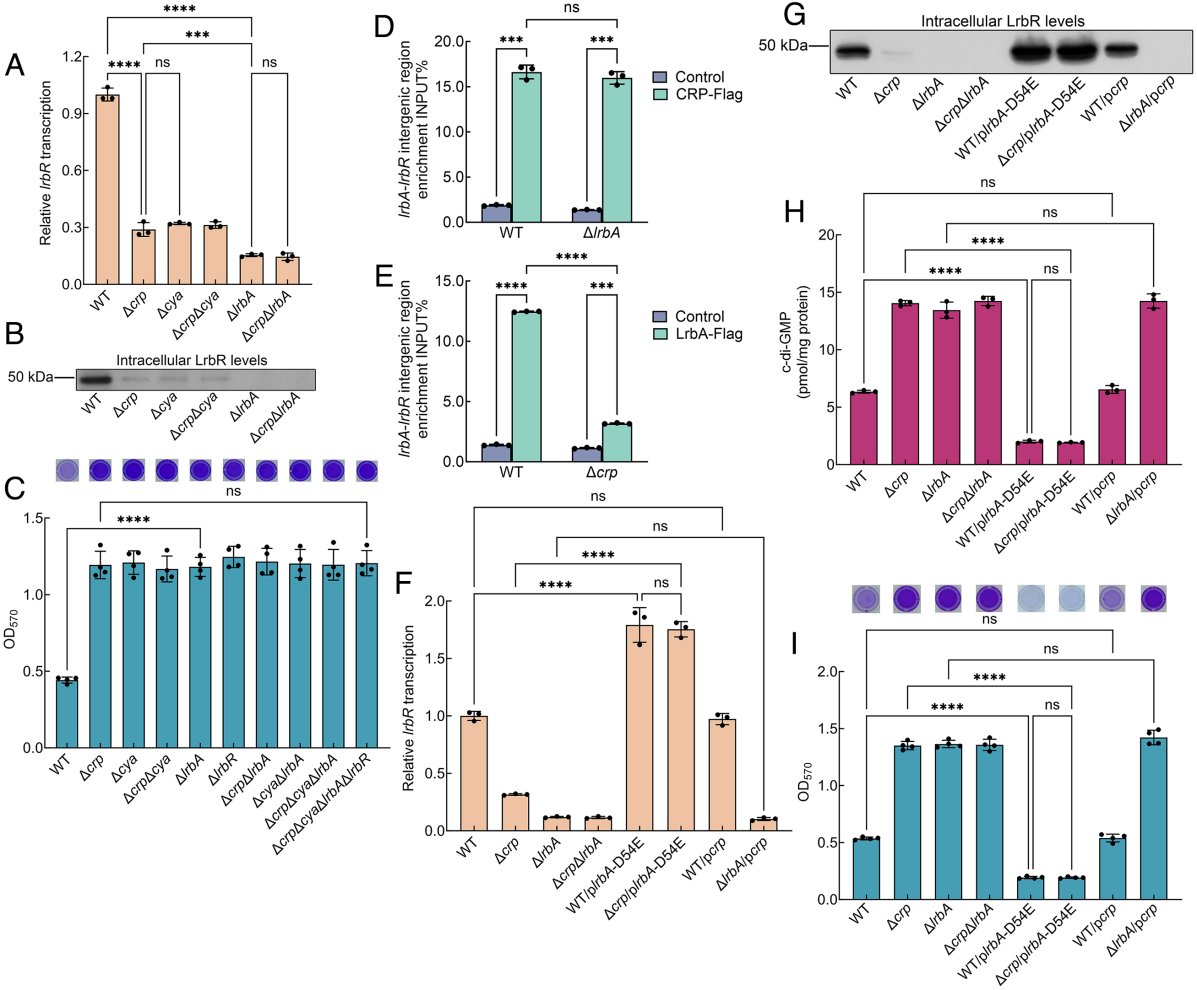
cAMP-CRP promotes the ability of LrbA to activate *lrbR* transcription. (*A*) Transcriptional analysis of *lrbR* at 6 h (n = 3 independent samples). The relative transcription value of the evaluated gene in the WT strain was set to 1. (*B*) Western blotting detection of intracellular LrbR levels at 6 h. (*C*) Biofilm biomass at 6 h (n = 4 independent samples). (*D*) In vivo ChIP-qPCR analysis of CRP-Flag binding to the intergenic region between *lrbR* and *lrbA* at 6 h. WT and Δ*lrbA* was used as a negative control, respectively (n = 3 independent samples). The Y-axis indicates the relative enrichment value of CRP at its target site, which was calculated by comparing the Ct value of the sample and the input. (*E*) In vivo ChIP-qPCR analysis of LrbA-Flag binding to the intergenic region between *lrbR* and *lrbA* at 6 h. WT and Δ*crp* was used as a negative control, respectively (n = 3 independent samples). The Y-axis indicates the relative enrichment value of LrbA at its target site, which was calculated by comparing the Ct value of the sample and the input. (*F*) Transcriptional analysis of *lrbR* at 6 h (n = 3 independent samples). The relative transcription value of the evaluated gene in the WT strain was set to 1. (*G*) Western blotting detection of intracellular LrbR levels at 6 h. (*H*) Intracellular c-di-GMP concentration at 6 h (n = 3 independent samples). (*I*) Biofilm biomass at 6 h (n = 4 independent samples). Insets in (*C* and *I*) are the biofilm pictures of crystal violet dyeing. All strains used in (*A*–*I*) were cultured in MM1 medium. Data in (*A*, *C*–*F*, *H*, and *I*) are shown as the mean ± SD. One-way ANOVA (*A*, *C*, *F*, *H*, and *I*) and two-way ANOVA (*D* and *E*) followed by Tukey’s multiple comparison tests was used to analyze the statistical significance, which was provided by GraphPad Prism 10 statistical software (ns: no significance, ***P < 0.001, ****P < 0.0001).

cAMP-CRP is a known global transcription factor. The electrophoretic mobility shift assay (EMSA) showed that in the presence of cAMP, CRP directly bound to the intergenic region between *lrbR* and its upstream gene (P_int_*_AR_*) (*SI Appendix*, Fig. S4). The qRT-PCR results showed that compared to WT, the transcription of *lrbR* in Δ*crp* was significantly downregulated at 6 h (Fig. 2*A*), which was at a similar level in Δ*crp*, Δ*cya*, and Δ*crp*Δ*cya* at 6 h (Fig. 2*A*). Western blotting showed that the intracellular LrbR protein levels of Δ*crp*, Δ*cya*, and Δ*crp*Δ*cya* were similar and significantly lower than those of WT (Fig. 2*B*), which were largely consistent with the tendency of *lrbR* transcription (Fig. 2*A*). Thus, cAMP-CRP negatively regulates intracellular c-di-GMP levels by promoting the transcription of *lrbR*, thereby negatively regulating the early biofilm development.

### cAMP-CRP Promotes the Ability of LrbA to Activate *lrbR* Transcription.

A previous study has shown that LrbR is a response regulator of a three-component regulatory system LrbS-LrbA-LrbR, which is involved in the same signal transduction pathway (26). LrbA, a transcription factor that regulates the transcription of *lrbR* by binding directly to the intergenic region between *lrbR* and its upstream gene, *lrbA* (26). We then investigated the regulatory relationship between cAMP-CRP and LrbA in the regulation of *lrbR* transcription. The biofilm biomass of Δ*crp* and Δ*cya* was similar to that of Δ*lrbA* and Δ*crp*Δ*cya*Δ*lrbA* (Fig. 2*C*), suggesting that cAMP-CRP and LrbA are involved in the same signaling pathway to regulate LrbR. The qRT-PCR result showed that while the *lrbR* transcription was significantly lower in both Δ*crp* and Δ*lrbA* than in WT (Fig. 2*A*), the *lrbR* transcription of Δ*crp*Δ*lrbA* was similar to that of Δ*lrbA* and was lower than that of Δ*crp* (Fig. 2*A*). Western blotting showed that the intracellular LrbR protein levels in these strains were largely consistent with the tendency of *lrbR* transcription (Fig. 2*B*). In addition, although the addition of exogenous cAMP increased intracellular cAMP concentrations of all strains (*SI Appendix*, Fig. S2*B*), it only restored the biofilm biomass of Δ*cya* to WT levels but had no effect on that of Δ*cya*Δ*lrbA* (Fig. 1*F*), suggesting that cAMP-CRP regulates the transcription of *lrbR* by controlling *lrbA*/LrbA.

cAMP-CRP directly binds to the intergenic region between *lrbA* and *lrbR* (P_int_*_AR_*) (*SI Appendix*, Fig. S4) that contains the bidirectional promoter region (26). We next investigated whether cAMP-CRP regulates the transcription of *lrbA*. The transcription levels of *lrbA* and the intracellular LrbA protein levels in Δ*crp* were similar to those in WT (*SI Appendix*, Fig. S5 *A* and *B*). *lrbS* is cotranscribed with *lrbA* (26), and the transcription levels of *lrbS* and the intracellular LrbS protein levels in Δ*crp* were similar to those in WT (*SI Appendix*, Fig. S5 *D* and *E*). These results indicate that cAMP-CRP does not regulate the transcription of the *lrbA*-*lrbS* operon. Thus, cAMP-CRP regulation of *lrbR* transcription is not through control of intracellular LrbA levels. The previous study found that phosphorylated LrbA has transcriptional regulatory activity, whereas dephosphorylated LrbA does not (26). Phos-tag PAGE analysis revealed that LrbA is phosphorylated in WT, Δ*crp*, Δ*cya,* and Δ*crp*Δ*cya* (the dephosphorylated LrbA mimic, LrbA-D54A, was used as a control) (*SI Appendix*, Fig. S5*C*), indicating that cAMP-CRP does not regulate the phosphorylation status of LrbA. Thus, cAMP-CRP regulation of *lrbR* transcription is not through control of LrbA phosphorylation status. We next sought to determine whether cAMP-CRP affects the ability of LrbA to bind the *lrbA*-*lrbR* intergenic region using EMSA and ChIP-qPCR assay. In the presence of LrbA, a 40-nt protected region was identified from −69 to −30 nt upstream of the transcription start site (TSS, +1) of *lrbR* (*SI Appendix*, Fig. S6*A*). As the sequence was mutated (*SI Appendix*, Fig. S6*B*), LrbA did not bind to the mutated probe (P_int_*_AR_*-mut) (*SI Appendix*, Fig. S6*C*), indicating that LrbA promotes *lrbR* transcription by binding to the 40-nt region that partially overlaps the −35 region of the *lrbR* promoter. Although we failed to find the CRP binding site, cAMP-CRP was still able to bind to mutated probes (P_int_*_AR_*-mut) of the LrbA binding sequence (*SI Appendix*, Fig. S6*D*). Then, the ChIP-qPCR assay was performed. A 3× Flag tagged at the C-terminal of CRP and LrbA did not affect the biofilm biomass of strains (*SI Appendix*, Fig. S7). The enrichment of CRP at the *lrbA-lrbR* intergenic region in Δ*lrbA* was similar to that in the WT (17-fold) (Fig. 2*D*), indicating that LrbA does not affect the ability of CRP to bind the *lrbA-lrbR* intergenic region. However, the enrichment of LrbA at the *lrbA-lrbR* intergenic region in Δ*crp* (threefold) was significantly downregulated compared to WT (12-fold) (Fig. 2*E*), indicating that CRP enhances the ability of LrbA to bind the *lrbR* promoter. GST pull-down and coimmunoprecipitation (Co-IP) assay results showed that CRP directly interacts with LrbA (*SI Appendix*, Fig. S8). Thus, CRP enhances the ability of LrbA to bind to the *lrbR* promoter by interacting with LrbA.

To further determine the regulatory relationship between CRP and LrbA in the regulation of *lrbR* transcription, we analyzed all phenotypes of strains overexpressing one protein (CRP or LrbA) but lacking the other (LrbA or CRP). For this analysis, *lrbA* and *crp* were overexpressed in the broad host plasmid by a constitutive promoter P*_aacC1_*, which increased the intracellular levels of both proteins (due to the extremely high levels of intracellular CRP protein in *S. putrefaciens* CN32, we labeled different tags for CRP in the chromosome and CRP in the plasmid) (*SI Appendix*, Fig. S9). In addition, only phosphorylated LrbA has transcriptional regulatory activity (26). Thus, we cannot be sure that the overexpressed LrbA is fully phosphorylated in the cell. Since the complementation of Δ*lrbA* with LrbA-D54E, a mimic of the phosphorylated LrbA protein, restored biofilm biomass to WT levels (26), LrbA-D54E was used for overexpression experiments. Δ*crp*/p*lrbA*-D54E and Δ*lrbA*/p*crp* strains fulfilled the requirements of overexpressing one protein but lacking the other one. The results showed that *lrbR* transcription and intracellular LrbR levels of WT/p*lrbA*-D54E were significantly higher than those of WT (Fig. 2 *F* and *G*), leading to decrease in intracellular c-di-GMP levels and biofilm biomass (Fig. 2 *H* and *I*). In addition, although the deletion of *crp* resulted in a significant decrease in *lrbR* transcription (Fig. 2*F*), the overexpression of *lrbA* reversed this condition (Fig. 2*F*). Thus, *lrbR* transcription and intracellular LrbR levels of Δ*crp*/p*lrbA*-D54E were significantly higher than those of WT and Δ*crp* (Fig. 2 *F* and *G*), resulting in decreased intracellular c-di-GMP levels and biofilm biomass (Fig. 2 *H* and *I*). These results indicate that *lrbR* transcription can be increased by overexpression of LrbA in the presence or absence of CRP (Fig. 2*F*). However, the overexpression of CRP in either WT or Δ*lrbA* did not affect all phenotypes (Fig. 2 *F*–*I*). Exogenous cAMP was added to the medium to culture WT/p*crp*, and Δ*lrbA*/p*crp* to rule out the possibility that intracellular cAMP levels are insufficient to form a complex with increased CRP. Although the addition of exogenous cAMP increased the intracellular cAMP levels of WT/p*crp* and Δ*lrbA*/p*crp* (*SI Appendix*, Fig. S10*A*), it did not affect the intracellular LrbR levels and biofilm biomass of either strain (*SI Appendix*, Fig. S10 *B* and *C*). These results indicate that LrbA is an essential transcription factor for the initiation of *lrbR* transcription and cAMP-CRP promotes *lrbR* transcription by enhancing LrbA binding to the *lrbR* promoter.

### LrbR Is Involved in a Local c-di-GMP Signaling Pathway Through Its Interaction with cAMP-CRP.

**Fig. 3.**
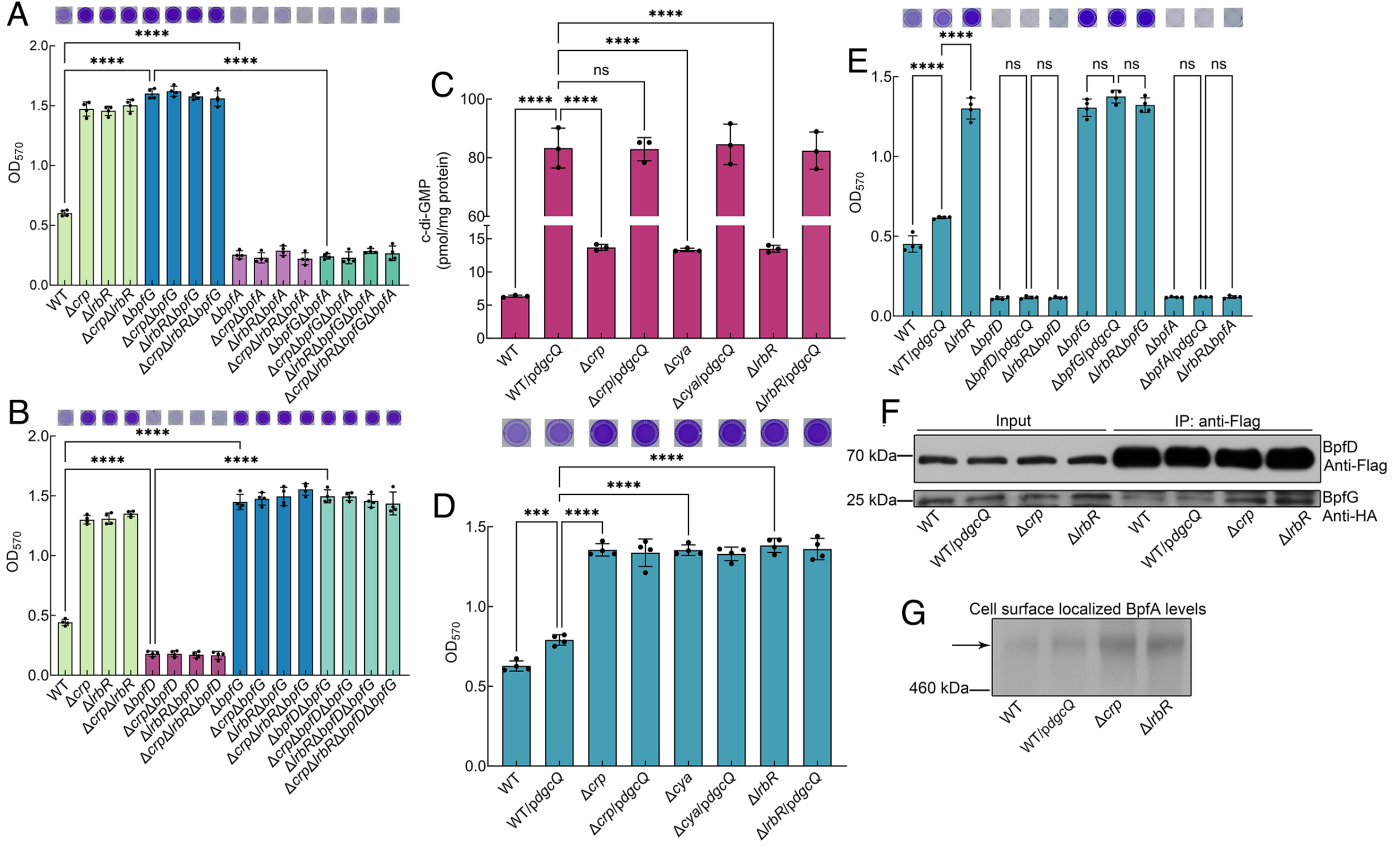
Regulation of global intracellular c-di-GMP levels is insufficient to recapitulate the effect of cAMP-CRP and LrbR on biofilm formation. (*A*) Biofilm biomass at 6 h (n = 4 independent samples). (*B*) Biofilm biomass at 6 h (n = 4 independent samples). (*C*) Intracellular c-di-GMP concentration at 6 h (n = 3 independent samples). (*D*) Biofilm biomass at 6 h (n = 4 independent samples). (*E*) Biofilm biomass at 6 h (n = 4 independent samples). (*F*) Co-IP to analyze the interaction between BpfD and BpfG in vivo at 6 h. 5 μM c-di-GMP was added to cell lysis of all samples. (*G*) Western blotting detection of cell surface localized BpfA levels at 6 h. The strains used in (*F* and *G*) contain the *bpfA* operon driven by the *aacC1* promoter to exclude the influence of *bpfA*, *bpfD*, *bpfG* transcription caused by the introduction of *dgcQ* or the deletion of *crp* and *lrbR*. Insets in (*A*, *B*, *D*, and *E*) are the biofilm pictures of crystal violet dyeing. All strains used in (*A*–*G*) were cultured in MM1 medium. Data in (*A*–*E*) are shown as the mean ± SD. One-way ANOVA followed by Tukey’s multiple comparison tests was used in (*A*−*E*) to analyze the statistical significance, which was provided by GraphPad Prism 10 statistical software (ns: no significance, ***P < 0.001, ****P < 0.0001).

A previous study showed that the deletion of *bpfA* results in a defect in the biofilm maintenance (30 h) (24). Deleted *bpfA* in WT, Δ*crp*, and Δ*lrbR* all failed to form biofilm at 6 h (Fig. 3*A*), indicating that BpfA is the downstream target of the cAMP-CRP and LrbR signaling pathway. Previous studies have shown that the transcription of the *bpfA* operon is regulated by both cAMP-CRP and c-di-GMP (24, 27). The result showed that the *bpfA* transcription of Δ*crp*, Δ*cya*, and Δ*lrbR* was higher than that of WT at 6 h (*SI Appendix*, Fig. S11*A*), and the total BpfA levels in these strains were consistent with *bpfA* transcription (*SI Appendix*, Fig. S11 *B* and *C*), indicating that cAMP-CRP and LrbR regulate the total BpfA levels at the transcriptional level. To determine whether the increase in total BpfA levels is the main reason for the regulation of biofilm formation by cAMP-CRP and LrbR, the native *bpfA* promoter and *bpfA*-specific SD (Shine–Dalgarno) sequence were replaced by a constitutive promoter, P*_aacC1_*, and *aacC1*-specific SD sequence. The results showed that at 6 h, replacement of the promoter and specific SD sequence resulted in similar transcription levels of *bpfA* and total BpfA levels in WT/P*_aacC1_*-*bpfA*, Δ*crp*/P*_aacC1_*-*bpfA*, Δ*cya*/P*_aacC1_*-*bpfA*, and Δ*lrbR*/P*_aacC1_*-*bpfA* (*SI Appendix*, Fig. S12). However, the biofilm biomass of Δ*crp*/P*_aacC1_*-*bpfA*, Δ*cya*/P*_aacC1_*-*bpfA*, and Δ*lrbR*/P*_aacC1_*-*bpfA* was significantly higher than that of WT/P*_aacC1_*-*bpfA* at 6 h (*SI Appendix*, Fig. S13*A*), which was consistent with the biofilm biomass of strains with native promoter and SD sequence (*SI Appendix*, Fig. S13*A*), indicating that the changes in total BpfA levels do not play a decisive role in the regulation of biofilm formation.

A previous study has shown that cell surface localized BpfA levels, not total BpfA levels, control biofilm maintenance (30 h) (24). Now, we found that the cell surface localized BpfA levels of Δ*crp*, Δ*cya*, and Δ*lrbR* were significantly higher than that of WT at 6 h (*SI Appendix*, Fig. S13 *B* and *C*). In addition, replacement of the constitutive promoter resulted in similar total BpfA levels (*SI Appendix*, Fig. S12) but did not affect cell surface localized BpfA levels and therefore had no effect on biofilm formation (*SI Appendix*, Fig. S13), indicating that biofilm biomass is positively correlated with cell surface localized BpfA levels. In *S. putrefaciens* CN32, cell surface localized BpfA levels are controlled by the BpfAGD system (24). The deletion of *bpfD* blocked biofilm formation of all strains, and the deletion of *bpfG* increased biofilm biomass of all strains, including these *bpfD*-deleted strains (Fig. 3*B*). In addition, all *bpfA*-deleted strains failed to form biofilm, including these *bpfG*-deleted strains (Fig. 3*A*). All these results are consistent with the regulatory pattern of the BpfAGD system (23, 24), indicating that the BpfAGD system is the downstream target of the cAMP-CRP and LrbR pathways. In conclusion, cAMP-CRP and LrbR signaling pathway regulate cell surface localized BpfA levels by modulating the BpfAGD system, thereby controlling biofilm formation.

The BpfAGD system responds to intracellular c-di-GMP levels (23, 24). We next sought to investigate whether cAMP-CRP and LrbR regulate the BpfAGD system through the regulation of intracellular c-di-GMP levels. The DgcQ (also known as YedQ) (28), a DGC of *E. coli* MG1655, was expressed in WT, Δ*crp*, Δ*cya*, and Δ*lrbR*. The introduction of DgcQ significantly increased the intracellular c-di-GMP levels of all strains (Fig. 3*C*), but the biofilm biomass of WT/p*dgcQ* was only slightly higher than that of WT (Fig. 3*D*). In addition, although the intracellular c-di-GMP levels increased by the introduction of DgcQ were much higher than those increased by the deletion of *lrbR* (Fig. 3*C*), the biofilm biomass of WT/p*dgcQ* was significantly lower than that of Δ*lrbR* (Fig. 3*D*). Apparently, WT/p*dgcQ* showed some contradictory results, which could not explain the significant increase in biofilm biomass of Δ*crp*, Δ*cya,* and Δ*lrbR* compared to WT as being caused by increased global intracellular c-di-GMP levels. The biofilm biomass of Δ*bpfA*/p*dgcQ*, Δ*bpfD*/p*dgcQ*, and Δ*bpfG*/p*dgcQ* was similar to that of Δ*lrbR*Δ*bpfA*, Δ*lrbR*Δ*bpfD*, and Δ*lrbR*Δ*bpfG*, respectively (Fig. 3*E*). In addition, deletion of genes related to the BpfAGD system in WT/p*dgcQ* had no effect on its intracellular c-di-GMP levels (*SI Appendix*, Fig. S14). These results indicate that the introduction of DgcQ regulates biofilm formation by controlling the BpfAGD system. Co-IP assay of BpfD and BpfG was performed to confirm the above results. Since *bpfD* and *bpfG* were in the same operon as *bpfA* (22, 24, 27) and transcription of the *bpfA* operon was regulated by cAMP-CRP and intracellular c-di-GMP levels (24, 27) (*SI Appendix*, Fig. S11*A*), we used strains containing the *bpfA* operon driven by the constitutive *aacC1* promoter and *aacC1-*specific SD sequence to exclude the influence of total intracellular protein levels (*SI Appendix*, Fig. S12). The interaction between BpfD and BpfG was higher in Δ*crp* and Δ*lrbR* than in WT and WT/p*dgcQ* (Fig. 3*F* and *SI Appendix*, Fig. S15*A*) and the cell surface localized BpfA levels were significantly higher in Δ*crp* and Δ*lrbR* than in WT and WT/p*dgcQ* (Fig. 3*G* and *SI Appendix*, Fig. S16*A*). These results are consistent with the biofilm biomass results (Fig. 3*D*), which also exhibit contradictory results with intracellular c-di-GMP levels (Fig. 3*C*). Specifically, although LrbR and DgcQ regulate global intracellular c-di-GMP levels (Fig. 3*C*) and the BpfAGD system is the downstream target of both LrbR and DgcQ (Fig. 3*E*), the regulatory outcomes are different. The intracellular c-di-GMP levels of Δ*lrbR* were two times higher than WT (Fig. 3*C*), and the interaction between BpfD and BpfG and the cell surface localized BpfA levels of Δ*lrbR* were also higher than that of WT (Fig. 3 *F* and *G* and *SI Appendix*, Fig. S16*A*), resulting in the robust biofilm biomass of Δ*lrbR* (Fig. 3*D*). However, compared to WT, the extremely high intracellular c-di-GMP levels of WT/p*dgcQ* (Fig. 3*C*) did not significantly increase BpfD and BpfG interactions and cell surface localized BpfA levels (Fig. 3 *F* and *G* and *SI Appendix*, Fig. S16*A*), resulting in only a slightly higher increase in biofilm biomass (Fig. 3*D*). These results suggest that although cAMP-CRP and LrbR regulate the biofilm formation by modulating the BpfAGD system, this is achieved by more than just regulating global intracellular c-di-GMP levels.

**Fig. 4.**
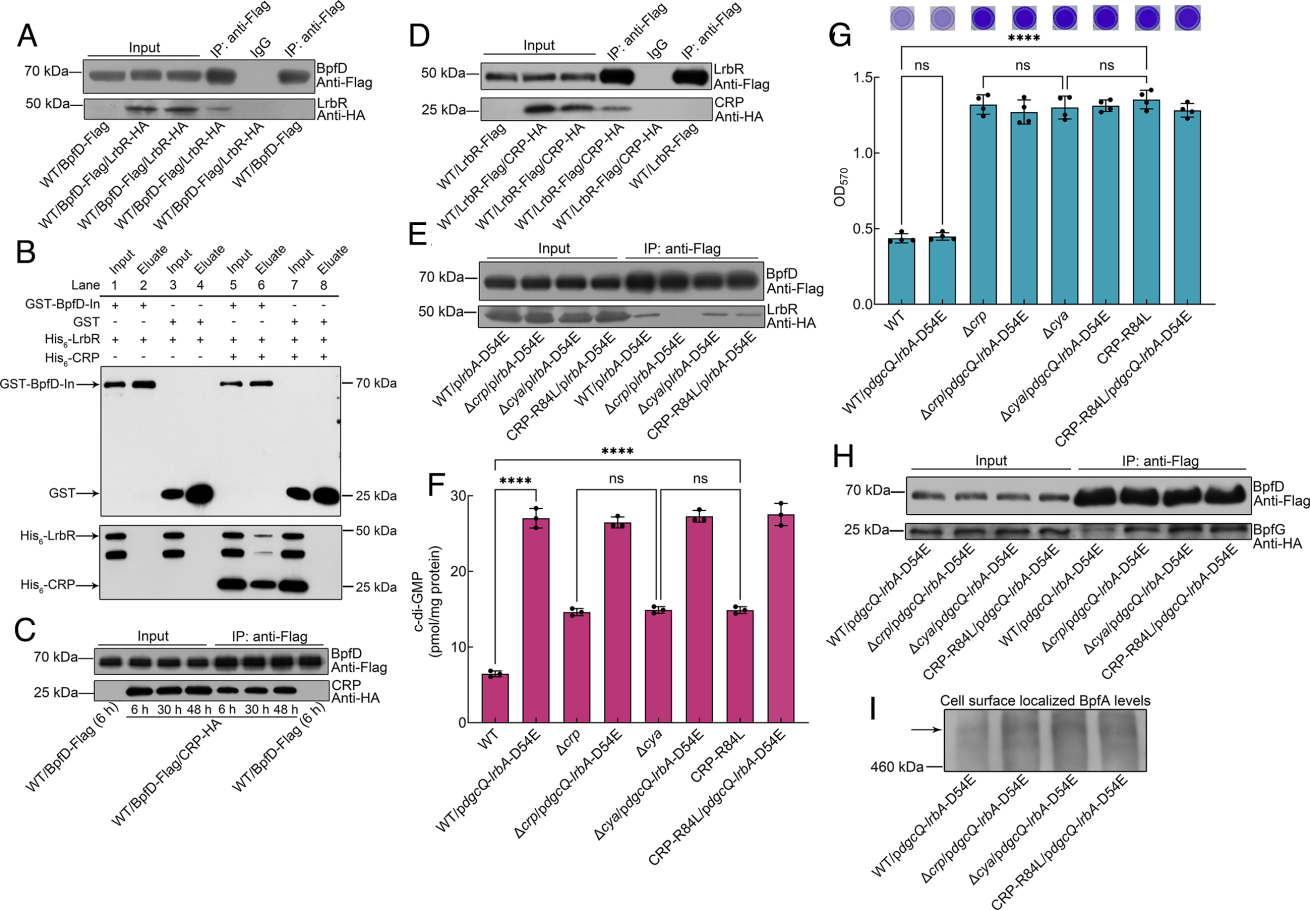
LrbR is involved in the local c-di-GMP signaling pathway through a direct interaction with cAMP-CRP. (*A*) Co-IP to analyze the interaction between BpfD and LrbR in vivo at 6 h. WT/BpfD-Flag was used as a negative control. Co-IP using antibodies against BpfD, LrbR, and IgG negative control. (*B*) GST pull-down assay analyzing the BpfD-LrbR interaction and BpfD-CRP-LrbR interaction in vitro. (*C*) Co-IP to analyze the interaction between BpfD and CRP in vivo at 6, 30, 48 h. WT/BpfD-Flag was used as a negative control. (*D*) Co-IP to analyze the interaction between LrbR and CRP in vivo at 6 h. WT/LrbR-Flag was used as a negative control. Co-IP using antibodies against LrbR, CRP, and IgG negative control. (*E*) Co-IP to analyze the interaction between BpfD and LrbR in vivo at 6 h. 5 μM cAMP was added only to cell lysis of the WT sample. (*F*) Intracellular c-di-GMP concentration at 6 h (n = 3 independent samples). (*G*) Biofilm biomass at 6 h (n = 4 independent samples). Insets are biofilm pictures of crystal violet dyeing. (*H*) Co-IP to analyze the interaction between BpfD and BpfG in vivo at 6 h. 5 μM c-di-GMP was added to cell lysis of all samples. (*I*) Western blotting detection of cell surface localized BpfA levels at 6 h. The strains used in (*A*, *C*, *E*, *H*, and *I*) contain the *bpfA* operon driven by the *aacC1* promoter to exclude the influence of *bpfA*, *bpfD*, *bpfG* transcription caused by the introduction of *dgcQ* or the overexpression of LrbA-D54E or the deletion of *cya* and *crp* or the replacement of CRP-R84L. All strains used in (*A* and *C*–*I*) were cultured in MM1 medium. Data in (*F* and *G*) are shown as the mean ± SD. One-way ANOVA followed by Tukey’s multiple comparison tests was used in (*F* and *G*) to analyze the statistical significance, which was provided by GraphPad Prism 10 statistical software (ns: no significance, ****P < 0.0001).

In *P. fluorescens* Pf0-1, the Lap system responds not only to global c-di-GMP signaling but also to local c-di-GMP signaling (21, 29, 30). The c-di-GMP metabolic enzymes involved in local c-di-GMP signaling directly interact with the c-di-GMP effectors to control the downstream phenotype but do not significantly affect global intracellular c-di-GMP levels (31). Thus, we next sought to investigate whether the above contradictory result was due to the fact that LrbR is involved in the local c-di-GMP signaling to regulate the BpfAGD system. Although LrbR interacts with BpfD in vivo (Fig. 4*A*), no direct interaction between BpfD and LrbR was detected by GST pull-down assay (Fig. 4*B*, Lane 2). The CRP-BpfD interaction occurs at 6 h, 30 h, and 48 h in vivo (24) (Fig. 4*C* and *SI Appendix*, Fig. S15*B*), suggesting that the interaction is maintained throughout the biofilm development. GST pull-down and Co-IP results showed that CRP directly interacted with LrbR (Fig. 4*D* and *SI Appendix*, Fig. S17 *A*), suggesting that the interaction between LrbR and BpfD may be indirect and that CRP may contribute to this interaction. The results of a GST pull-down assay of the three proteins showed that LrbR was detected in the eluate after the addition of CRP to the mixture of BpfD and LrbR (Fig. 4*B*, Lane 6), indicating that both BpfD and LrbR interact with CRP, forming a LrbR–CRP–BpfD ternary complex in vitro. We next sought to demonstrate this result in vivo. Since cAMP-CRP was essential for *lrbR* transcription, intracellular LrbR protein levels were particularly low in the *crp*-deleted mutants (Fig. 2 *A* and *B*). We used the strains overexpressing LrbA-D54E, which has an increase in intracellular LrbR proteins in the absence of CRP (Fig. 2*G*). The Co-IP results showed that LrbR was not detected in the eluate of the *crp*-deleted mutant (Fig. 4*E*), indicating that BpfD cannot form a stable complex with LrbR in the absence of CRP, but can form a stable complex in the presence of CRP.

The question was raised whether the lower biofilm biomass of WT/p*dgcQ* compared to Δ*lrbR* was caused by the formation of the LrbR–CRP–BpfD ternary complex. Thus, we next sought to investigate whether LrbR exerts local c-di-GMP signaling through the formation of the LrbR–CRP–BpfD ternary complex. Overexpression of LrbA-D54E in WT and Δ*crp* resulted in higher intracellular LrbR levels (Fig. 2*G*). It is noteworthy that high intracellular LrbR levels led to significantly reduced intracellular c-di-GMP levels in both WT and Δ*crp* (Fig. 2 *G* and *H*), resulting in a significant decrease in biofilm biomass in both strains (Fig. 2*I*). This suggests that the high intracellular LrbR levels can exert their global c-di-GMP signaling in both WT and Δ*crp*. A previous study has shown that global and local c-di-GMP signaling are not antagonistic regulatory models (32). If the amount of a c-di-GMP metabolic enzyme involved in local signaling is large enough or its enzyme activity is high enough, which can lead to the changes in global intracellular c-di-GMP levels, the c-di-GMP metabolic enzyme regulates not only local c-di-GMP signaling but also global c-di-GMP signaling (32). Thus, we speculated that LrbA-D54E overexpression results in increased intracellular LrbR levels, which regulates not only local c-di-GMP signaling but also global c-di-GMP signaling. To confirm this speculation and exclude the global c-di-GMP signaling caused by LrbR, we expressed both *dgcQ* and *lrbA*-D54E to obtain WT/p*dgcQ*-*lrbA*-D54E and Δ*crp*/p*dgcQ*-*lrbA*-D54E. On the one hand, overexpression of LrbA-D54E increased the expression of *lrbR* on the genome through regulation of transcription (Fig. 2*F*), leading to an increased intracellular LrbR levels (*SI Appendix*, Fig. S17*B*). On the other hand, although increased LrbR levels could lead to a decrease in global intracellular c-di-GMP levels (Fig. 2*H*), total intracellular c-di-GMP levels were greatly increased by overexpressing *dgcQ* on the plasmid (Fig. 4*F*), which suppressed the decrease in global c-di-GMP levels caused by LrbR. Finally, we obtained the strains with increased global intracellular c-di-GMP levels and intracellular LrbR levels (Fig. 4*F* and *SI Appendix*, Fig. S17*B*). Specifically, both intracellular c-di-GMP levels and intracellular LrbR levels of WT/p*dgcQ*-*lrbA*-D54E and Δ*crp*/p*dgcQ*-*lrbA*-D54E were significantly higher than those of WT and Δ*crp*, respectively (Fig. 4*F* and *SI Appendix*, Fig. S17*B*), indicating that the introduction of DgcQ significantly increases the global intracellular c-di-GMP levels but does not affect the increased intracellular LrbR levels due to LrbA-D54E overexpression. In addition, although the global intracellular c-di-GMP levels of WT/p*dgcQ*-*lrbA*-D54E were not only significantly higher than those of WT but also those of Δ*crp* (Fig. 4*F*), the biofilm biomass of WT/p*dgcQ*-*lrbA*-D54E was similar to that of WT and significantly lower than that of Δ*crp* (Fig. 4*G*). Furthermore, although the global intracellular c-di-GMP levels of WT/p*dgcQ*-*lrbA*-D54E were similar to those of Δ*crp*/p*dgcQ*-*lrbA*-D54E (Fig. 4*F*), the biofilm biomass of WT/p*dgcQ*-*lrbA*-D54E was significantly lower than that of Δ*crp*/p*dgcQ*-*lrbA*-D54E (Fig. 4*G*). The BpfD-BpfG interaction and cell surface localized BpfA levels in WT/p*dgcQ*-*lrbA*-D54E were lower than that of Δ*crp*/p*dgcQ*-*lrbA*-D54E (Fig. 4 *H* and *I* and *SI Appendix*, Fig. S16*B*), which consistent with the biofilm biomass of these strains (Fig. 4*G*). These results indicate that the formation of the LrbR–CRP–BpfD ternary complex allows LrbR to exert local c-di-GMP signaling, which is the reason why elevated global intracellular c-di-GMP levels in WT/p*dgcQ*-*lrbA*-D54E have no effect on biofilm biomass.

CRP recruits LrbR to BpfD, allowing LrbR to exert local c-di-GMP signaling, but does this regulatory process still require the involvement of cAMP? CRP still interacted with LrbR in the absence of cAMP in vitro (*SI Appendix*, Fig. S17*A*). In addition, the CRP-R84L, a mutant protein of CRP that cannot bind cAMP (24), not only directly interacted with LrbR (*SI Appendix*, Fig. S17*C*) but also mediated LrbR interactions with BpfD (*SI Appendix*, Fig. S17*D*). These results indicate that the absence of cAMP does not affect the interaction between CRP and LrbR in vitro. Since the intracellular LrbR levels were significantly reduced in Δ*cya* or CRP-R84L (*SI Appendix*, Fig. S17*B*), Δ*cya*/p*dgcQ*-*lrbA*-D54E and CRP-R84L/p*dgcQ*-*lrbA*-D54E were used for Co-IP assays. The results showed that not only CRP-R84L interacted with LrbR, but also CRP interacted with LrbR in the absence of cAMP in vivo (*SI Appendix*, Fig. S17*E*), indicating that cAMP is not necessary for the physical interaction between CRP and LrbR.

During the biofilm maturation stage, the absence of cAMP does not affect the interaction between CRP and BpfD but causes CRP to lose its regulatory function (24). Specifically, the absence of cAMP leads to the interaction of CRP-BpfD failing to enhance the binding of BpfG to BpfD (24). We next sought to investigate whether the absence of cAMP affects the local regulatory function of the LrbR. The Co-IP results showed that LrbR was not detected in the eluate of the Δ*crp*/p*dgcQ-lrbA-*D54E but can be detected in Δ*cya*/p*dgcQ-lrbA-*D54E and CRP-R84L/p*dgcQ-lrbA-*D54E (*SI Appendix*, Fig. S17*F*), indicating that the absence of cAMP did not affect the formation of the LrbR–CRP–BpfD ternary complex. However, the intracellular c-di-GMP levels, biofilm biomass, BpfD-BpfG interaction, and the cell surface BpfA levels of Δ*cya*/p*dgcQ-lrbA-*D54E and CRP-R84L/p*dgcQ-lrbA-*D54E were similar to those of Δ*crp*/p*dgcQ-lrbA-*D54E (Fig. 4 *F*−*I* and *SI Appendix*, Fig. S16*B*), indicating that although the absence of cAMP does not influence the interaction between CRP and LrbR, it leads to the loss of local regulation of LrbR. Thus, CRP, LrbR, and BpfD can form a stable complex, which does not require the presence of cAMP. However, the absence of cAMP causes LrbR to lose its local regulatory function. In conclusion, the LrbR is involved in a local c-di-GMP signaling pathway through its interaction with cAMP-CRP.

cAMP-CRP supports the biofilm maintenance at 30 h (24). Since CRP interacts with BpfD at both 6 and 30 h (Fig. 4*C*), why does LrbR no longer provide local c-di-GMP signaling to negatively regulate biofilm biomass at 30 h? The *lrbR* transcription was significantly reduced at 30 h compared to 6 h (*SI Appendix*, Fig. S18*A*), LrbR protein was barely detected in the cells at 30 h (*SI Appendix*, Fig. S18*B*), and the biofilm biomass of Δ*lrbR* was similar to WT at 30 h (*SI Appendix*, Fig. S18*C*). A previous study showed that the *lrbR* transcription responds to the lactate concentration in MM1 medium (26). These results showed that LrbR was absent from cells (*SI Appendix*, Fig. S18 *A* and *B*), when lactate was depleted at 30 h (*SI Appendix*, Fig. S18*D*). Consequently, the BpfAGD system cannot receive local c-di-GMP signaling. Therefore, as intracellular LrbR levels decrease, cAMP-CRP-BpfD enables a rapid shift to biofilm development and supports biofilm maintenance (Fig. 1A). The issue here is that once LrbR has decreased, the enhancement of the BpfAGD system could result from cAMP-CRP interacting with BpfD. Alternatively, it could be due to local regulation by other DGCs recruited by cAMP-CRP. This should be investigated in future studies.

### Cross-Regulation of cAMP-CRP and c-di-GMP Enables a Rapid Shift to Biofilm Development in Nutrient-Poor Conditions.

**Fig. 5.**
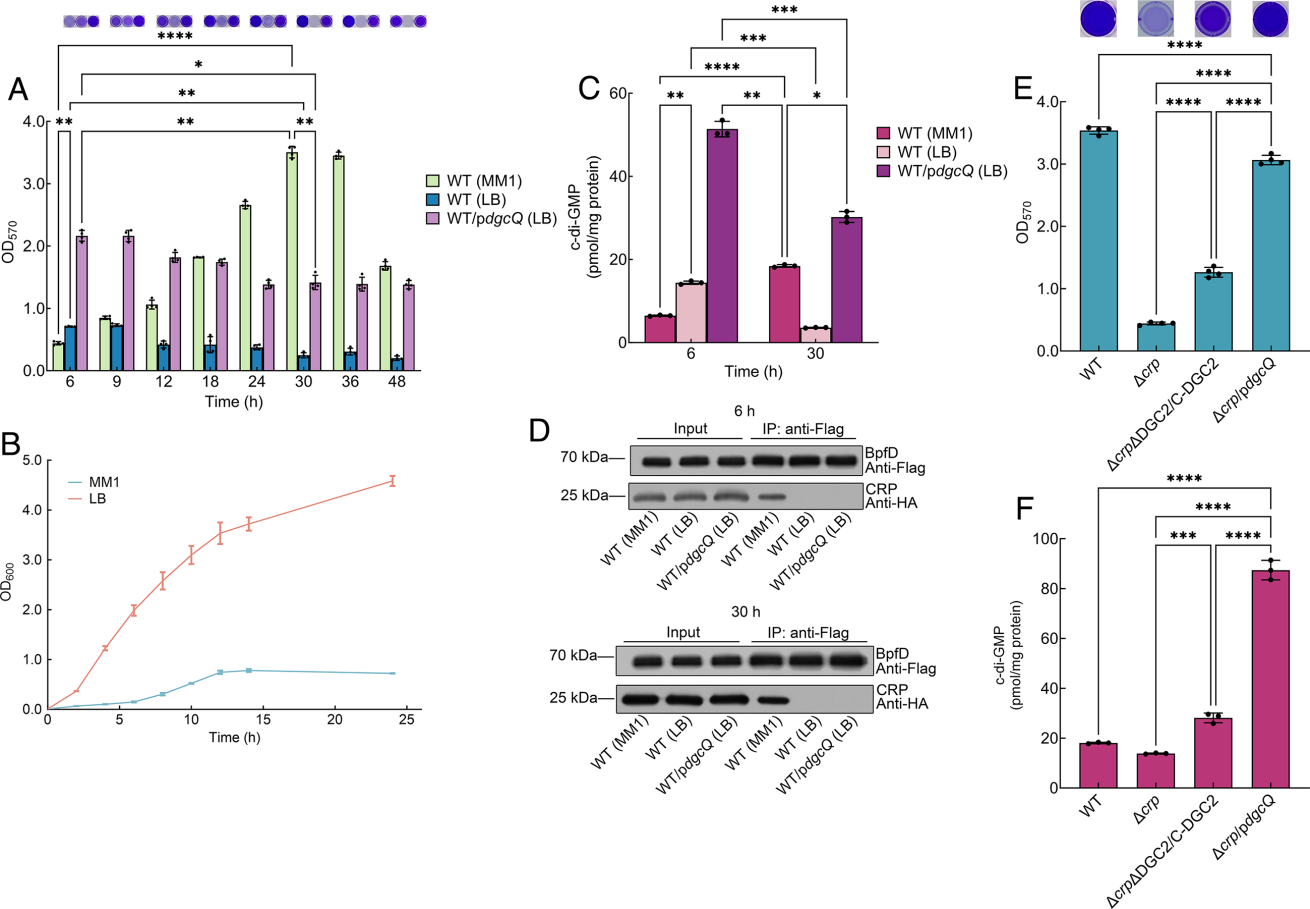
Cross-regulation of cAMP-CRP and c-di-GMP enables a rapid shift to biofilm development in nutrient-poor conditions. (*A*) Biofilm biomass of WT or WT/p*dgcQ* in MM1 medium or LB medium (n = 4 independent samples). (*B*) Planktonic cell growth of WT in MM1 medium or LB medium (n = 3 independent samples). (*C*) Intracellular c-di-GMP concentration of WT or WT/p*dgcQ* in MM1 medium or LB medium (n = 3 independent samples). (*D*) Co-IP to analyze the interaction between BpfD and CRP in vivo at 6 and 30 h in MM1 medium or LB medium. The strains contain the *bpfA* operon driven by the *aacC1* promoter to exclude the influence of *bpfD* transcription caused by the introduction of *dgcQ* or different medium. (*E*) Biofilm biomass of Δ*crp*-related strains in MM1 medium at 30 h (n = 4 independent samples). (*F*) Intracellular c-di-GMP concentration of Δ*crp*-related strains in MM1 medium at 30 h (n = 3 independent samples). Insets in (*A* and *E*) are the biofilm pictures of crystal violet dyeing. Data in (*A*–*C*, *E*, and *F*) are shown as the mean ± SD. Two-way ANOVA (*A* and *C*) and one-way ANOVA (*E* and *F*) followed by Tukey’s multiple comparison tests was used to analyze the statistical significance, which was provided by GraphPad Prism 10 statistical software (*P < 0.05, **P < 0.01, ***P < 0.001, ****P < 0.0001).

We found that when bacteria form biofilm in MM1 medium (nutrient-poor medium), the interaction between cAMP-CRP and BpfD is maintained throughout the biofilm development (Fig. 4*C*). What is the biological significance of this interaction? *S. putrefaciens* CN32 form biofilm not only in MM1 medium but also in LB medium (nutrient-rich medium) (24, 27). The biofilm biomass of LB-cultured WT was significantly lower than that of MM1-cultured WT at most time points, with the exception of 6 h (Fig. 5*A*). However, the cell growth of planktonic bacteria in LB was significantly higher than in MM1 (Fig. 5*B*). We used WT/p*dgcQ* for better biofilm formation in LB. The results showed that at all time points, the intracellular c-di-GMP levels and biofilm biomass in LB was significantly increased by the introduction of DgcQ (Fig. 5 *A* and *C*), indicating that when cultured in LB, the biofilm formation of *S. putrefaciens* CN32 was also regulated by global intracellular c-di-GMP levels. However, the regulation of biofilm formation by c-di-GMP differs in LB and MM1 media. The intracellular c-di-GMP levels and the biofilm biomass of LB-cultured WT/p*dgcQ* were both more than three times higher than those of LB-cultured WT at 6 h, and both were more than seven times higher at 30 h (Fig. 5 *A* and *C*). In addition, the intracellular c-di-GMP levels and biofilm biomass of LB-cultured WT decreased more than threefold at 30 h compared to 6 h, whereas both decreased about 1.5-fold in LB-cultured WT/p*dgcQ* (Fig. 5 *A* and *C*). These results indicate that a positive correlation between biofilm biomass and intracellular c-di-GMP levels were observed when bacteria were cultured in LB. However, the situation is different when bacteria are cultured in MM1. Although the global intracellular c-di-GMP levels of MM1-cultured WT/p*dgcQ* were more than four times higher than those of MM1-cultured WT at 30 h, the biofilm biomass of both MM1-cultured bacteria was similar (*SI Appendix*, Fig. S19). Most importantly, the intracellular c-di-GMP levels of MM1-cultured WT only increased about twofold at 30 h compared to 6 h, but the biofilm biomass of MM1-cultured WT increased about sixfold at 30 h compared to 6 h (Fig. 5 *A* and *C*). In addition, the intracellular c-di-GMP levels of LB-cultured WT/p*dgcQ* were more than 1.5 times higher than those of MM1-cultured WT at 30 h (Fig. 5*C*), but the biofilm biomass of LB-cultured WT/p*dgcQ* was only half of that of MM1-cultured WT (Fig. 5*A*). These results suggest that a moderate increase in intracellular c-di-GMP levels results in a rapid increase in biofilm biomass in MM1; however, a positive correlation was observed between biofilm biomass and intracellular c-di-GMP levels in LB. Thus, we speculate that certain factors contribute to the rapid shift of biofilm development when the bacteria are cultured in MM1. The Co-IP assay showed that no interaction between CRP and BpfD was detected at 6 h or 30 h in either LB-cultured WT or WT/p*dgcQ* (Fig. 5*D*), suggesting that cAMP-CRP and BpfD can only interact in MM1. All three proteins of BpfAGD system were expressed despite differences in total BpfA/BpfG/BpfD protein levels between bacteria cultured in MM1 and LB (*SI Appendix*, Fig. S20*A*), and CRP is highly expressed when both bacteria are cultured in MM1 and LB at either 6 h or 30 h (*SI Appendix*, Fig. S20*B*). In addition, the intracellular cAMP levels of LB-cultured bacteria were two times higher than those of MM1-cultured bacteria at both 6 and 30 h (*SI Appendix*, Fig. S20*C*). All these results indicate that the BpfAGD system and CRP can be expressed, and the cAMP levels are also sufficient for the regulatory function of CRP when the bacteria are cultured in LB medium. Thus, the failure to detect CRP interaction with BpfD in LB was not due to the absence of protein production. In both LB-cultured WT and WT/p*dgcQ*, the biofilm biomass of both bacteria was significantly decreased as *bpfA* and *bpfD* deleted and significantly increased as *bpfG* deleted at both 6 and 30 h (*SI Appendix*, Fig. S20*D*), indicating that the BpfAGD system still plays an important role in controlling the biofilm formation in LB. In conclusion, when bacteria are cultured in MM1, a twofold increase in intracellular c-di-GMP levels from 6 to 30 h results in a sixfold increase in biofilm biomass due to the interaction between cAMP-CRP and BpfD. The failure of cAMP-CRP and BpfD interactions in LB leads to the positive correlation between c-di-GMP and biofilm biomass, indicating that high global intracellular c-di-GMP levels are required to maintain high biofilm biomass in the absence of (cAMP-)CRP-BpfD interactions. To further confirm this conclusion, we analyzed the regulatory connection between intracellular c-di-GMP levels and biofilm biomass in MM1-cultured Δ*crp* at 30 h. The *dgcQ* gene and two other DGC genes [Sputcn32_1291 and Sputcn32_3328 (24), abbreviated to DGC2 here] were introduced into the Δ*crp* strain to increase global intracellular c-di-GMP levels. The results showed that the biofilm biomass was positively correlated with intracellular c-di-GMP levels after deletion of *crp* (Fig. 5 *E* and *F*), indicating that high global intracellular c-di-GMP levels are required to overcome the loss of *crp* and support biofilm formation in MM1. Thus, the rapid shift to biofilm development under nutrient-poor conditions is driven by cAMP-CRP and its cross-regulation with c-di-GMP.

## Discussion

**Fig. 6.**
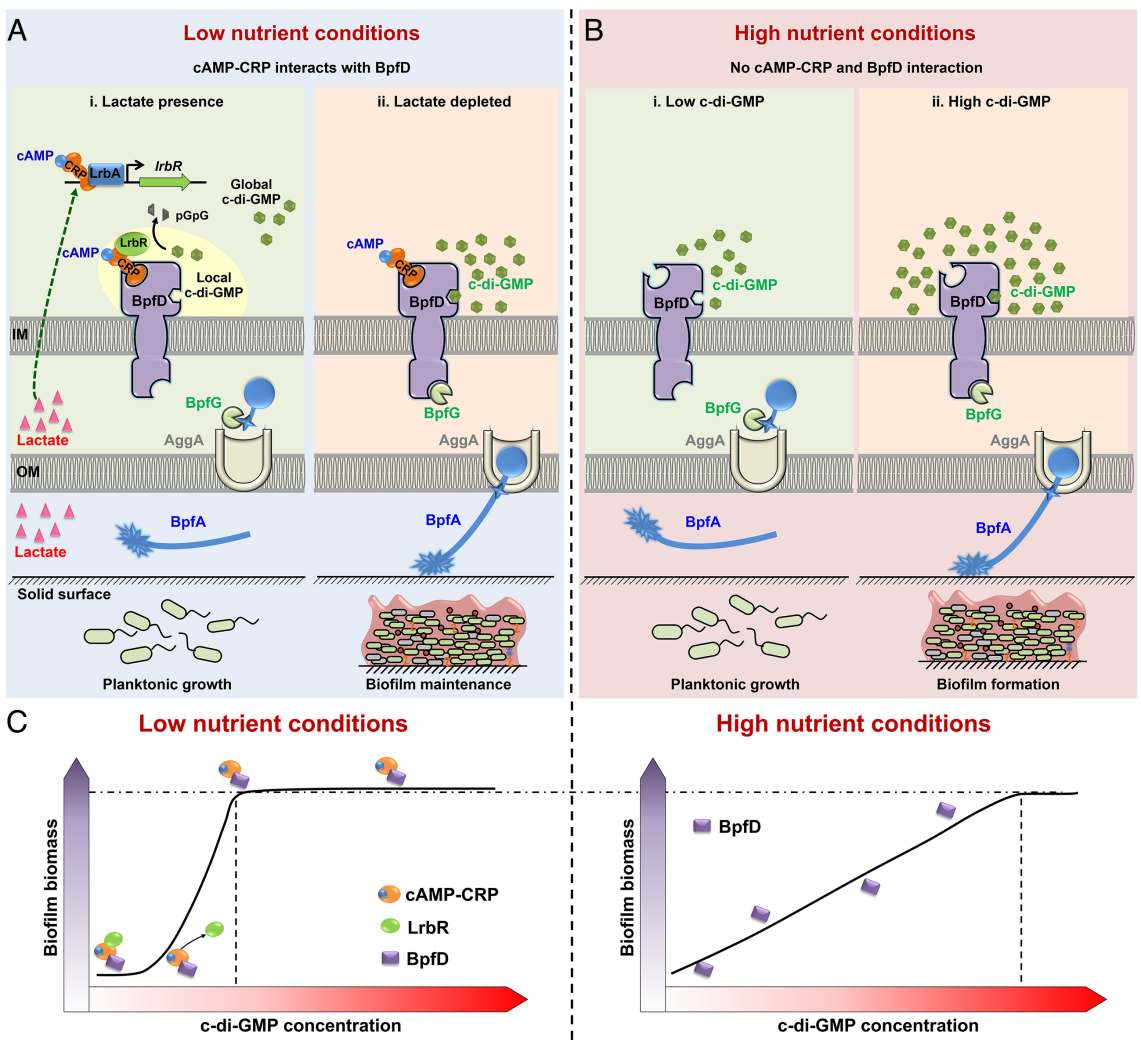
Pattern for the regulation under different nutrient conditions. (*A*) Cross-regulation of cAMP-CRP and c-di-GMP occurs under low nutrient conditions (MM1 medium). i) In the presence of lactate, cAMP-CRP directly promotes the transcription of a PDE gene, *lrbR*, by LrbA. Additionally, cAMP-CRP recruits LrbR to BpfD to suppress the early biofilm formation via LrbR-dependent local degradation of c-di-GMP. ii) As intracellular LrbR levels decrease in response to depleted lactate, cAMP-CRP-BpfD enables a rapid shift to biofilm development and supports biofilm maintenance. The yellow oval background region indicates the local c-di-GMP signaling exerted by LrbR. (*B*) Under high nutrient conditions, cAMP-CRP does not interact with BpfD. i) Low global intracellular c-di-GMP levels prevent BpfD from interacting with BpfG, allowing BpfG to process and release BpfA from the cell surface, leading to planktonic growth. ii) High intracellular c-di-GMP levels promote the formation of the c-di-GMP-BpfD complex, which interacts with BpfG and sequesters BpfG on the inner membrane, resulting in the location of BpfA on the outer membrane, thereby promoting biofilm formation. IM indicates the inner membrane, and OM indicates the outer membrane. The dashed arrows indicate indirect regulation. (*C*) The relationship between biofilm biomass and global intracellular c-di-GMP concentration under different nutrient conditions. The horizontal arrow indicates increasing global intracellular c-di-GMP concentration (also indicated with the gradient of the red panels in the background); the vertical arrow indicates increasing biofilm biomass (also indicated with the gradient of the purple panels in the background).

We proposed the regulatory pattern shown in Fig. 6. Under nutrient-poor conditions with sufficient lactate, cAMP-CRP directly promotes the transcription of *lrbR* by LrbA. Additionally, cAMP-CRP recruits LrbR to BpfD to suppress early biofilm formation via LrbR-dependent local degradation of c-di-GMP (Fig. 6 *A*, *i*). As lactate is exhausted, intracellular LrbR levels decrease significantly, enabling a rapid shift to biofilm development and supporting biofilm maintenance via cAMP-CRP-BpfD (Fig. 6 *A*, *ii*). Under high nutrient conditions, the cross-regulation of cAMP-CRP and LrbR and BpfD does not occur, and the BpfAGD system responds to global intracellular c-di-GMP levels (Fig. 6*B*). Fig. 6*C* illustrates the correlation between biofilm biomass and global intracellular c-di-GMP levels under different nutrient conditions. Under low nutrient conditions, a moderate increase in intracellular c-di-GMP levels results in a rapid increase in biofilm biomass, which is dependent on the cross-regulation of cAMP-CRP and c-di-GMP signaling. Under high nutrient conditions, a positive correlation was observed between global intracellular c-di-GMP levels and biofilm biomass. This regulation means that bacteria need to maintain high levels of intracellular c-di-GMP to support a robust biofilm in nutrient-rich environments. On the one hand, this mechanism prevents biofilm formation from being readily induced in nutrient-rich environments. Even if some stress signals occur transiently in the bacterial ecological niche, unless they lead to a significant increase in intracellular c-di-GMP levels, bacteria still grow planktonically and proliferate rapidly in nutrient-rich environments. On the other hand, once certain stress signals are present, which greatly increase intracellular c-di-GMP levels, robust biofilm formation can be induced to protect the bacteria from adverse stress. This regulatory mechanism ensures that the bacteria live primarily on a lifestyle of planktonic growth under nutrient-rich conditions. Although planktonic growth strains are less resistant to stress than strains that form biofilms, rapid growth and proliferation to produce large numbers of bacteria will ensure survival even under certain stresses (except in the case of severe stress that leads to large numbers of bacteria dying). The regulatory direction of bacterial lifestyle under the nutrient-poor conditions is opposite to that under the nutrient-rich conditions. Generally, the high adaptive potential of bacteria enables them to grow planktonically and proliferate even under nonoptimal or resource-poor environmental conditions (33). Until resources become exhausted and/or stress levels increase, bacteria trigger a fundamental change in lifestyle, from a quantitative to a qualitative strategy (6, 33, 34). The cross-regulation between cAMP-CRP and c-di-GMP signaling not only promotes bacteria growing planktonically under nutrient-poor conditions with optimal carbon sources but also promotes them quickly forming a biofilm as the nutrient environment becomes more hostile. In conclusion, *S. putrefaciens* CN32 has evolved a more efficient strategy to form biofilms under different nutrient conditions.

Although the BpfAGD system is controlled by c-di-GMP signaling and regulates biofilm development in LB and MM1 media, there are still major differences. In MM1, the biofilm biomass of Δ*bpfG* and Δ*bpfG*/p*dgcQ* was similar either at 6 h or 30 h (Fig. 3*E* and *SI Appendix*, Fig. S20*E*). This indicates that after the deletion of *bpfG*, an increase in global intracellular c-di-GMP levels failed to increase biofilm biomass when the bacteria were cultured under nutrient-poor conditions. However, the biofilm biomass of Δ*bpfG*/p*dgcQ* was significantly higher than that of Δ*bpfG* in LB either at 6 h or 30 h (*SI Appendix*, Fig. S20*D*). This suggests that after the deletion of *bpfG*, an increase in global intracellular c-di-GMP levels can lead to an increase in biofilm biomass when bacteria are cultured under nutrient-rich conditions. This may be because the synthesis or secretion of other extracellular matrix components responds to global intracellular c-di-GMP levels in addition to BpfA under nutrient-rich conditions. The reason that *bpfA* deletion strains are unable to form a biofilm under the nutrient-rich conditions (*SI Appendix*, Fig. S20*D*) could be because this unknown component works together with BpfA. The positive correlation between c-di-GMP and biofilm biomass in nutrient-rich conditions is the result of the BpfAGD system and the unknown extracellular matrix components. This should be further investigated in future studies.

The absence of cAMP did not affect the interaction between CRP-LrbR and CRP-BpfD (Fig. 4*D* and *SI Appendix*, Fig. S17) (24), but it did affect the regulatory function of CRP (Fig. 4 *F* and *G*). If CRP merely enables LrbR to reduce the concentrations of c-di-GMP adjacent to BpfD, then the formation of the LrbR-CRP-BpfD complex appears to be sufficient. Why does this regulatory process require the involvement of cAMP? Perhaps in addition to spatial proximity, complex conformation is also important. In some bacteria, such as *Mycobacterium tuberculosis*, CRP can bind to DNA in the absence of cAMP (35). However, in most cases, especially in γ-proteobacteria, only cAMP-bound CRP can bind DNA (7, 36, 37). Thus, we speculate that although the absence of cAMP did not affect the formation of the LrbR–CRP–BpfD ternary complex, either the conformation of cAMP-CRP or the formation of the cAMP–CRP–DNA ternary complex is the key factor for CRP to exert its regulatory function, which requires investigation in future studies.

In bacteria, cAMP-CRP acts as a global regulator mainly at the transcriptional level (25), but recently it has been found to be involved in posttranslational regulation (7, 24, 38). In *S. putrefaciens* CN32, the posttranslational cross-regulation interaction occurs not only between the cAMP receptor protein (CRP) and the c-di-GMP PDE (LrbR), but also between CRP and the c-di-GMP effector (BpfD). cAMP and c-di-GMP are widely found in bacteria (7). The amino acid sequence of *S. putrefaciens* CN32 CRP exhibits 88% identity with *E. coli* and *V. cholerae* CRP. Proteins containing the GGDEF and EAL domains are generally present as c-di-GMP metabolic enzymes or effectors, which are widely found in bacteria (16). Thus, this cross-regulation may be common in a wide range of bacteria. In addition, cAMP-CRP responds to nutrient cues (9). c-di-GMP regulates not only biofilm development, but also cell cycle, antibiotic biosynthesis, and many other physiological functions (7, 16). Thus, such cross-regulation may exist in the regulation of many physiological processes in addition to biofilm development. Our research model provides guidance for studying the cross-regulation of multiple second messengers and how such regulation controls physiological processes in response to nutrient cues in other bacteria.

## Materials and Methods

### Bacterial Strains and Growth Conditions.

*E. coli* strains were grown at 37 °C in Luria-Bertani (LB) medium. The LB broth and modified M1 medium [MM1: containing 30 mM HEPES, 1.34 mM KCl, 28.04 mM NH_4_Cl, 4.35 mM NaH_2_PO_4_, 7.5 mM NaOH, adjusted to pH 7.0, supplemented with 20 mM sodium lactate, 0.68 mM CaCl_2_, and trace amounts of amino acids, minerals, and vitamins (26)] were used for the biofilm formation of *S. putrefaciens* CN32 WT and its derivative strains. When necessary, the medium was supplemented with 50 μg/mL of kanamycin for *S. putrefaciens* CN32 and 100 μg/mL of ampicillin or 50 μg/mL of kanamycin for *E. coli*. The LB broth and the MM1 medium (vortex for 1 min to disrupt the aggregates before measuring OD_600_) were used for the cell growth of *S. putrefaciens* CN32 and its derivatives. The seeds of *S. putrefaciens* CN32 and its derivatives were grown in LB medium. Specifically, 10 mL LB medium was prepared in the 50-mL centrifuge tube (NEST, China) (supplemented with 50 μg/mL of kanamycin when necessary). To ensure that all strains were in a consistent status, bacteria preserved in 25% glycerol at −80 °C were picked and inoculated into the prepared liquid LB medium, which were cultured for 21 h at 30 °C, 200 rpm. The strains and plasmids used in this study are listed in *SI Appendix*, Table S1, and the primers are listed in *SI Appendix*, Table S2. The related reagents used in this study are listed in *SI Appendix*, Table S3. Additional detailed descriptions of the *Materials and Methods* can be found in *SI Appendix*.

## Supplementary Material

Appendix 01 (PDF)

## Data Availability

All study data are included in the article and/or *SI Appendix*.
