# Supplementary material for: Local c-di-GMP signaling, triggered by cross-regulation of cAMP-CRP and c-di-GMP, controls biofilm formation under nutrient limitation
Source: Proc Natl Acad Sci U S A. 2025 Aug 25;122(35):e2516964122. doi: 10.1073/pnas.2516964122 (PMC12415220; doi:10.1073/pnas.2516964122)
Supplement: Supplementary file 1 — Appendix 01 (PDF) [file pnas.2516964122.sapp.pdf]

PNAS

Supporting Information for

Local c-di-GMP signaling, triggered by cross-regulation of cAMP-CRP and c-di-GMP, controls biofilm formation under nutrient limitation

Di Sun[a,1], Xiaobo Liu[b,1], Ying Zhang[c,1], Rui Shi[a], Yunrui Ru[a], Xuge Zhou[a], Ying Chen[a], Jing Yang[a], Jiawen Liu[a], Jingrong Zhu[a], Cong Liu[a,2], and Weijie Liu[a,2]

[a]Department of Microbiology, School of Life Sciences, Jiangsu Normal University, Xuzhou 221116, China.

[b]Department of Biological Engineering, School of Environmental and Biological Engineering, Nanjing University of Science and Technology, Nanjing 210094, China.

[c]Department of Space Biology Research, School of Life Science, Beijing Institute of Technology, Beijing 100081, China.

[1]These authors contributed equally: Di Sun, Xiaobo Liu, Ying Zhang

[2]Corresponding: Cong Liu, Tel: +86 18610712643, E-mail: liucong0426@126.com; Weijie Liu, Tel: +86 18252161264, E-mail: leonliu2013@126.com

This PDF file includes:

Supporting Material and Methods

Fig. S1 to S20

Tables S1 to S4

SI References

Supporting Material and Methods

Construction of *S. putrefaciens* CN32 deletion mutants and complementation strains

In-frame deletion mutants were generated as described previously (1). For example, to construct the *Sputcn32_1235* deletion mutant, a 922-bp upstream fragment (–814 to +108 bp relative to the *Sputcn32_1235* start codon) and a 961-bp downstream fragment (+1726 to +2687 bp relative to the *Sputcn32_1235* start codon) were amplified using (primer pairs D*1235*-5F/D*1235*-5R and D*1235*-3F/D*1235*-3R, respectively). The homologous arms were digested by *Eco*RI/*Bam*HI and *Bam*HI/*Pst*I, and both were cloned into the *Eco*RI/*Pst*I-digested vector pK19*mobsacB*(2) to yield pK19-*1235*UD. The pK19-*1235*UD was transformed into *S. putrefaciens* CN32 by conjugation. The Δ*1235* deletion mutant was verified by PCR, using the following primer pairs D*1235*-UF/D*1235*-DR, D*1235*-OF/D*1235*-DR, D*1235*-UF/D*1235*-OR, and D*1235*-INF/D*1235*-INR. A similar strategy was used to generate all relative deletion mutants in the present study. All of the resulting mutants were verified by PCR and DNA sequencing.

The complementation strains were generated using plasmid pBBR1MCS-2-P_{aacC1} (sourced from pBBR1MCS-2) as described previously(3, 4). For example, to complement Δ*1235*, DNA fragments carrying the ribosome binding site (RBS) and complete ORFs of the *Sputcn32_1235* genes without their own promoters were amplified using primers *1235*-comF/*1235*-comR. Then the DNA fragments were cloned into the *Bam*HI and *Eco*RI sites of pBBR1MCS-2-P_{aacC1}(4), thereby yielding the complementation vectors pBBR-P_{aacC1}-*1235*. The coding region of *Sputcn32_1235* with its own RBS was under the control of an *aacC1* promoter. The complementation plasmid was transformed into Δ*1235* by conjugation. A similar strategy was used to generate all relative complementation strains in the present study. All of the complementation strains were verified by PCR and DNA sequencing.

Construction of 3×Flag or 1×HA tagged transformant

All tags were knocked into the corresponding location of the labeled gene in the genome. For example, to construct a C-terminal 3×Flag-tagged LrbR transformant, the 3'-terminus region of the *lrbR* gene, including its upstream and downstream flanks was amplified by the primers *lrbR*-Flag/HA-ConF and *lrbR*-Flag/HA-ConR, which was cloned into pK19*mobsacB* to yield an intermediate plasmid pK19-LrbR-CterUD. The pK19-LrbR-CterUD was linearized by PCR amplification using the primers *lrbR*-Flag-KinF and *lrbR*-Flag-KinR (5' phosphorylated primer), and the yielding fragment was digested by *Bam*HI, defined as pK19-LrbR-CterUD-*Bam*HI. pMV-Flag was a template to amplified DNA fragment of 3×Flag by primers Flag-F (5' phosphorylated primer) and Flag-R. The yielding fragment was digested with *Bam*HI and cloned into pK19-LrbR-CterUD-*Bam*HI to yield the LrbR C-terminal 3×Flag-tag knock-in plasmid, pK19-LrbR-Cter-FlagKin, which was conjugated into related strains. Thus, the 3×Flag nucleotide

sequence was knocked into the *lrbR* in-frame of the genome by homologous recombination to obtain related LrbR-Flag strains. The final transformant was verified by PCR, using the following primer pairs *lrbR*-Flag-SF/*lrbR*-Flag-SR, *lrbR*-Flag-SF/*lrbR*-Flag-OR, and *lrbR*-Flag-OF/*lrbR*-Flag-SR. A similar strategy was used to generate related 3×Flag-tagged strains. All transformants were verified by PCR and DNA sequencing.

To construct a C-terminal 1×HA-tagged LrbR transformant, the pK19-LrbR-CterUD (including the 3'-terminus region of the *lrbR* gene with its upstream and downstream flanks) was linearized by PCR amplification using the primers *lrbR*-HA-KinR and *lrbR*-HA-KinR (both were 5' phosphorylated primers containing the nucleotide sequence encoding HA). The linearized plasmid was self-ligated to yield the LrbR-HA knock-in plasmid, pK19-LrbR-Cter-HAKin, which was conjugated into related strains to obtain the transformant related LrbR-HA strains. The final transformants were verified by PCR, using the primers *lrbR*-Flag-SF/*lrbR*-Flag-SR, *lrbR*-Flag-SF/*lrbR*-Flag-OR, and *lrbR*-Flag-OF/*lrbR*-Flag-SR. A similar strategy was used to generate related 1×HA-tagged strains. All transformants were verified by PCR and DNA sequencing.

lrbA is co-transcribed with *lrbS*, and the RBS and start codon of *lrbS* are located in the ORF of *lrbA*. Therefore, knocking in a 3×Flag tag at the C-terminus of *lrbA* will affect the translation of *lrbS*. Oligonucleotides were synthesized (by Beijing Genomics Institute, China) according to the sequence in Supporting Information Table 4. The oligonucleotides encoding 3×Flag were inserted before the stop codon of *lrbA*. In addition, the oligonucleotides including a linker (a short oligonucleotide from *lrbA*), the RBS and the start codon of *lrbS* were added after the stop codon of *lrbA*. The synthesized oligonucleotides were digested by *Eco*RI/*Pst*I, and both were cloned into the *Eco*RI/*Pst*I-digested vector pK19*mobsacB* to yield pK19-*lrbA*-Flag, which was conjugated into related strains to obtain the transformant related LrbA-Flag strains. The final transformants were verified by PCR, using the primers *lrbA*-Flag-SF/*lrbA*-Flag-SR, *lrbA*-Flag-SF/*lrbA*-Flag-OR, and *lrbA*-Flag-OF/*lrbA*-Flag-SR. A similar strategy was used to generate related 3×Flag-tagged LrbA strains. All transformants were verified by PCR and DNA sequencing.

Biofilm microtiter plate assay

After using MM1 medium to dilute the LB seed broth to OD_{600}~0.01, 100 μL was aliquoted into 96-well cell culture plates (NEST, China). When necessary, 1 mM exogenous adenosine 3', 5'-cyclic monophosphate sodium salt monohydrate (cAMP) (Sigma-Aldrich, USA) was added into LB seed broth and MM1 medium. The 96-well cell culture plates were statically incubated at 30°C for different times. Static biofilm assay was based on a 96-well plate crystal violet dyeing method (1).

c-di-GMP measurement

The intracellular c-di-GMP concentration was measured using an established method (1). After using

MM1 medium to dilute the LB seed broth to OD_{600}~0.01, 100 μL was aliquoted into 96-well cell culture plates. *S. putrefaciens* CN32 cells grown in 96-well cell culture plates were harvested at 30 h by centrifugation at 13,000 g for 10 min at 4°C and washed with PBS buffer. Then, the cell samples were lysed by B-PER Bacterial Protein Extraction Reagent (ThermoFisher Scientific, USA), incubated at room temperature for 10 min, and then centrifuged at 13,000 g for 5 min. The liquid supernatant was used to measure the intracellular c-di-GMP concentration using a Cyclic di-GMP ELISA Kit (Cayman Chemical, USA) and determine the total protein concentration using a Quick Start Bradford 1×dye reagent (Bio-Rad, USA). The intracellular c-di-GMP concentrations were converted to picomoles per milligram of protein.

RNA extraction and real-time RT-PCR (qRT-PCR) assay

After using MM1 medium to dilute the LB seed broth to OD_{600}~0.01, 100 μL was aliquoted into 96-well cell culture plates. RNA was extracted from cells cultured in 96-well cell culture plates at the appropriate times using the TRIzol method. Following the manufacturer's protocol (Promega, USA), 2 μg of total RNA was reverse transcribed into cDNA, which was then used as a template for the qRT-PCR assay. The qRT-PCR assay was performed using the SYBR Green Master Mix (Biosharp Life Sciences, China) and analyzed using the Tianlong Gentier 96 Real-Time PCR System. The PCR program included a pre-denaturation step at 95°C for 10 min, 40 cycles of 95°C for 10 s and 60°C for 30 s; the fluorescence was measured at the end of each cycle. The primers used in qRT-PCR analysis were listed in Supporting Information Table 2, and the 16S rRNA gene was selected as an internal control. All of the experiments were performed at least three times.

Protein purification

To purify His_6-LrbR, pET28a-LrbR were constructed and transformed into *E. coli* BL21 (DE3) cells, which were cultured at 37°C in LB medium to an OD_{600}~0.6 and were induced with 0.4 mM isopropyl-β-D-thiogalactopyranoside (IPTG) for 24 h at 16°C, and the His_6-LrbR proteins were purified by Ni Sepharose 6FF (Solarbio Life Sciences, China) according to the manufacturer’s protocol. A similar strategy was used to purify His_6-LrbA, His_6-CRP, and His_6-CRP-R84L.

To purify GST-LrbR, pGEX-4T-1-LrbR was constructed and transformed into *E. coli* BL21 (DE3) cells, which were cultured at 37°C in LB medium to an OD_{600}~0.6. The cells were then inducted with 0.4 mM isopropyl-β-D-thiogalactopyranoside (IPTG) for 24 h at 16°C, and the GST-LrbR were purified by Glutathione-Sepharose resin (Solarbio Life Sciences, China) according to the manufacturer’s protocol. A similar strategy was used to purify GST-LrbA, GST-BpfD-In (intracellular domains of BpfD), and the GST protein.

Electrophoretic mobility shift assay (EMSA)

EMSA was performed as described previously (4). The DNA probes P_{intAR} covered intergenic region

between *lrbA* and *lrbR* from –403 bp to +34 bp relative to the *lrbR* start codon was amplified using the primers *lrbA*-*lrbR*-inter-EMSA-F/*lrbA*-*lrbR*-inter-EMSA-R. The site-mutated P_{intAR}-mut probe was synthesized (by Beijing Genomics Institute, China). The 3'-terminal ends of both purified probes were labeled with digoxigenin (DIG) using the DIG Gel Shift Kit, 2nd Generation (Roche, USA). EMSA was performed according to the manufacturer's protocol. The reaction mixture (20 μL) containing digoxigenin (DIG)-labeled DNA probes, the proteins, 1 μg of poly(dA-dT) (vial 10) and the binding buffer (vial 5) was incubated at 30°C for 30 min. When necessary, 1 μM cAMP (Sigma-Aldrich, USA) was added. The probes were separated by 5% native polyacrylamide gels and then transferred to a nylon membrane (Roche, USA). Cross-linking and chemiluminescence detection processes were performed as described in DIG Gel Shift Kit, 2nd Generation (Roche, USA).

Chromatin immunoprecipitation-qPCR (ChIP-qPCR)

ChIP-qPCR was performed as described previously (5). Anti-FLAG Affinity Gel (Bimake, USA) was balanced with the lysis buffer [HEPES 50 mM, NaCl 137 mM, EDTA 1 mM, Tris-HCl (pH 8.0) 10 mM] containing bovine serum albumin (BSA) 0.5 mg/mL and salmon sperm DNA 0.1 mg/mL. After using MM1 medium to dilute the LB seed broth to OD_{600}~0.01, 100 μL was aliquoted into 96-well cell culture plates. Strains grown in 96-well cell culture plates for 6 h were collected in an Erlenmeyer flask. *In vivo* cross-linking was performed by adding formaldehyde to the Erlenmeyer flask containing harvested strains to a final concentration of 1%, which was shaken at 30°C, 200 rpm for 30 min. Cross-linking was quenched by adding glycine to a final concentration of 0.125 M, shaking at 30°C, 200 rpm for 5 min. 0.15 g of cells were harvested by centrifugation at 13,000 g for 10 min at 4°C, washed three times with ice-cold PBS buffer and resuspended in 2 mL ice-cold lysis buffer [Protease inhibitor cocktail (CoWin Biosciences, China) was added before sonication]. The cellular DNA was sonicated to a mean size of 250 to 500 bp and centrifuged to remove cellular debris. The protein concentration of the supernatant was measured and adjusted to 2 mg/mL with lysis buffer. 10 μL of supernatant was retained as input sample. 1 mL of supernatant was added to 40 μL balanced Anti-FLAG Affinity Gel and rotated vertically at 4°C for 4 h. After incubation, the Affinity Gel (ChIP sample) was washed twice with ChIP buffer I (50 mM HEPES, 500 mM NaCl, 1 mM EDTA, 10 mM Tris-HCl, pH 8.0), three times with ChIP buffer II (250 mM LiCl, 1% NP-40, 1% sodium deoxycholate, 1 mM EDTA, 10 mM Tris-HCl, pH 8.0), and twice with ChIP buffer III (1 mM EDTA, 50 mM Tris-HCl, pH 8.0). The input sample and Affinity Gel (ChIP sample) were resuspended with 500 μL of fresh elution buffer (0.1 M $NaHCO_3$, 1% SDS, 250 mM NaCl, 0.1 mg/mL proteinase K) for elution and reverse cross-linking, both of which were incubated overnight at 65°C. After centrifugation at 13,000g for 10 min at 4°C, the nucleotide in the supernatant from the

input sample and Affinity Gel (ChIP sample) was precipitated with ethanol and resuspended in 200 μL ddH_2O and analyzed by qPCR.

cAMP measurement

The intracellular cAMP concentration was measured using an established method (1). After using MM1 medium to dilute the LB seed broth to OD_{600}~0.01, 100 μL was aliquoted into 96-well cell culture plates. *S. putrefaciens* CN32 cells grown in 96-well cell culture plates were harvested at appropriate time points by centrifugation at 12,000 g for 10 min at 4°C and washed twice with cold phosphate-buffered saline (PBS) buffer. Then, the cell samples were divided into two parts: one part was acetylated following the manufacturer's protocol and used to measure the intracellular cAMP concentration with a Cyclic AMP ELSA Kit (Cayman Chemical, USA), and the other was used to determine the total protein concentration using a Quick Start Bradford 1×dye reagent (Bio-Rad, USA). The intracellular cAMP concentrations were converted to picomoles per milligram of protein.

GST pull-down experiment

The GST pull-down experiment was performed using an established method(1). PBS-glycerol buffer (PBS buffer containing 20% of glycerol) was used to dialyze His_6-CRP, His_6-CRP-R84L, His_6-LrbR, GST-LrbR, GST-LrbA, GST-BpfD-In, and GST protein. GST-tagged and His_6-tagged proteins were combined in equal amounts and 20 μM cAMP was added as necessary. The three proteins (GST-BpfD-In, His_6-CRP, and His_6-LrbR, or GST-BpfD-In, His_6-CRP-R84L, and His_6-LrbR) were also mixed in equal amounts to determine the three-protein interaction. Pull-down buffer (50-mM Na_2HPO_4/NaH_2PO_4, pH 7.8, 200 mM NaCl, 1 mM EDTA, 0.5% Nonidet P-40) was added to a final volume of 1 mL. To each volume, 50 μL of pre-balanced Glutathione-Sepharose resin (Solarbio Life Sciences, China) was added and incubated for 2 h at 4°C with generous rotation. Then, the Glutathione-Sepharose resin was washed five times for 10 min each with pull-down buffer, and 20 μM cAMP was supplied if necessary. The bound proteins were analyzed by Western blotting.

Phos-tag PAGE

The phosphorylation status of LrbA was measured using an established method with minor modifications (6). Specifically, after using MM1 medium to dilute the LB seed broth to OD_{600}~0.01, 100 μL was aliquoted into 96-well cell culture plates. Strains grown in 96-well cell culture plates for appropriate time points were harvested by centrifugation at 13, 000 g for 8 min at 4°C, and the pellets were snap frozen in liquid nitrogen and stored at −80°C. Cells were lysed in 400 μL lysis buffer [5 mL B-PER Bacterial Protein Extraction Reagent (ThermoFisher Scientific, USA) containing 1 PhosSTOP phosphatase inhibitor tablet (Roche, USA), 1 cOmplete EDTA-free protease inhibitor cocktail tablet (Roche, USA), 1 mg lysozyme (ThermoFisher Scientific, USA) and 50 U DNase I (ThermoFisher Scientific, USA)] for 5 min at room temperature. Cell lysates were

centrifugated at 13,000 g for 10 min at 4°C, and 200 μL supernatants were mixed with 100 μL 3×loading buffer (0.2 mol/L Tris-Cl, pH 6.8, 6% [w/v] SDS, 15% [v/v] β-mercaptoethanol, 30% [v/v] glycerol, 0.015%[w/v] bromophenol blue), boiled for 5 min and kept on ice as samples for SDS-PAGE. The rest of supernatants were used to measure total protein concentration with Quick Start Bradford 1×dye reagent (Bio-Rad, USA). Subsequently, boiled samples containing 1 μg total protein were run on 15% SDS-PAGE gels supplemented with 150 μM Phos-tag acrylamide compound (Wako, Japan) and 300 μM $MnCl_2$ for 3 h at 35 mA in ice bath. The following transmembrane assay and immunoblotting were performed as described in Western blotting section.

Co-immunoprecipitation (Co-IP) assay

The Co-IP assay was performed using an established method (1). Co-IP was performed to verify the interaction between Flag-tagged LrbA and HA-tagged CRP, Flag-tagged BpfD and HA-tagged BpfG, Flag-tagged LrbR and HA-tagged CRP, Flag-tagged BpfD and HA-tagged CRP, and Flag-tagged BpfD and HA-tagged LrbR. After using MM1 medium to dilute the LB seed broth to OD_{600}~0.01, 100 μL was aliquoted into 96-well cell culture plates. Strains grown in 96-well cell culture plates for appropriate time points were harvested, washed once with PBS buffer and resuspended in ice-cold lysis buffer (45-mM HEPES, pH 7.2, 10% glycerol, 0.2% NP-40, 150 mM NaCl, 1 mM EDTA, 2 mM DTT, and 1×protease inhibitor cocktail [CoWin Biosciences, China]) for 30 min. The cell lysates were centrifuged at 13, 000 g for 20 min at 4°C. A Quick Start Bradford 1×dye reagent (Bio-Rad, USA) was used to determine the concentration of total protein in the supernatant, which was adjusted to 2 mg/mL of protein. The supernatant was incubated with anti-Flag M2 antibody [DYKDDDDK-Tag (3B9) mAb (Abmart, China)] and Recombinant Protein G-Sepharose 4B (Invitrogen, USA) on a rotary shaker at 4°C for 2 h. The Mouse IgG (Abmart, China) was used as negative control. The protein-bead complexes were then washed three times with lysis buffer. When necessary, 5 μM cAMP was added to the lysis buffer to perform interaction analysis between Flag-tagged LrbA or Flag-tagged LrbR and HA-tagged CRP, and 5 μM c-di-GMP was added to the lysis buffer to perform interaction analysis between Flag-tagged BpfD and HA-tagged BpfG. Sample-loading-buffer was used to elute the bound protein complexes from the beads, and Western blotting was used for analysis.

BpfA localization assay

The harvesting of cells and the collection of BpfA on cell surface were determined using an established method (1). After using MM1 medium to dilute the LB seed broth to OD_{600}~0.01, 100 μL was aliquoted into 96-well cell culture plates. Cells were adjusted to the same cell concentration and harvested and from different strains grown in 96-well cell culture plates for appropriate time points. Specifically, 30 mL of adjusted cells was harvested by centrifugation at 12,000 g for 10 min at 4°C,

resuspended in 150 μL PBS buffer, and mixed with 150 μL PBS buffer containing 8 mg/mL lysozyme (Solarbio Life Sciences, China). After 15 minutes of incubation at 37°C, the cell suspensions were centrifuged at 13,000 g for 5 min at 4°C. Finally, the BpfA-containing supernatant fraction was determined by Western blotting.

Extraction of intracellular total proteins

After using MM1 medium to dilute the LB seed broth to OD_{600}~0.01, 100 μL was aliquoted into 96-well cell culture plates. To analyze the intracellular protein levels of LrbR, LrbA, LrbS, CRP, BpfA, BpfD, BpfG, Sputcn32_1291, Sputcn32_3328, and Sputcn32_1235, cells from different strains grown in 96-well cell culture plates for appropriate time points were harvested and treated B-PER Bacterial Protein Extraction Reagent (ThermoFisher Scientific, USA). Total protein concentration was measured using a Quick Start Bradford 1×dye reagent (Bio-Rad, USA). Total proteins from different strains were adjusted to equal amounts for Western blotting assay.

Western blotting

The intracellular LrbA proteins were separated by 15% SDS-PAGE gels to be consistent with Phos-tag PAGE. Protein samples except LrbA and BpfA were separated by 12% SDS-PAGE gels. PageRuler^{TM} pre-stained protein ladder (ThermoFisher Scientific, USA) and Multicolor prestained protein ladder (Epizyme Biotech, China) was used to identify the protein molecular weight (except BpfA). The total BpfA proteins and BpfA on cell surface were separated by 5% SDS-PAGE gels and HiMark^{TM} pre-stained protein standard (ThermoFisher Scientific, USA) was used to identify the BpfA molecular weight. Then, they were transferred onto a PVDF membrane (Roche, USA). After blocking with skim milk (5% in TBST), the membranes were incubated with primary antibodies against the target protein, followed by incubation with HRP-conjugated secondary antibodies. Alternatively, after blocking with skim milk (5% in TBST), the membranes were incubated with Anti-HA-Tag Antibody (HRP Conjugated) (Abmart, China). The target protein was then detected using the eECL Western Blotting Kit (CoWin Biosciences, China) or SuperFemto ECL Chemiluminescence Kit (Vazyme, China) according to the manufacturer's protocol. Primary antibody: Monoclonal anti-Flag M2 antibody produced in mouse (Sigma-Aldrich, USA); anti-Flag antibody [DYKDDDDK-Tag (3B9) mAb] (Abmart, China); HA-Tag (26D11) mAb (Abmart, China); Anti HA-Tag mouse monoclonal antibody (CoWin Biosciences, China); Anti GST-Tag mouse monoclonal antibody (CoWin Biosciences, China); Anti His-Tag mouse monoclonal antibody (CoWin Biosciences, China). HRP-conjugated secondary antibody: Goat anti-mouse IgG HRP conjugated secondary antibody (CoWin Biosciences, China); Goat Anti-Mouse IgG-Fc HRP conjugated secondary antibody (SinoBiological, China); Goat Anti-Mouse IgG HRP (light chain specific) (Abmart, China). Antibodies for different samples are annotated in the Source Data.

Supporting Information Figures

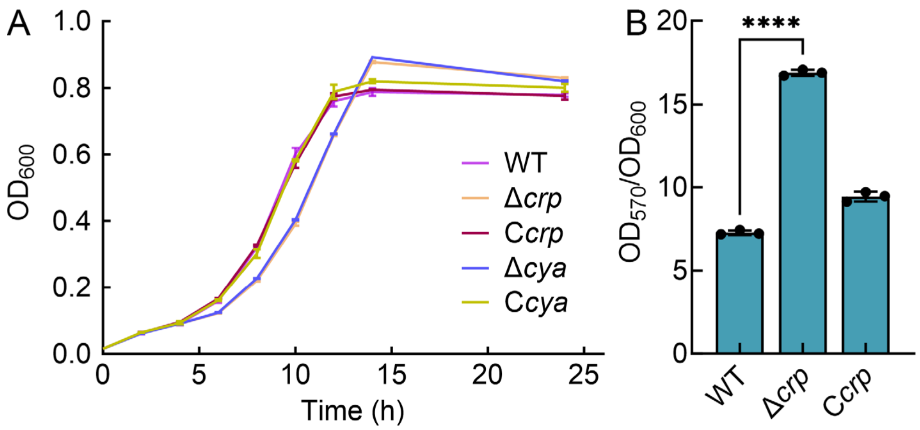

Fig. S1. (A) Planktonic cell growth of related strains in MM1 medium (n=3 independent samples). (B) The ratio of biofilm biomass to cell growth (OD_{570}/OD_{600}) at 6 h in MM1 medium (n=3 independent samples). Data are shown as the mean ± SD. One-way ANOVA followed by Tukey's multiple comparison tests was used in (B) to analyze the statistical significance, which was provided by GraphPad Prism 10 statistical software (****$p < 0.0001$).

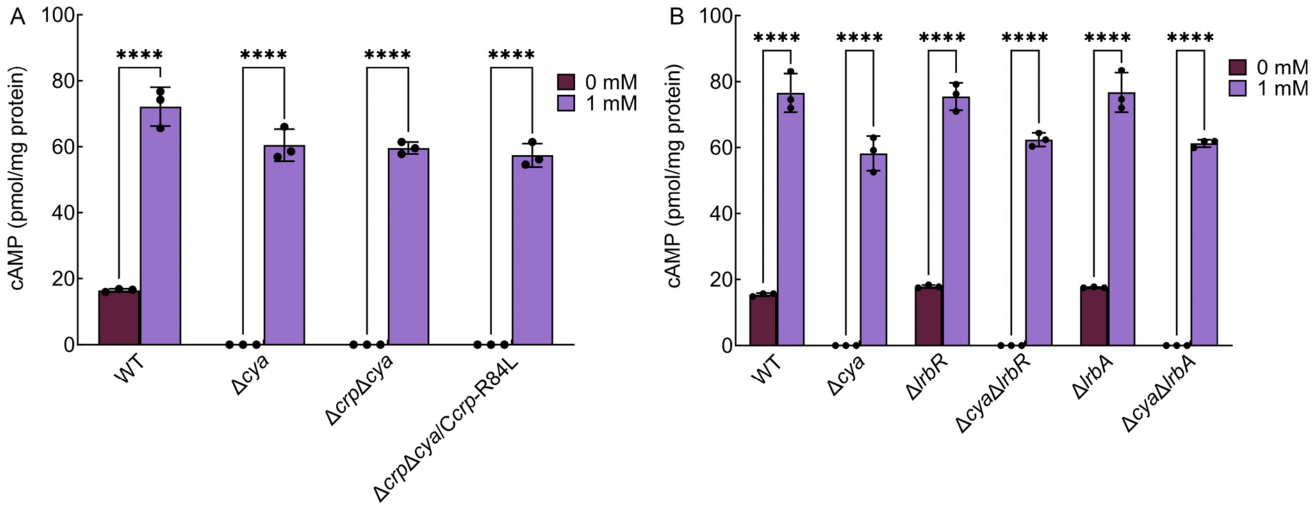

Fig. S2. Intracellular cAMP concentration with the addition of 1 mM exogenous cAMP to the culture medium vs. control (no addition of exogenous cAMP [0 mM]) at 6 h in MM1 medium (n=3 independent samples). Data are shown as the mean ± SD. Two-way ANOVA followed by Tukey's multiple comparison tests was used to analyze the statistical significance, which was provided by GraphPad Prism 10 statistical software (****p < 0.0001).

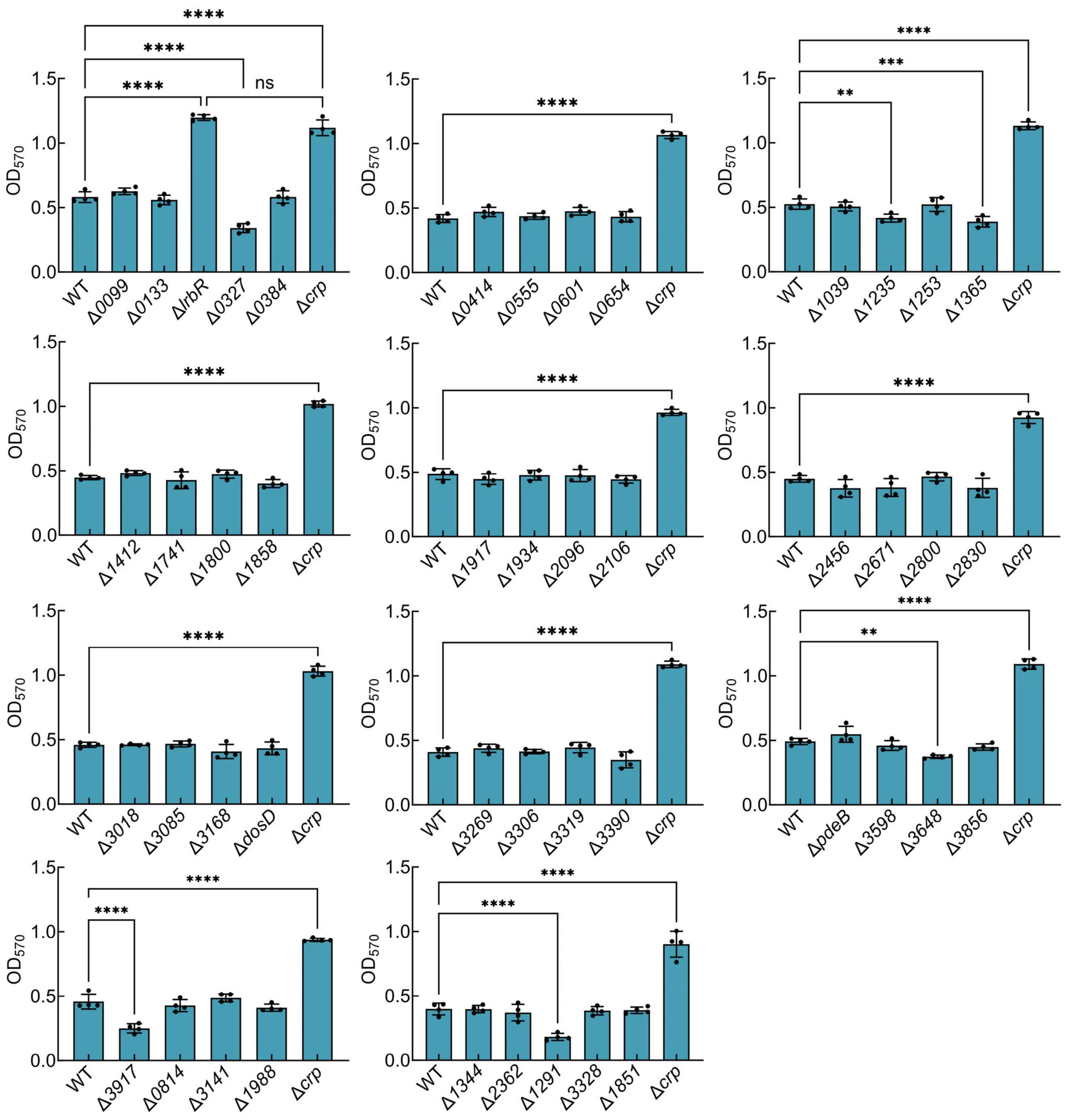

Fig. S3. Biofilm biomass of mutants with deletion of 46 *dgc/pde* genes at 6 h in MM1 medium (n=4 independent samples). Data are shown as the mean ± SD. One-way ANOVA followed by Tukey's multiple comparison tests was used to analyze the statistical significance, which was provided by GraphPad Prism 10 statistical software (ns: no significance, ***p* < 0.01, ****p* < 0.001, *****p* < 0.0001).

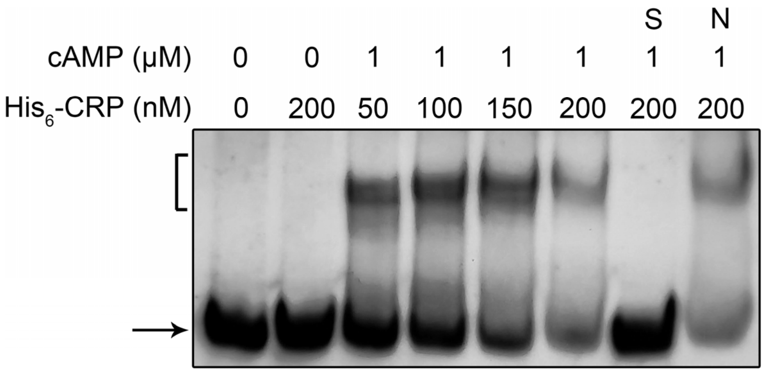

Fig. S4. EMSA of cAMP-CRP binding to probe P_{intAR} [the intergenic region between *lrbR* and its upstream gene (*lrbA*)]. The concentrations of His_6-CRP and cAMP are shown above the figure. The black arrow indicated the free DNA probe P_{intAR}. The binding specificity was confirmed by competitive assays with a 300-fold excess of unlabeled specific probe P_{intAR} (lane S) or unlabeled nonspecific competitor DNA (probe *recA*) (lane N).

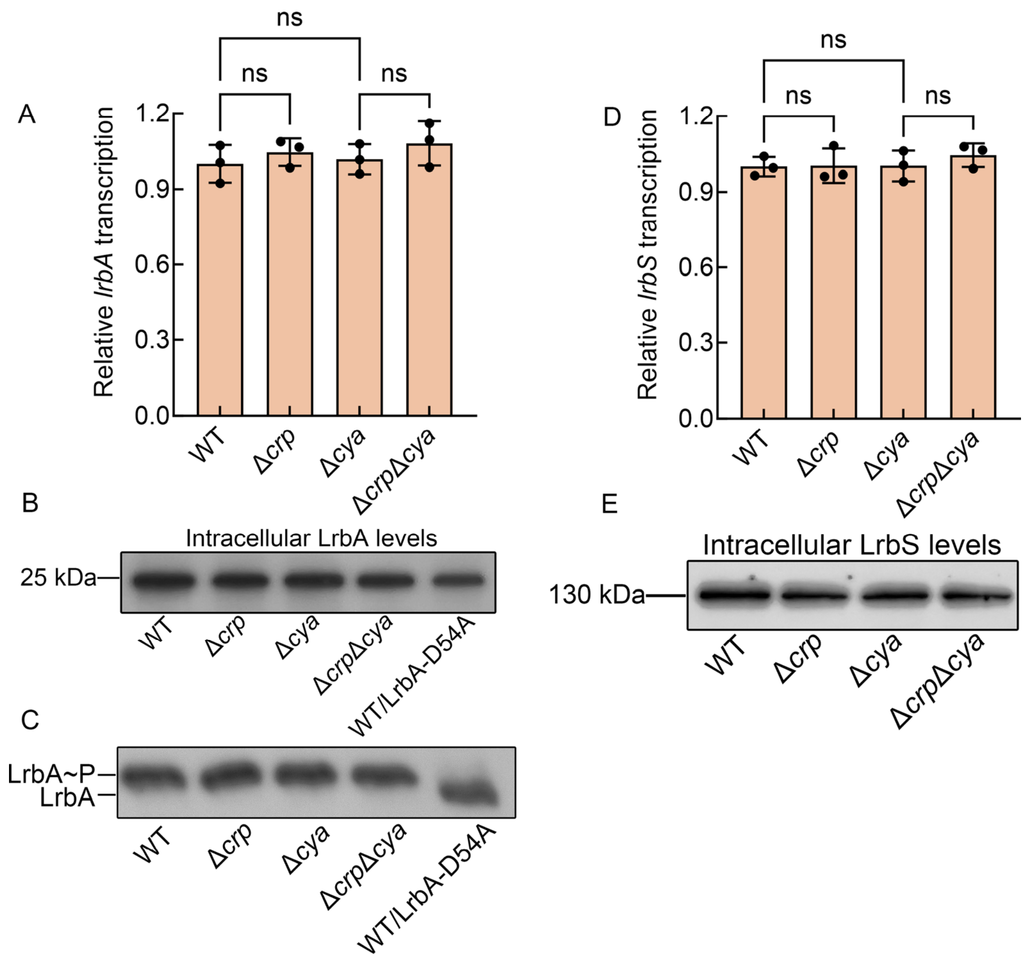

Fig. S5. The expression of the *lrbA*-*lrbS* operon and the phosphorylation status of LrbA were not affected by cAMP-CRP. (A) Transcriptional analysis of *lrbA* at 6 h (n=3 independent samples). The relative transcription value of the evaluated gene in the WT strain was set to 1. (B) Western blotting detection of intracellular LrbA levels at 6 h. WT/LrbA-D54A as the control. (C) Phos-tag PAGE analysis the phosphorylation status of LrbA at 6 h. WT/LrbA-D54A is the dephosphorylation control. (D) Transcriptional analysis of *lrbS* at 6 h (n=3 independent samples). The relative transcription value of the evaluated gene in the WT strain was set to 1. (E) Western blotting detection of intracellular LrbS levels at 6 h. All strains used in (A-E) were cultured in MM1 medium. Data in (A and D) are shown as the mean ± SD. One-way ANOVA followed by Tukey's multiple comparison tests was used in (A and D) to analyze the statistical significance, which was provided by GraphPad Prism 10 statistical software (ns: no significance).

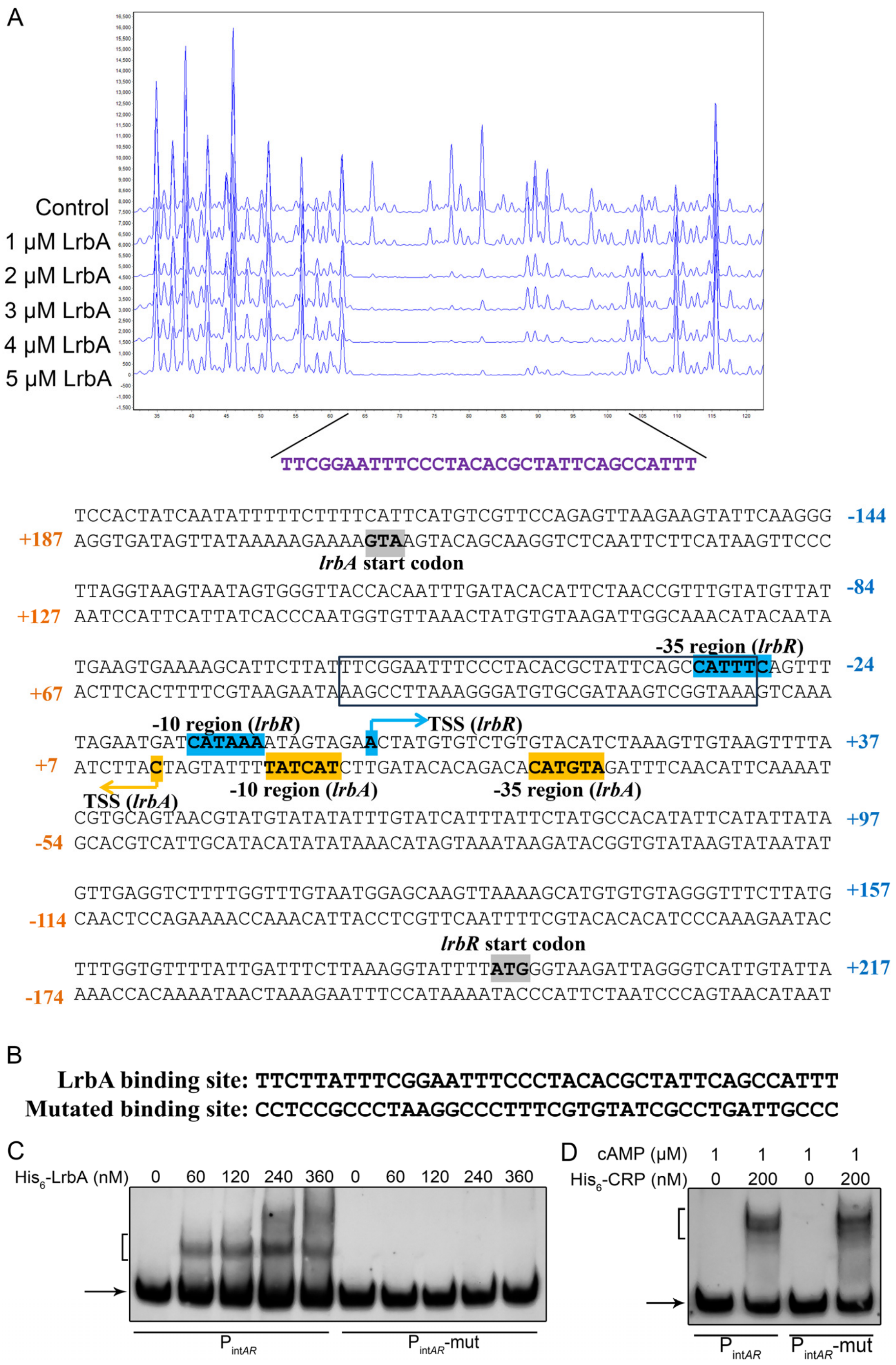

Fig. S6. (A) DNase I footprinting assay of the LrbA and *lrbA-lrbR* intergenic region. Each reaction mixture contains 300 ng of FAM-labeled *lrbA-lrbR* intergenic region probe, and the LrbA concentration is shown on the left of the figure. Control: no addition of LrbA protein. The nucleotide sequences of the protected region are shown in bold purple below the figure. The black nucleotide sequences are the intergenic region between *lrbA* and *lrbR*. The nucleotide sequences in a box in the *lrbA-lrbR* intergenic region are also the LrbA protected region. The transcription start site (TSS, +1) and the predicted -10 region and -35 region of *lrbA* are shown by the yellow arrow and bold. The TSS and the predicted -10 region and -35 region of *lrbR* are shown by the blue arrow and bold. The

numbers located on the left side of the *lrbA-lrbR* intergenic region indicate the distance (nt) from the TSS of *lrbA* (4), and the numbers located on the right side of the *lrbA-lrbR* intergenic region indicate the distance (nt) from the TSS of *lrbR* (4). The start codon of both genes is shown in gray bold. (B) The binding sequence of LrbA was mutated according to the principle that G intermutates with A (G←→A) and T intermutates with C (T←→C). (C) EMSA of LrbA binding to probe $P_{int\mathit{AR}}$ (the intergenic region between *lrbR* and *lrbA*) and probe $P_{int\mathit{AR}}$-mut ($P_{int\mathit{AR}}$ probe containing the mutated sequence in Fig. S6B). The concentrations of LrbA are shown above the figure. The black arrow indicated the free DNA probe $P_{int\mathit{AR}}$ or $P_{int\mathit{AR}}$-mut. (D) EMSA of cAMP-CRP binding to probe $P_{int\mathit{AR}}$ and probe $P_{int\mathit{AR}}$-mut. The concentrations of His_6-CRP and cAMP are shown above the figure. The black arrow indicated the free DNA probe $P_{int\mathit{AR}}$ or $P_{int\mathit{AR}}$-mut.

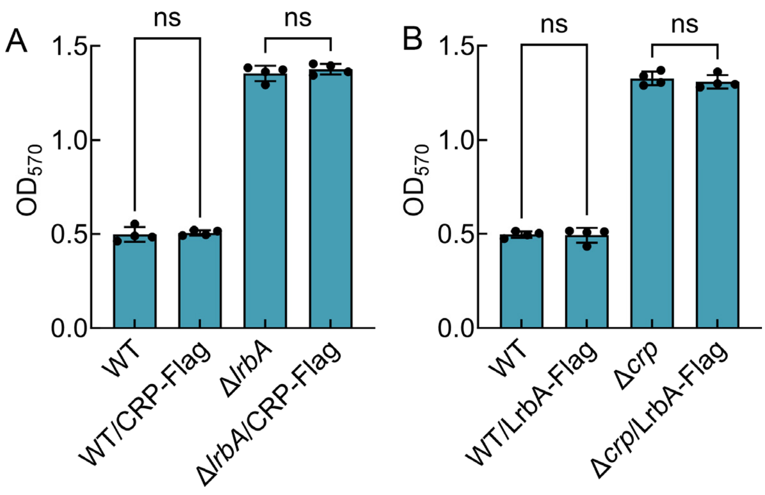

Fig. S7. Biofilm biomass of related strains at 6 h in MM1 medium (n=4 independent samples). Data are shown as the mean ± SD. One-way ANOVA followed by Tukey's multiple comparison tests was used in to analyze the statistical significance, which was provided by GraphPad Prism 10 statistical software (ns: no significance).

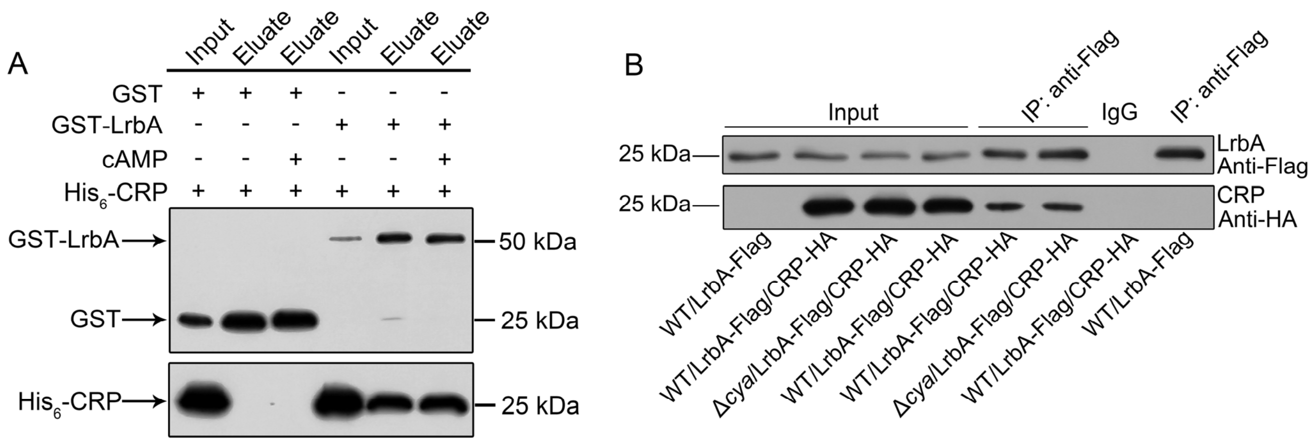

Fig. S8. (A) GST pull-down assay showing the interaction between LrbA and CRP *in vitro*. The addition of cAMP was 20 μM. (B) Co-IP to analyze the interaction between LrbA and CRP *in vivo* at 6 h in MM1 medium. 5 μM cAMP was added only to cell lysis of all WT samples. WT/LrbA-Flag was used as a negative control. Co-IP using antibodies against LrbA, CRP, and immunoglobulin G (IgG) negative control.

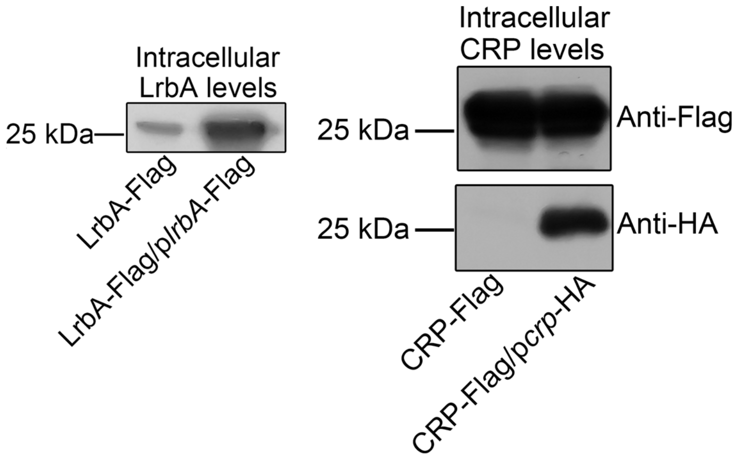

Fig. S9. Western blotting detection of intracellular LrbA and CRP levels at 6 h in MM1 medium.

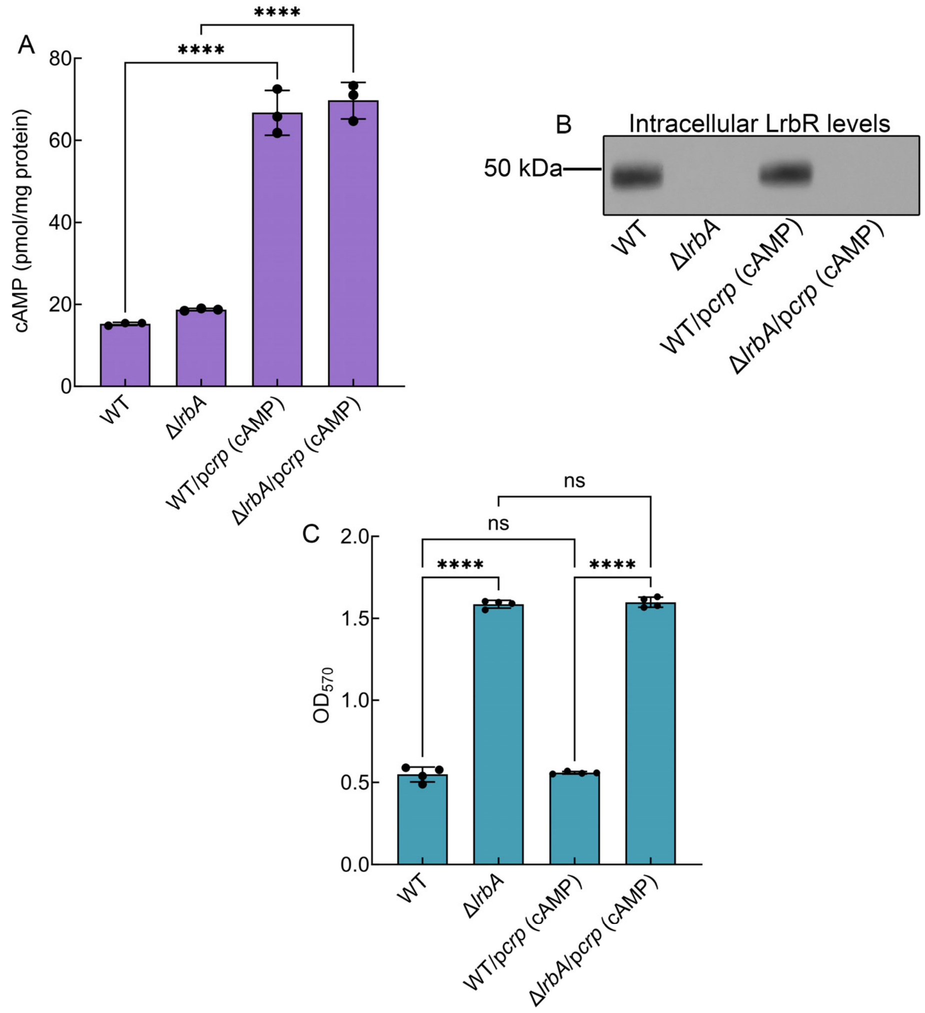

Fig. S10. (A) Intracellular cAMP concentration at 6 h (n=3 independent samples). (B) Western blotting detection of intracellular LrbR levels at 6 h. (C) Biofilm biomass at 6 h (n=4 independent samples). The cAMP in brackets indicated the addition of 1 mM exogenous cAMP to the culture medium. All strains used in (A-C) were cultured in MM1 medium. Data in (A and C) are shown as the mean ± SD. One-way ANOVA followed by Tukey's multiple comparison tests was used in (A and C) to analyze the statistical significance, which was provided by GraphPad Prism 10 statistical software (ns: no significance, ****$p < 0.0001$).

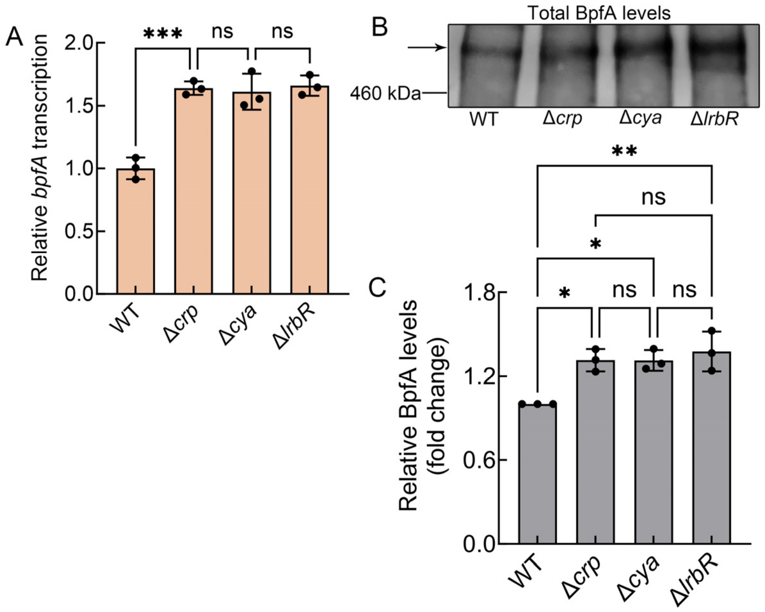

Fig. S11. (A) Transcriptional analysis of *bpfA* at 6 h (n=3 independent samples). The relative transcription value of the evaluated gene in the WT strain was set to 1. (B-C): Western blotting detection of total BpfA levels at 6 h (B), and band intensities were quantified using Image J soft-ware and normalized to WT (n=3 independent samples) (C). All strains used in (A-C) were cultured in MM1 medium. One-way ANOVA followed by Tukey's multiple comparison tests was used in (A and C) to analyze the statistical significance, which was provided by GraphPad Prism 10 statistical software (ns: no significance, *$p < 0.05$, **$p < 0.01$, ***$p < 0.001$).

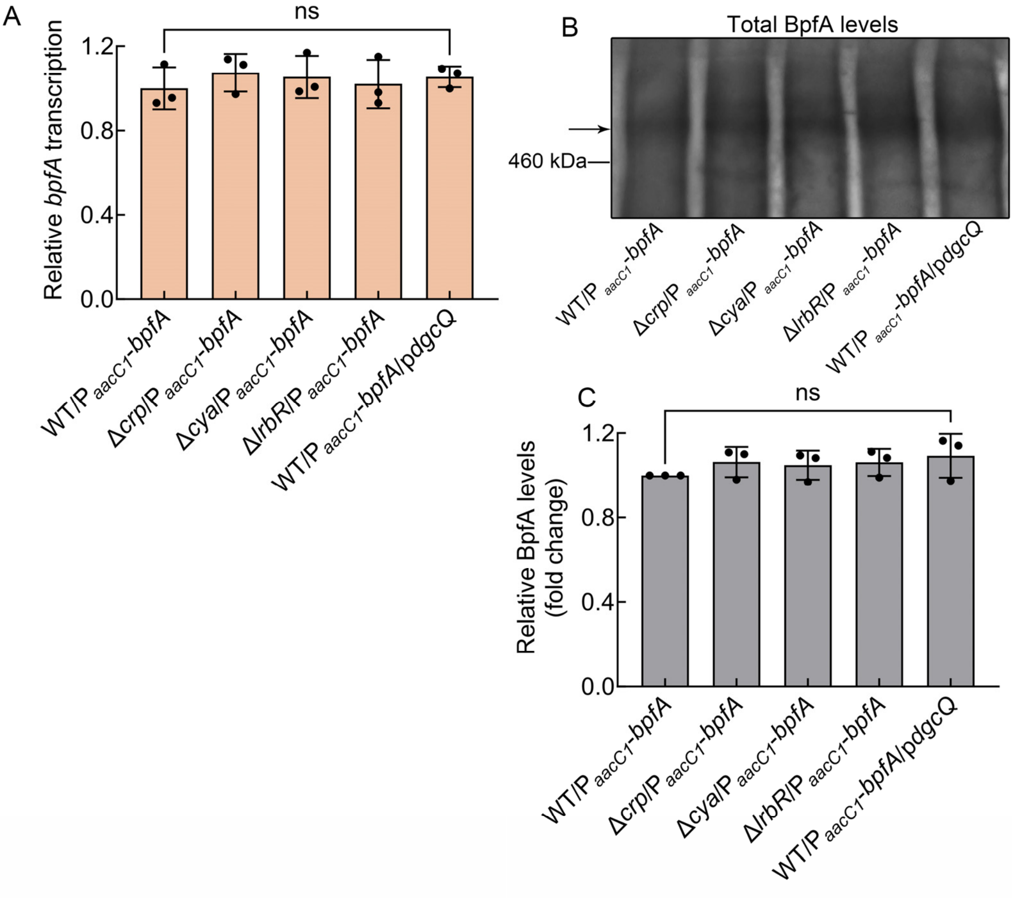

Fig. S12. Replacement of the promoter experiments. (A) Transcriptional analysis of *bpfA* at 6 h (n=3 independent samples). The relative transcription value of the evaluated gene in the WT strain was set to 1. (B-C): Western blotting analysis of total BpfA levels at 6 h (B), and band intensities were quantified using Image J soft-ware and normalized to WT (n=3 independent samples) (C). P_{aacC1}-*bpfA* indicates that the *bpfA* operon driven by the *aacC1* promoter. All strains used in (A-C) were cultured in MM1 medium. Data in (A and C) are shown as the mean ± SD. One-way ANOVA followed by Tukey's multiple comparison tests was used in (A and C) to analyze the statistical significance, which was provided by GraphPad Prism 10 statistical software (ns: no significance).

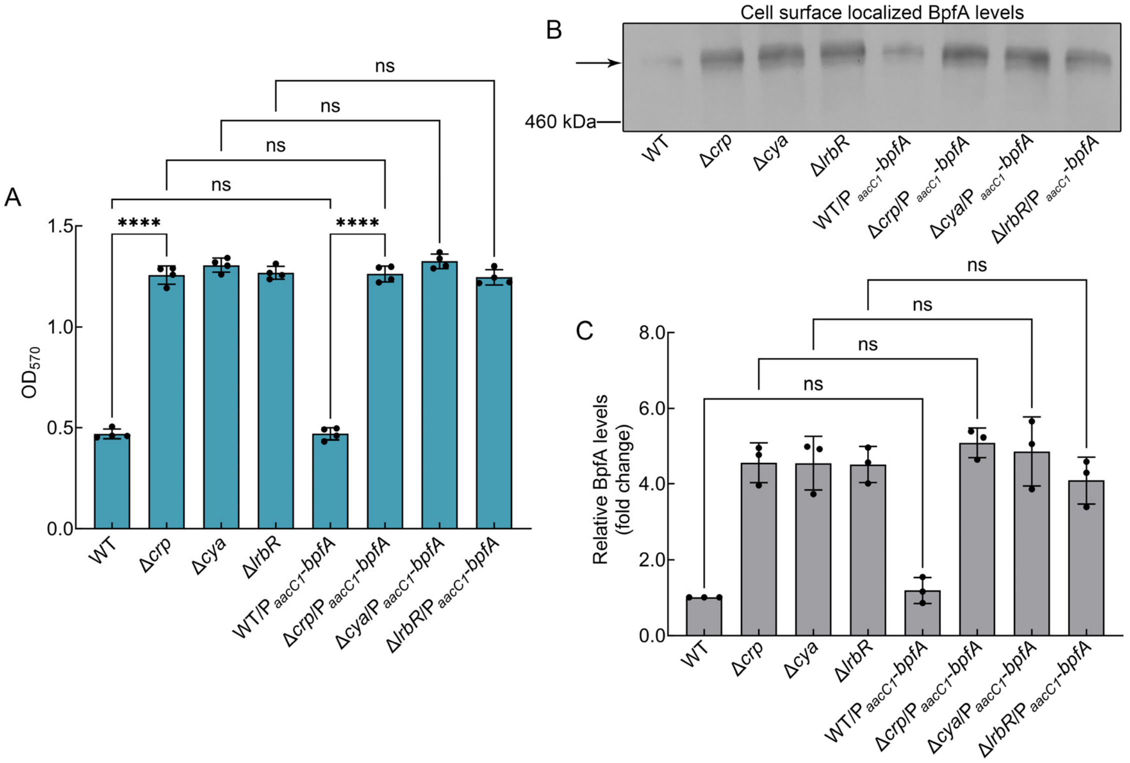

Fig. S13. (A) Biofilm biomass at 6 h (n=4 independent samples). (B-C): Western blotting detection of cell surface localized BpfA levels at 6 h (B), and band intensities were quantified using Image J soft-ware and normalized to WT (n=3 independent samples) (C). P_{aacC1}-*bpfA* indicates that the *bpfA* operon driven by the *aacC1* promoter. All strains used in (A-C) were cultured in MM1 medium. One-way ANOVA followed by Tukey's multiple comparison tests was used in (A and C) to analyze the statistical significance, which was provided by GraphPad Prism 10 statistical software (ns: no significance, ****$p < 0.0001$).

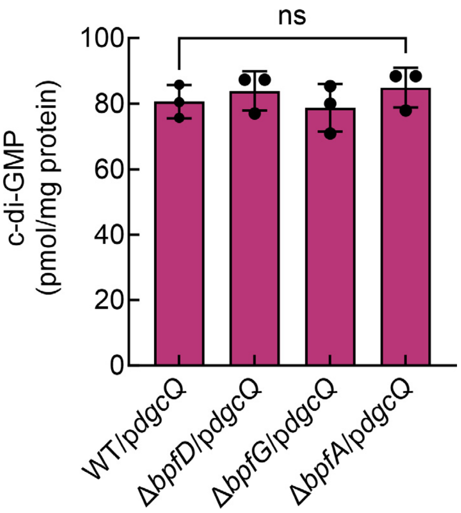

Fig. S14. Intracellular c-di-GMP concentration at 6 h in MM1 medium (n=3 independent samples). Data are shown as the mean ± SD. One-way ANOVA followed by Tukey's multiple comparison tests was used to analyze the statistical significance, which was provided by GraphPad Prism 10 statistical software (ns: no significance).

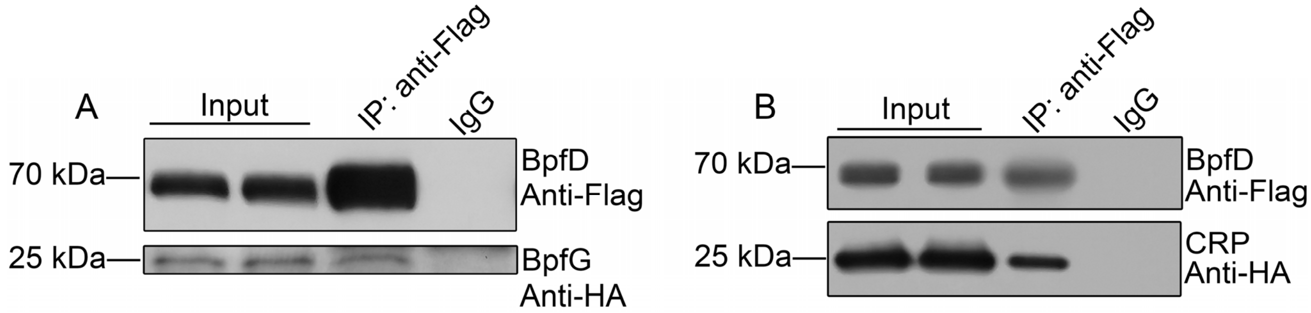

Fig. S15. Control of Co-IP assays in WT. (A) Co-IP to analyze the interaction between BpfD and BpfG *in vivo* at 6 h in MM1 medium. Co-IP using antibodies against BpfD, BpfG, and immunoglobulin G (IgG) negative control. (B) Co-IP to analyze the interaction between BpfD and CRP *in vivo* at 6 h in MM1 medium. Co-IP using antibodies against BpfD, CRP, and immunoglobulin G (IgG) negative control.

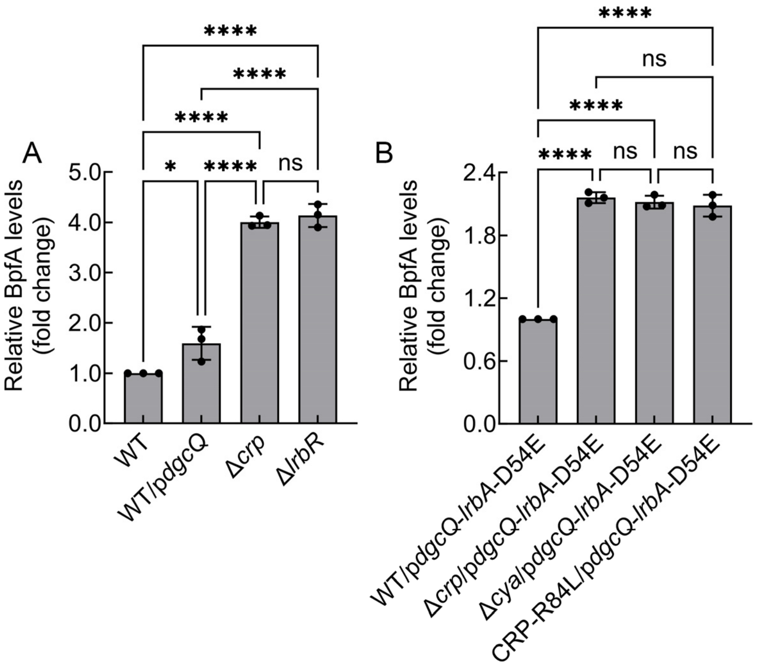

Fig. S16. Band intensities of BpfA related western blotting were quantified using Image J soft-ware and normalized to WT. (A) Band intensities of cell surface localized BpfA levels at 6 h in Fig. 3G (n=3 independent samples). (B) Band intensities of cell surface localized BpfA levels at 6 h in Fig. 4I (n=3 independent samples). Data are shown as the mean ± SD. One-way ANOVA followed by Tukey's multiple comparison tests was used to analyze the statistical significance, which was provided by GraphPad Prism 10 statistical software (ns: no significance, **p* <0.05, *****p* < 0.0001).

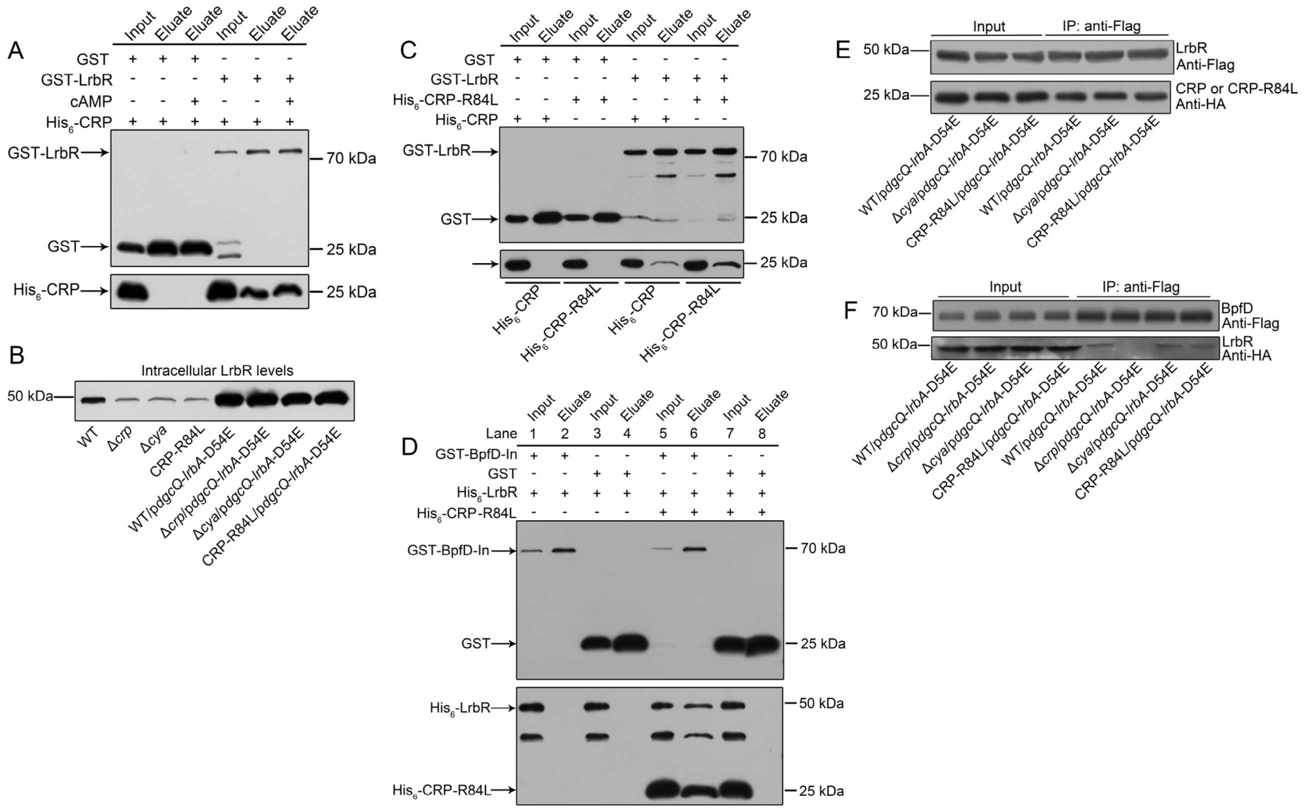

Fig. S17. (A) GST pull-down assay showing the interaction between LrbR and CRP *in vitro*. The addition of cAMP was 20 μM. (B) Western blotting detection of intracellular LrbR levels at 6 h. (C) GST pull-down assay showing the interaction between LrbR and CRP-R84L *in vitro*. (D) GST pull-down assay showing the BpfD-LrbR interaction and BpfD-(CRP-R84L)-LrbR interaction *in vitro*. (E) Co-IP to analyze the interaction between LrbR and CRP *in vivo* at 6 h. 5 μM cAMP was added only to cell lysis of the WT sample. (F) Co-IP to analyze the interaction between BpfD and LrbR *in vivo* at 6 h. 5 μM cAMP was added only to cell lysis of the WT sample. The strains used in (F) contain the *bpfA* operon driven by the *aacC1* promoter to exclude the influence of *bpfA*, *bpfD*, *bpfG* transcription caused by the introduction of *dgcQ* or the overexpression of LrbA-D54E or the deletion of *cya* and *crp* or the replacement of CRP-R84L. All strains used in (B, E, F) were cultured in MM1 medium.

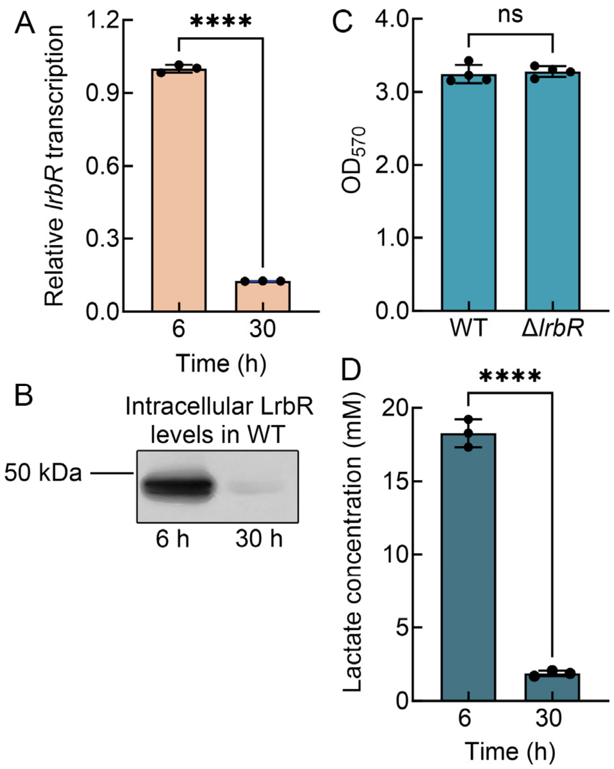

Fig. S18. (A) Transcriptional analysis of *lrbR* in WT at 6 h and 30 h (n=3 independent samples). The relative transcription value of the evaluated gene at 6 h was set to 1. (B) Western blotting detection of intracellular LrbR levels in WT at 6 h and 30 h. (C) Biofilm biomass at 30 h (n=4 independent samples). (D) Residual lactate concentration in MM1 medium at 6 h and 30 h (n=3 independent samples). All strains used in (A-C) were cultured in MM1 medium. Data in (A, C, D) are shown as the mean ± SD. Two-sided Student's *t*-test was used in (A, C, D) to analyze the statistical significance, which was provided by GraphPad Prism 10 statistical software (ns: no significance, ****$p < 0.0001$).

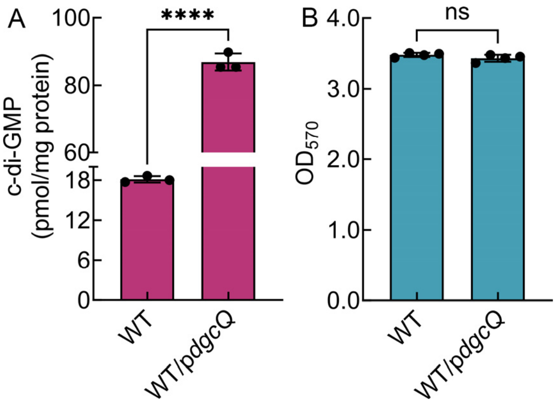

Fig. S19. Related phenotypes of WT and WT/p*dgcQ* in MM1 at 30 h. (A) Intracellular c-di-GMP concentration at 30 h (n=3 independent samples). (B) Biofilm biomass at 30 h (n=4 independent samples). All strains used in (A and B) were cultured in MM1 medium. Data are shown as the mean ± SD. Two-sided Student's *t*-test was used to analyze the statistical significance, which was provided by GraphPad Prism 10 statistical software (ns: no significance, ****$p < 0.0001$).

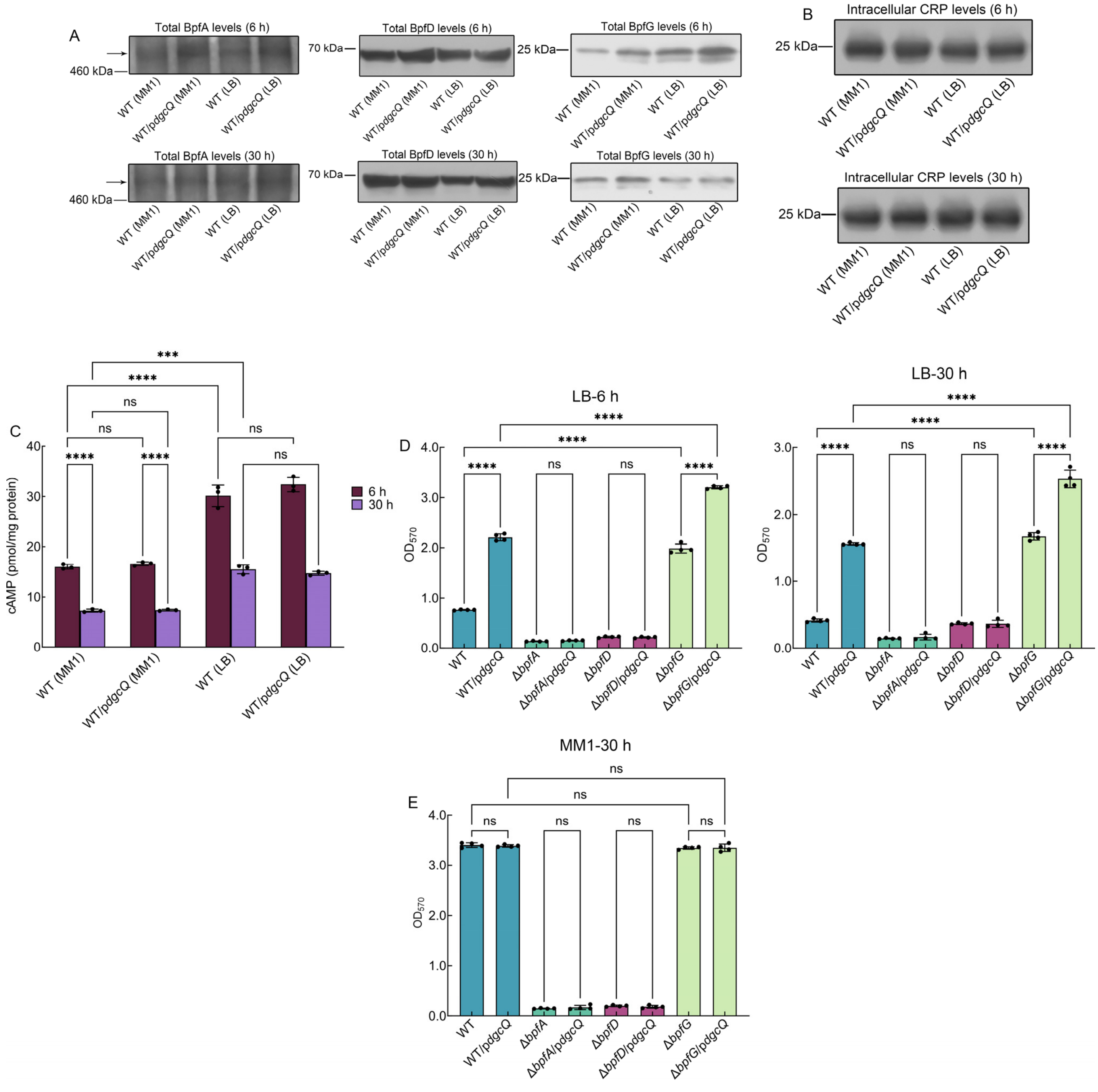

Fig. S20. A comparison of related phenotypes in different media. (A) Western blotting detection of total BpfA, BpfD, and BpfG levels at 6 h and 30 h in MM1 medium or LB medium. (B) Western blotting detection of intracellular CRP levels at 6 h and 30 h in MM1 medium or LB medium. (C) Intracellular cAMP concentration of WT or WT/p*dgcQ* in MM1 medium or LB medium (n=3 independent samples). (D) Biofilm biomass at 6 h and 30 h in LB medium (n=4 independent samples). (E) Biofilm biomass at 30 h in MM1 medium (n=4 independent samples). Data in (C-E) are shown as the mean ± SD. Two-way ANOVA (C) and one-way ANOVA (D and E) followed by Tukey's multiple comparison tests was used to analyze the statistical significance, which was provided by GraphPad Prism 10 statistical software (ns: no significance, ***$p < 0.001$, ****$p < 0.0001$).

Supporting Information Tables

Table S1. Strains and plasmids used in this study

Strains or Plasmids	Usages or Descriptions	Sources or references
Escherichia coli		
DH5α	Routine cloning host. *fhuA2*, *lacΔU169*, *phoA*, *glnV44*, *Φ80'*, *lacZΔM15*, *gyrA96*, *recA1*, *relA1*, *endA1*, *thi-1*, *hsdR17*	Lab stock
DH5α/p*crp*	DH5α carrying pBBR1MCS-2-P_{aacC1}-*crp*; Km^r	(1)
DH5α/p*cya*	DH5α carrying pBBR1MCS-2-P_{aacC1}-*cyaA-cyaB-cyaC*; Km^r	(1)
DH5α/p*crp*-R84L	DH5α carrying pBBR1MCS-2-P_{aacC1}-*crp*-R84L; Km^r	(1)
DH5α/p*lrbA*	DH5α carrying pBBR1MCS-2-P_{aacC1}-*lrbA*; Km^r	(4)
DH5α/p*lrbA*-D54E	DH5α carrying pBBR1MCS-2-P_{aacC1}-*lrbA*-D54E; Km^r	(4)
DH5α/p*lrbA*-D54A	DH5α carrying pBBR1MCS-2-P_{aacC1}-*lrbA*-D54A; Km^r	(4)
DH5α/p*dgcQ*	DH5α carrying pBBR1MCS-2-P_{aacC1}-*dgcQ*$_{MG1655}$; Km^r	(1)
DH5α/p*dgcQ-lrbA*-D54E	DH5α carrying pBBR1MCS-2-P_{aacC1}-*dgcQ*$_{MG1655}$-*lrbA*-D54E; Km^r	This study
DH5α/p*1235*	DH5α carrying pBBR1MCS-2-P_{aacC1}-*Sputcn32_1235*; Km^r	This study
DH5α/p*1235*-GGAAF	DH5α carrying pBBR1MCS-2-P_{aacC1}-*Sputcn32_1235*-(GGDEF→GGAAF); Km^r	This study
DH5α/pDGC2	DH5α carrying pBBR1MCS-2-P_{aacC1}-*Sputcn32_1291-Sputcn32_3328*; Km^r	(1)
DH5α/pK19-D*crp*	DH5α carrying *crp*-deletion plasmid, pK19*mobsacB*-D*crp*; Km^r	(1)
DH5α/pK19-*crp*-R84L-knock-in	DH5α carrying CRP-R84L knock-in plasmid, pK19*mobsacB*-*crp*-R84L-knock-in; Km^r	This study
DH5α/pK19-D*lrbA*	DH5α carrying *lrbA*-deletion plasmid, pK19*mobsacB*-D*lrbA*; Km^r	(4)
DH5α/pK19-D*lrbR*	DH5α carrying *lrbR*-deletion plasmid, pK19*mobsacB*-D*lrbR*; Km^r	(4)
DH5α/pK19-*crp*-Flag-knock-in	DH5α carrying CRP-Flag knock-in plasmid, pK19*mobsacB*-*crp*-Flag-knock-in; Km^r	This study
DH5α/pK19-*crp*-HA-knock-in	DH5α carrying CRP-HA knock-in plasmid, pK19*mobsacB*-*crp*-HA-knock-in; Km^r	This study
DH5α/pK19-*lrbA*-Flag-SD-*lrbS*-knock-in	DH5α carrying LrbA-Flag knock-in plasmid, pK19*mobsacB*-*lrbA*-Flag-SD-*lrbS*-knock-in; Km^r	This study
DH5α/pK19-*lrbA*-D54A-Flag-SD-*lrbS*-knock-in	DH5α carrying LrbA-D54A-Flag knock-in plasmid, pK19*mobsacB*-*lrbA*- D54A-Flag-SD-*lrbS*-knock-in; Km^r	This study
DH5α/pK19-*lrbR*-Flag-knock-in	DH5α carrying LrbR-Flag knock-in plasmid, pK19*mobsacB*-*lrbR*-Flag-knock-in; Km^r	This study
DH5α/pK19-D*bpfA*	DH5α carrying *bpfA*-deletion plasmid, pK19*mobsacB*-D*bpfA*; Km^r	(1)
DH5α/pK19-D*bpfD*	DH5α carrying *bpfD*-deletion plasmid, pK19*mobsacB*-D*bpfD*; Km^r	(1)
DH5α/pK19-D*bpfG*	DH5α carrying *bpfG*-deletion plasmid, pK19*mobsacB*-D*bpfG*; Km^r	(1)

DH5α/pK19-D*bpfGbpfD*	DH5α carrying *bpfDbpfG*-deletion plasmid, pK19*mobsacB*-D*bpfDbpfG*; Km^r	This study
DH5α/pK19-*bpfD*-Flag-knock-in	DH5α carrying BpfD-Flag knock-in plasmid, pK19*mobsacB*-*bpfD*-Flag-knock-in; Km^r	(1)
DH5α/pK19-*bpfG*-HA-knock-in	DH5α carrying BpfG--HA knock-in plasmid, pK19*mobsacB*-*bpfG*-HA-knock-in; Km^r	(1)
DH5α/pK19-D*1235*	DH5α carrying *Sputcn32_1235*-deletion plasmid, pK19*mobsacB*-D*Sputcn32_1235*; Km^r	This study
DH5α/pK19-*1235*-Flag-knock-in	DH5α carrying Sputcn32_1235-Flag knock-in plasmid, pK19*mobsacB*-*1235*-Flag-knock-in; Km^r	This study
DH5α/pK19-P_{aacC1}-*lrbR*-D56E-knock-in	DH5α carrying P_{aacC1}-*lrbR*-D56E knock-in plasmid, pK19*mobsacB*- P_{aacC1}-*lrbR*-D56E -Flag-knock-in; Km^r	This study
BL21(DE3)	Protein expression host. *dcm*, *ompT*, *hsdS* ($r_B^- m_B^-$) *gal*	Lab stock
BL21(DE3)/pET28a-CRP	Heterologous expression of N-terminal His_6-tagged *S. putrefaciens* CN32 CRP protein	(1)
BL21(DE3)/pET28a-CRP-R84L	Heterologous expression of N-terminal His_6-tagged *S. putrefaciens* CN32 site-directed CRP-R84L protein	(1)
BL21(DE3)/pET28a-LrbA	Heterologous expression of N-terminal His_6-tagged *S. putrefaciens* CN32 LrbA protein	(4)
BL21(DE3)/pET28a-LrbR	Heterologous expression of N-terminal His_6-tagged *S. putrefaciens* CN32 LrbR protein	This study
BL21(DE3)/pGEX-4T-1-LrbA	Heterologous expression of N-terminal GST-tagged *S. putrefaciens* CN32 LrbA protein	This study
BL21(DE3)/pGEX-4T-1-LrbR	Heterologous expression of N-terminal GST-tagged *S. putrefaciens* CN32 LrbR protein	This study
BL21(DE3)/pGEX-4T-1-BpfD-In	Heterologous expression of N-terminal GST-tagged BpfD of intracellular domain	(1)
Shewanella putrefaciens CN32		
Wild type (WT)	*Shewanella putrefaciens* CN32	(4)
Δ*crp*	CN32 Δ*crp*	(1)
C*crp*	Δ*crp* carrying complement pBBR1MCS-2-P_{aacC1}-*crp*; Km^r	(1)
Δ*cya*	CN32 Δ*cyaA*Δ*cyaB*Δ*cyaC*	(1)
C*cya*	Δ*cya* carrying complement pBBR1MCS-2-P_{aacC1}-*cyaA*-*cyaB*-*cyaC*; Km^r	(1)
Δ*crp*Δ*cya*	CN32 Δ*crp*Δ*cyaA*Δ*cyaB*Δ*cyaC*	(1)
C*crp*-R84L	Δ*crp* carrying complement pBBR1MCS-2-P_{aacC1}-*crp*-R84L; Km^r	(1)
Δ*crp*Δ*cya*C*crp*-R84L	Δ*crp*Δ*cyaA*Δ*cyaB*Δ*cyaC* carrying complement pBBR1MCS-2-P_{aacC1}-*crp*-R84L; Km^r	This study
CRP-R84L	WT with a CRP site mutant from R84 to L84	This study
Δ*lrbA*	CN32 Δ*lrbA*	(4)
Δ*lrbR*	CN32 Δ*lrbR*	(4)
Δ*crp*Δ*lrbA*	CN32 Δ*crp*Δ*lrbA*	This study
Δ*cya*Δ*lrbA*	CN32 Δ*cyaA*Δ*cyaB*Δ*cyaC*Δ*lrbA*	This study
Δ*crp*Δ*lrbR*	CN32 Δ*crp*Δ*lrbR*	(1)

Δ*cya*Δ*lrbR*	CN32 Δ*cyaA*Δ*cyaB*Δ*cyaC*Δ*lrbR*	This study
Δ*crp*Δ*cya*Δ*lrbA*	CN32 Δ*crp*Δ*cyaA*Δ*cyaB*Δ*cyaC*Δ*lrbA*	This study
Δ*crp*Δ*cya*Δ*lrbR*	CN32 Δ*crp*Δ*cyaA*Δ*cyaB*Δ*cyaC*Δ*lrbR*	This study
Δ*crp*Δ*cya*Δ*lrbA*Δ*lrbR*	CN32 Δ*crp*Δ*cyaA*Δ*cyaB*Δ*cyaC*Δ*lrbA*Δ*lrbR*	This study
Δ*lrbS*	CN32 Δ*lrbS*	(4)
Δ*lrbS*Δ*lrbA*	CN32 Δ*lrbS*Δ*lrbA*	(4)
Δ*lrbS*Δ*lrbR*	CN32 Δ*lrbS*Δ*lrbR*	(4)
Δ*lrbA*Δ*lrbR*	CN32 Δ*lrbA*Δ*lrbR*	(4)
Δ*lrbS*Δ*lrbA*Δ*lrbR*	CN32 Δ*lrbS*Δ*lrbA*Δ*lrbR*	(4)
WT/LrbR-Flag	WT with a C-terminal 3×Flag-tagged LrbR	This study
Δ*crp*/LrbR-Flag	Δ*crp* with a C-terminal 3×Flag-tagged LrbR	This study
Δ*cya*/LrbR-Flag	Δ*cya* with a C-terminal 3×Flag-tagged LrbR	This study
Δ*crp*Δ*cya*/LrbR-Flag	Δ*crp*Δ*cya* with a C-terminal 3×Flag-tagged LrbR	This study
Δ*lrbA*/LrbR-Flag	Δ*lrbA* with a C-terminal 3×Flag-tagged LrbR	This study
Δ*crp*Δ*lrbA*/LrbR-Flag	Δ*crp*Δ*lrbA* with a C-terminal 3×Flag-tagged LrbR	This study
CRP-R84L/LrbR-Flag	WT with a CRP site mutant from R84 to L84 and a C-terminal 3×Flag-tagged LrbR	This study
WT/LrbA-Flag	WT with a C-terminal 3×Flag-tagged LrbA	This study
Δ*crp*/LrbA-Flag	Δ*crp* with a C-terminal 3×Flag-tagged LrbA	This study
Δ*cya*/LrbA-Flag	Δ*cya* with a C-terminal 3×Flag-tagged LrbA	This study
Δ*crp*Δ*cya*/LrbA-Flag	Δ*crp*Δ*cya* with a C-terminal 3×Flag-tagged LrbA	This study
WT/LrbA-D54A-Flag	WT with a LrbA site mutant from D54 to A54 and a C-terminal 3×Flag-tagged LrbA	This study
WT/LrbS-Flag	WT with a C-terminal 3×Flag-tagged LrbS	This study
Δ*crp*/LrbS-Flag	Δ*crp* with a C-terminal 3×Flag-tagged LrbS	This study
Δ*cya*/LrbS-Flag	Δ*cya* with a C-terminal 3×Flag-tagged LrbS	This study
Δ*crp*Δ*cya*/LrbS-Flag	Δ*crp*Δ*cya* with a C-terminal 3×Flag-tagged LrbS	This study
WT/CRP-Flag	WT with a C-terminal 3×Flag-tagged CRP	This study
Δ*lrbA*/CRP-Flag	Δ*lrbA* with a C-terminal 3×Flag-tagged CRP	This study
WT/CRP-Flag/p*dgcQ*	WT with a C-terminal 3×Flag-tagged CRP and carrying pBBR1MCS-2-P_{aacC1}-*dgcQ*$_{\mathrm{MG1655}}$; Km^r	This study
WT/LrbA-Flag	WT with a C-terminal 3×Flag-tagged LrbA	This study
Δ*crp*/LrbA-Flag	Δ*crp* with a C-terminal 3×Flag-tagged LrbA	This study
WT/LrbA-Flag/CRP-HA	WT with a C-terminal 3×Flag-tagged LrbA and a C-terminal 1×HA-tagged CRP	This study
Δ*cya*/LrbA-Flag/CRP-HA	Δ*cya* with a C-terminal 3×Flag-tagged LrbA and a C-terminal 1×HA-tagged CRP	This study
C*lrbA*-D54E	Δ*lrbA* carrying pBBR1MCS-2-P_{aacC1}-*lrbA*-D54E; Km^r	(4)
C*lrbA*-D54A	Δ*lrbA* carrying pBBR1MCS-2-P_{aacC1}-*lrbA*-D54A; Km^r	(4)
WT/p*lrbA*-D54E	WT carrying pBBR1MCS-2-P_{aacC1}-*lrbA*-D54E; Km^r	This study
Δ*crp*/p*lrbA*-D54E	Δ*crp* carrying pBBR1MCS-2-P_{aacC1}-*lrbA*-D54E; Km^r	This study
WT/p*crp*	WT carrying pBBR1MCS-2-P_{aacC1}-*crp*; Km^r	This study
Δ*lrbA*/p*crp*	Δ*lrbA* carrying pBBR1MCS-2-P_{aacC1}-*crp*; Km^r	This study
WT/LrbR-Flag/p*lrbA*-D54E	WT with a C-terminal 3×Flag-tagged LrbR and carrying	This study

	pBBR1MCS-2-P_{aacC1}-*lrbA*-D54E; Km^r	
Δ*crp*/LrbR-Flag/p*lrbA*-D54E	Δ*crp* with a C-terminal 3×Flag-tagged LrbR and carrying pBBR1MCS-2-P_{aacC1}-*lrbA*-D54E; Km^r	This study
Δ*cya*/LrbR-Flag/p*lrbA*-D54E	Δ*cya* with a C-terminal 3×Flag-tagged LrbR and carrying pBBR1MCS-2-P_{aacC1}-*lrbA*-D54E; Km^r	This study
CRP-R84L/LrbR-Flag/p*lrbA*-D54E	WT with a CRP site mutant from R84 to L84 and with a C-terminal 3×Flag-tagged LrbR and carrying pBBR1MCS-2-P_{aacC1}-*lrbA*-D54E; Km^r	This study
WT/LrbR-Flag/p*crp*	WT with a C-terminal 3×Flag-tagged LrbR and carrying pBBR1MCS-2-P_{aacC1}-*crp*; Km^r	This study
Δ*lrbA*/LrbR-Flag/p*crp*	Δ*lrbA* with a C-terminal 3×Flag-tagged LrbR and carrying pBBR1MCS-2-P_{aacC1}-*crp*; Km^r	This study
Δ*bpfA*	CN32 Δ*bpfA*	(1)
Δ*crp*Δ*bpfA*	CN32 Δ*crp*Δ*bpfA*	(1)
Δ*lrbR*Δ*bpfA*	CN32 Δ*lrbR*Δ*bpfA*	This study
Δ*crp*Δ*lrbR*Δ*bpfA*	CN32 Δ*crp*Δ*lrbR*Δ*bpfA*	This study
Δ*bpfD*	CN32 Δ*bpfD*	(1)
Δ*crp*Δ*bpfD*	CN32 Δ*crp*Δ*bpfD*	(1)
Δ*lrbR*Δ*bpfD*	CN32 Δ*lrbR*Δ*bpfD*	This study
Δ*crp*Δ*lrbR*Δ*bpfD*	CN32 Δ*crp*Δ*lrbR*Δ*bpfD*	This study
Δ*bpfG*	CN32 Δ*bpfG*	(1)
Δ*crp*Δ*bpfG*	CN32 Δ*crp*Δ*bpfG*	(1)
Δ*lrbR*Δ*bpfG*	CN32 Δ*lrbR*Δ*bpfG*	This study
Δ*crp*Δ*lrbR*Δ*bpfG*	CN32 Δ*crp*Δ*lrbR*Δ*bpfG*	This study
Δ*bpfG*Δ*bpfA*	CN32 Δ*bpfG*Δ*bpfA*	This study
Δ*crp*Δ*bpfG*Δ*bpfA*	CN32 Δ*crp*Δ*bpfG*Δ*bpfA*	This study
Δ*lrbR*Δ*bpfG*Δ*bpfA*	CN32 Δ*lrbR*Δ*bpfG*Δ*bpfA*	This study
Δ*crp*Δ*lrbR*Δ*bpfG*Δ*bpfA*	CN32 Δ*crp*Δ*lrbR*Δ*bpfG*Δ*bpfA*	This study
Δ*bpfD*Δ*bpfG*	CN32 Δ*bpfD*Δ*bpfG*	This study
Δ*crp*Δ*bpfD*Δ*bpfG*	CN32 Δ*crp*Δ*bpfD*Δ*bpfG*	This study
Δ*lrbR*Δ*bpfD*Δ*bpfG*	CN32 Δ*lrbR*Δ*bpfD*Δ*bpfG*	This study
Δ*crp*Δ*lrbR*Δ*bpfD*Δ*bpfG*	CN32 Δ*crp*Δ*lrbR*Δ*bpfD*Δ*bpfG*	This study
WT/BpfA-FLAG	WT with a BpfA that 3×Flag inserted after residue 3700 aa (11100 bp) in the full-length protein of 4220 aa transformant	(1)
Δ*crp*/BpfA-FLAG	Δ*crp* with a BpfA that 3×Flag inserted after residue 3700 aa (11100 bp) in the full-length protein of 4220 aa transformant	(1)
Δ*cya*/BpfA-FLAG	Δ*cya* with a BpfA that 3×Flag inserted after residue 3700 aa (11100 bp) in the full-length protein of 4220 aa	This study
Δ*lrbR*/BpfA-FLAG	Δ*lrbR* with a BpfA that with 3×Flag inserted after residue 3700 aa (11100 bp) in the full-length protein of 4220 aa transformant	This study
WT/BpfA-FLAG/p*dgcQ*	WT with a BpfA that 3×Flag inserted after residue 3700 aa (11100 bp) in the full-length protein of 4220 aa transformant and carrying pBBR1MCS-2-P_{aacC1}-*dgcQ*$_{MG1655}$; Km^r	This study
WT/P_{aacC1}-*bpfA*/BpfA-Flag	WT replacing the promoter region of *bpfA* operon with *aacC1*	(1)

	promoter and BpfA with 3×Flag inserted after residue 3700 aa (11100 bp) in the full-length protein of 4220 aa	
Δ*crp*/P$_{aacC1}$-*bpfA*/BpfA-Flag	Δ*crp* replacing the promoter region of *bpfA* operon with *aacC1* promoter and BpfA with 3×Flag inserted after residue 3700 aa (11100 bp) in the full-length protein of 4220 aa	(1)
Δ*cya*/P$_{aacC1}$-*bpfA*/BpfA-Flag	Δ*cyaA*Δ*cyaB*Δ*cyaC* replacing the promoter region of *bpfA* operon with *aacC1* promoter and BpfA with 3×Flag inserted after residue 3700 aa (11100 bp) in the full-length protein of 4220 aa	(1)
Δ*lrbR*/P$_{aacC1}$-*bpfA*/BpfA-Flag	Δ*lrbR* replacing the promoter region of *bpfA* operon with *aacC1* promoter and BpfA with 3×Flag inserted after residue 3700 aa (11100 bp) in the full-length protein of 4220 aa	This study
WT/P$_{aacC1}$-*bpfA*	WT replacing the promoter region of *bpfA* operon with *aacC1* promoter	(1)
Δ*crp*/P$_{aacC1}$-*bpfA*	Δ*crp* replacing the promoter region of *bpfA* operon with *aacC1* promoter	(1)
Δ*cya*/P$_{aacC1}$-*bpfA*	Δ*cyaA*Δ*cyaB*Δ*cyaC* replacing the promoter region of *bpfA* operon with *aacC1* promoter	(1)
Δ*lrbR*/P$_{aacC1}$-*bpfA*	Δ*lrbR* replacing the promoter region of *bpfA* operon with *aacC1* promoter	This study
WT/P$_{aacC1}$-*bpfA*/p*dgcQ*	WT replacing the promoter region of *bpfA* operon with *aacC1* promoter and carrying pBBR1MCS-2-P$_{aacC1}$-*dgcQ*$_{MG1655}$; Km^{r}	This study
WT/P$_{aacC1}$-*bpfA*/BpfA-FLAG	WT replacing the promoter region of *bpfA* operon with *aacC1* promoter and BpfA with 3×Flag inserted after residue 3700 aa (11100 bp) in the full-length protein of 4220 aa transformant	(1)
Δ*crp*/P$_{aacC1}$-*bpfA*/BpfA-FLAG	Δ*crp* replacing the promoter region of *bpfA* operon with *aacC1* promoter and BpfA with 3×Flag inserted after residue 3700 aa (11100 bp) in the full-length protein of 4220 aa transformant	(1)
Δ*cya*/P$_{aacC1}$-*bpfA*/BpfA-FLAG	Δ*cyaA*Δ*cyaB*Δ*cyaC* replacing the promoter region of *bpfA* operon with *aacC1* promoter and BpfA with 3×Flag inserted after residue 3700 aa (11100 bp) in the full-length protein of 4220 aa transformant	(1)
Δ*lrbR*/P$_{aacC1}$-*bpfA*/BpfA-FLAG	Δ*lrbR* replacing the promoter region of *bpfA* operon with *aacC1* promoter and BpfA with 3×Flag inserted after residue 3700 aa (11100 bp) in the full-length protein of 4220 aa transformant	This study
WT/p*dgcQ*	WT carrying pBBR1MCS-2-P$_{aacC1}$-*dgcQ*$_{MG1655}$; Km^{r}	(1)
Δ*crp*/p*dgcQ*	Δ*crp* carrying pBBR1MCS-2-P$_{aacC1}$-*dgcQ*$_{MG1655}$; Km^{r}	(1)
Δ*cya*/p*dgcQ*	Δ*cyaA*Δ*cyaB*Δ*cyaC* carrying pBBR1MCS-2-P$_{aacC1}$-*dgcQ*$_{MG1655}$; Km^{r}	(1)
Δ*lrbR*/p*dgcQ*	Δ*lrbR* carrying pBBR1MCS-2-P$_{aacC1}$-*dgcQ*$_{MG1655}$; Km^{r}	This study
Δ*bpfA*/p*dgcQ*	Δ*bpfA* carrying pBBR1MCS-2-P$_{aacC1}$-*dgcQ*$_{MG1655}$; Km^{r}	(1)
Δ*bpfD*/p*dgcQ*	Δ*bpfD* carrying pBBR1MCS-2-P$_{aacC1}$-*dgcQ*$_{MG1655}$; Km^{r}	This study
Δ*bpfG*/p*dgcQ*	Δ*bpfG* carrying pBBR1MCS-2-P$_{aacC1}$-*dgcQ*$_{MG1655}$; Km^{r}	This study
WT/P$_{aacC1}$-*bpfA*/BpfA-FLAG/p*dgcQ*	WT replacing the promoter region of *bpfA* operon with *aacC1* promoter and BpfA with 3×Flag inserted after residue 3700 aa	This study

	(11100 bp) in the full-length protein of 4220 aa transformant and carrying pBBR1MCS-2-P_{aacC1}-*dgcQ*$_{MG1655}$; Km^r	
WT/P_{aacC1}-*bpfA*/BpfD-FLAG/BpfG-HA	WT replacing the promoter region of *bpfA* operon with *aacC1* promoter, and with a C-terminal 3×Flag-tagged BpfD, and BpfG with 1×HA inserted after residue 221 aa (663 bp) in the full-length protein of 235 aa transformant	(1)
Δ*crp*/P_{aacC1}-*bpfA*/BpfD-FLAG/BpfG-HA	Δ*crp* replacing the promoter region of *bpfA* operon with *aacC1* promoter, and with a C-terminal 3×Flag-tagged BpfD, and BpfG with 1×HA inserted after residue 221 aa (663 bp) in the full-length protein of 235 aa transformant	(1)
Δ*lrbR*/P_{aacC1}-*bpfA*/BpfD-FLAG/BpfG-HA	Δ*lrbR* replacing the promoter region of *bpfA* operon with *aacC1* promoter, and with a C-terminal 3×Flag-tagged BpfD, and BpfG with 1×HA inserted after residue 221 aa (663 bp) in the full-length protein of 235 aa transformant	This study
WT/P_{aacC1}-*bpfA*/BpfD-FLAG/BpfG-HA/p*dgcQ*	WT replacing the promoter region of *bpfA* operon with *aacC1* promoter, and with a C-terminal 3×Flag-tagged BpfD, and BpfG with 1×HA inserted after residue 221 aa (663 bp) in the full-length protein of 235 aa transformant, and carrying pBBR1MCS-2-P_{aacC1}-*dgcQ*$_{MG1655}$; Km^r	This study
WT/P_{aacC1}-*bpfA*/BpfD-FLAG	WT replacing the promoter region of *bpfA* operon with *aacC1* promoter, and with a C-terminal 3×Flag-tagged BpfD	(1)
WT/P_{aacC1}-*bpfA*/BpfD-FLAG/LrbR-HA	WT replacing the promoter region of *bpfA* operon with *aacC1* promoter, and with a C-terminal 3×Flag-tagged BpfD and a C-terminal 1×HA-tagged LrbR	This study
WT/P_{aacC1}-*bpfA*/BpfD-FLAG/CRP-HA	WT replacing the promoter region of *bpfA* operon with *aacC1* promoter, and with a C-terminal 3×Flag-tagged BpfD and a C-terminal 1×HA-tagged CRP	This study
WT/LrbR-FLAG/CRP-HA	WT with a C-terminal 3×Flag-tagged LrbR and a C-terminal 1×HA-tagged CRP	This study
WT/p*dgcQ*-*lrbA*-D54E	WT carrying pBBR1MCS-2-P_{aacC1}-*dgcQ*$_{MG1655}$-*lrbA*-D54E; Km^r	This study
Δ*crp*/p*dgcQ*-*lrbA*-D54E	Δ*crp* carrying pBBR1MCS-2-P_{aacC1}-*dgcQ*$_{MG1655}$-*lrbA*-D54E; Km^r	This study
Δ*cya*/p*dgcQ*-*lrbA*-D54E	Δ*cya* carrying pBBR1MCS-2-P_{aacC1}-*dgcQ*$_{MG1655}$-*lrbA*-D54E; Km^r	This study
CRP-R84L/p*dgcQ*-*lrbA*-D54E	WT with a CRP site mutant from R84 to L84 and carrying pBBR1MCS-2-P_{aacC1}-*dgcQ*$_{MG1655}$-*lrbA*-D54E; Km^r	This study
WT/LrbR-Flag/p*dgcQ*-*lrbA*-D54E	WT with a C-terminal 3×Flag-tagged LrbR and carrying pBBR1MCS-2-P_{aacC1}-*dgcQ*$_{MG1655}$-*lrbA*-D54E; Km^r	This study
Δ*crp*/LrbR-Flag/p*dgcQ*-*lrbA*-D54E	Δ*crp* with a C-terminal 3×Flag-tagged LrbR and carrying pBBR1MCS-2-P_{aacC1}-*dgcQ*$_{MG1655}$-*lrbA*-D54E; Km^r	This study
Δ*cya*/LrbR-Flag/p*dgcQ*-*lrbA*-D54E	Δ*cya* with a C-terminal 3×Flag-tagged LrbR and carrying pBBR1MCS-2-P_{aacC1}-*dgcQ*$_{MG1655}$-*lrbA*-D54E; Km^r	This study
CRP-R84L/LrbR-Flag/p*dgcQ*-*lrbA*-D54E	WT with a CRP site mutant from R84 to L84 and a C-terminal 3×Flag-tagged LrbR and carrying pBBR1MCS-2-P_{aacC1}-*dgcQ*$_{MG1655}$-*lrbA*-D54E; Km^r	This study

WT/p*dgcQ*-*lrbA*-D54E	WT carrying pBBR1MCS-2-P$_{aacC1}$-*dgcQ*$_{MG1655}$-*lrbA*-D54E; Km^r	This study
Δ*crp*/p*dgcQ*-*lrbA*-D54E	Δ*crp* carrying pBBR1MCS-2-P$_{aacC1}$-*dgcQ*$_{MG1655}$-*lrbA*-D54E; Km^r	This study
Δ*cya*/p*dgcQ*-*lrbA*-D54E	Δ*cya* carrying pBBR1MCS-2-P$_{aacC1}$-*dgcQ*$_{MG1655}$-*lrbA*-D54E; Km^r	This study
CRP-R84L/p*dgcQ*-*lrbA*-D54E	WT with a CRP site mutant from R84 to L84 and carrying pBBR1MCS-2-P$_{aacC1}$-*dgcQ*$_{MG1655}$-*lrbA*-D54E; Km^r	This study
WT/P$_{aacC1}$-*bpfA*/BpfD-FLAG/BpfG-HA/ p*dgcQ*-*lrbA*-D54E	WT replacing the promoter region of *bpfA* operon with *aacC1* promoter, and with a C-terminal 3×Flag-tagged BpfD, and BpfG with 1×HA inserted after residue 221 aa (663 bp) in the full-length protein of 235 aa transformant, and carrying pBBR1MCS-2-P$_{aacC1}$-*dgcQ*$_{MG1655}$-*lrbA*-D54E; Km^r	This study
Δ*crp*//P$_{aacC1}$-*bpfA*/BpfD-FLAG/BpfG-HA/ p*dgcQ*-*lrbA*-D54E	Δ*crp* replacing the promoter region of *bpfA* operon with *aacC1* promoter, and with a C-terminal 3×Flag-tagged BpfD, and BpfG with 1×HA inserted after residue 221 aa (663 bp) in the full-length protein of 235 aa transformant, and carrying pBBR1MCS-2-P$_{aacC1}$-*dgcQ*$_{MG1655}$-*lrbA*-D54E; Km^r	This study
Δ*cya*//P$_{aacC1}$-*bpfA*/BpfD-FLAG/BpfG-HA/ p*dgcQ*-*lrbA*-D54E	Δ*cya* replacing the promoter region of *bpfA* operon with *aacC1* promoter, and with a C-terminal 3×Flag-tagged BpfD, and BpfG with 1×HA inserted after residue 221 aa (663 bp) in the full-length protein of 235 aa transformant, and carrying pBBR1MCS-2-P$_{aacC1}$-*dgcQ*$_{MG1655}$-*lrbA*-D54E; Km^r	This study
CRP-R84L//P$_{aacC1}$-*bpfA*/BpfD-FLAG/BpfG-HA/ p*dgcQ*-*lrbA*-D54E	WT with a CRP site mutant from R84 to L84 and replacing the promoter region of *bpfA* operon with *aacC1* promoter, and with a C-terminal 3×Flag-tagged BpfD, and BpfG with 1×HA inserted after residue 221 aa (663 bp) in the full-length protein of 235 aa transformant, and carrying pBBR1MCS-2-P$_{aacC1}$-*dgcQ*$_{MG1655}$-*lrbA*-D54E; Km^r	This study
WT/P$_{aacC1}$-*bpfA*/BpfA-FLAG/p*dgcQ*-*lrbA*-D54E	WT replacing the promoter region of *bpfA* operon with *aacC1* promoter and BpfA with 3×Flag inserted after residue 3700 aa (11100 bp) in the full-length protein of 4220 aa transformant, and carrying pBBR1MCS-2-P$_{aacC1}$-*dgcQ*$_{MG1655}$-*lrbA*-D54E; Km^r	This study
Δ*crp*/P$_{aacC1}$-*bpfA*/BpfA-FLAG/p*dgcQ*-*lrbA*-D54E	Δ*crp* replacing the promoter region of *bpfA* operon with *aacC1* promoter and BpfA with 3×Flag inserted after residue 3700 aa (11100 bp) in the full-length protein of 4220 aa transformant, and carrying pBBR1MCS-2-P$_{aacC1}$-*dgcQ*$_{MG1655}$-*lrbA*-D54E; Km^r	This study
Δ*cya*/P$_{aacC1}$-*bpfA*/BpfA-FLAG/p*dgcQ*-*lrbA*-D54E	Δ*cyaA*Δ*cyaB*Δ*cyaC* replacing the promoter region of *bpfA* operon with *aacC1* promoter and BpfA with 3×Flag inserted after residue 3700 aa (11100 bp) in the full-length protein of 4220 aa transformant, and carrying pBBR1MCS-2-P$_{aacC1}$-*dgcQ*$_{MG1655}$-*lrbA*-D54E; Km^r	This study
CRP-R84L/P$_{aacC1}$-*bpfA*/BpfA-FLAG/p*dgcQ*-*lrbA*-D54E	WT with a CRP site mutant from R84 to L84 and replacing the promoter region of *bpfA* operon with *aacC1* promoter and BpfA with 3×Flag inserted after residue 3700 aa (11100 bp) in the full-length protein of 4220 aa transformant, and carrying	This study

	pBBR1MCS-2-P$_{aacC1}$-*dgcQ*$_{\mathrm{MG1655}}$-*lrbA*-D54E; Km^r	
WT/P$_{aacC1}$-*bpfA*/BpfD-FLAG/LrbR-HA/p*lrbA*-D54E	WT replacing the promoter region of *bpfA* operon with *aacC1* promoter, and with a C-terminal 3×Flag-tagged BpfD and a C-terminal 1×HA-tagged LrbR and carrying pBBR1MCS-2-P$_{aacC1}$-*lrbA*-D54E; Km^r	This study
Δ*crp*/P$_{aacC1}$-*bpfA*/BpfD-FLAG/LrbR-HA/p*lrbA*-D54E	Δ*crp* replacing the promoter region of *bpfA* operon with *aacC1* promoter, and with a C-terminal 3×Flag-tagged BpfD and a C-terminal 1×HA-tagged LrbR and carrying pBBR1MCS-2-P$_{aacC1}$-*lrbA*-D54E; Km^r	This study
Δ*cya*/P$_{aacC1}$-*bpfA*/BpfD-FLAG/LrbR-HA/p*lrbA*-D54E	Δ*cya* replacing the promoter region of *bpfA* operon with *aacC1* promoter, and with a C-terminal 3×Flag-tagged BpfD and a C-terminal 1×HA-tagged LrbR and carrying pBBR1MCS-2-P$_{aacC1}$-*lrbA*-D54E; Km^r	This study
CRP-R84L/P$_{aacC1}$-*bpfA*/BpfD-FLAG/LrbR-HA/ p*lrbA*-D54E	WT with a CRP site mutant from R84 to L84 and replacing the promoter region of *bpfA* operon with *aacC1* promoter, and with a C-terminal 1×Flag-tagged BpfD and a C-terminal 1×HA-tagged LrbR and carrying pBBR1MCS-2-P$_{aacC1}$-*lrbA*-D54E; Km^r	This study
WT/P$_{aacC1}$-*bpfA*/BpfD-FLAG/LrbR-HA/ p*dgcQ*-*lrbA*-D54E	WT replacing the promoter region of *bpfA* operon with *aacC1* promoter, and with a C-terminal 3×Flag-tagged BpfD and a C-terminal 1×HA-tagged LrbR and carrying pBBR1MCS-2-P$_{aacC1}$-*dgcQ*$_{\mathrm{MG1655}}$-*lrbA*-D54E; Km^r	This study
Δ*crp*/P$_{aacC1}$-*bpfA*/BpfD-FLAG/LrbR-HA/ p*dgcQ*-*lrbA*-D54E	Δ*crp* replacing the promoter region of *bpfA* operon with *aacC1* promoter, and with a C-terminal 3×Flag-tagged BpfD and a C-terminal 1×HA-tagged LrbR and carrying pBBR1MCS-2-P$_{aacC1}$-*dgcQ*$_{\mathrm{MG1655}}$-*lrbA*-D54E; Km^r	This study
Δ*cya*/P$_{aacC1}$-*bpfA*/BpfD-FLAG/LrbR-HA/ p*dgcQ*-*lrbA*-D54E	Δ*cya* replacing the promoter region of *bpfA* operon with *aacC1* promoter, and with a C-terminal 3×Flag-tagged BpfD and a C-terminal 1×HA-tagged LrbR and carrying pBBR1MCS-2-P$_{aacC1}$-*dgcQ*$_{\mathrm{MG1655}}$-*lrbA*-D54E; Km^r	This study
CRP-R84L/P$_{aacC1}$-*bpfA*/BpfD-FLAG/LrbR-HA/ p*dgcQ*-*lrbA*-D54E	WT with a CRP site mutant from R84 to L84 and replacing the promoter region of *bpfA* operon with *aacC1* promoter, and with a C-terminal 3×Flag-tagged BpfD and a C-terminal 1×HA-tagged LrbR and carrying pBBR1MCS-2-P$_{aacC1}$-*dgcQ*$_{\mathrm{MG1655}}$-*lrbA*-D54E; Km^r	This study
WT/LrbR-FLAG/CRP-HA/p*dgcQ*-*lrbA*-D54E	WT with a C-terminal 3×Flag-tagged LrbR and a C-terminal 1×HA-tagged CRP and carrying pBBR1MCS-2-P$_{aacC1}$-*dgcQ*$_{\mathrm{MG1655}}$-*lrbA*-D54E; Km^r	This study
Δ*cya*/LrbR-FLAG/CRP-HA/p*dgcQ*-*lrbA*-D54E	Δ*cya* with a C-terminal 3×Flag-tagged LrbR and a C-terminal 1×HA-tagged CRP and carrying pBBR1MCS-2-P$_{aacC1}$-*dgcQ*$_{\mathrm{MG1655}}$-*lrbA*-D54E; Km^r	This study
CRP-R84L/LrbR-FLAG/CRP-HA/p*dgcQ*-*lrbA*-D54E	WT with a CRP site mutant from R84 to L84 and with a C-terminal 3×Flag-tagged LrbR and a C-terminal 1×HA-tagged CRP and carrying pBBR1MCS-2-P$_{aacC1}$-*dgcQ*$_{\mathrm{MG1655}}$-*lrbA*-D54E; Km^r	This study

Δ0099	CN32 Δ*Sputcn32_0099*	This study
Δ0133	CN32 Δ*Sputcn32_0133*	(1)
Δ0327	CN32 Δ*Sputcn32_0327*	This study
Δ0384	CN32 Δ*Sputcn32_0384*	This study
Δ0414	CN32 Δ*Sputcn32_0414*	This study
Δ0555	CN32 Δ*Sputcn32_0555*	This study
Δ0601	CN32 Δ*Sputcn32_0601*	This study
Δ0654	CN32 Δ*Sputcn32_0654*	(1)
Δ1039	CN32 Δ*Sputcn32_1039*	This study
Δ1235	CN32 Δ*Sputcn32_1235*	This study
Δ1253	CN32 Δ*Sputcn32_1253*	This study
Δ1291	CN32 Δ*Sputcn32_1291*	(1)
Δ1365	CN32 Δ*Sputcn32_1365*	(1)
Δ1412	CN32 Δ*Sputcn32_1412*	(1)
Δ1741	CN32 Δ*Sputcn32_1741*	This study
Δ1800	CN32 Δ*Sputcn32_1800*	This study
Δ1851	CN32 Δ*Sputcn32_1851*	This study
Δ1858	CN32 Δ*Sputcn32_1858*	(1)
Δ1917	CN32 Δ*Sputcn32_1917*	This study
Δ1934	CN32 Δ*Sputcn32_1934*	(1)
Δ2096	CN32 Δ*Sputcn32_2096*	This study
Δ2106	CN32 Δ*Sputcn32_2106*	This study
Δ2456	CN32 Δ*Sputcn32_2456*	This study
Δ2671	CN32 Δ*Sputcn32_2671*	This study
Δ2800	CN32 Δ*Sputcn32_2800*	This study
Δ2830	CN32 Δ*Sputcn32_2830*	This study
Δ3018	CN32 Δ*Sputcn32_3018*	This study
Δ3085	CN32 Δ*Sputcn32_3085*	This study
Δ3168	CN32 Δ*Sputcn32_3168*	This study
ΔdosD	CN32 Δ*Sputcn32_3244*	(7)
Δ3269	CN32 Δ*Sputcn32_3269*	This study
Δ3306	CN32 Δ*Sputcn32_3306*	This study
Δ3319	CN32 Δ*Sputcn32_3319*	(1)
Δ3328	CN32 Δ*Sputcn32_3328*	(1)
Δ3390	CN32 Δ*Sputcn32_3390*	This study
ΔpdeB	CN32 Δ*Sputcn32_3405*	(8)
Δ3598	CN32 Δ*Sputcn32_3598*	(1)
Δ3648	CN32 Δ*Sputcn32_3648*	This study
Δ3856	CN32 Δ*Sputcn32_3856*	This study
Δ3917	CN32 Δ*Sputcn32_3917*	This study
Δ0814	CN32 Δ*Sputcn32_0814*	This study
Δ3141	CN32 Δ*Sputcn32_3141*	This study
Δ1988	CN32 Δ*Sputcn32_1988*	This study
Δ1344	CN32 Δ*Sputcn32_1344*	This study

Δ*2362*	CN32 Δ*Sputcn32_2362*	This study
Δ*crp*ΔDGC2/C-DGC2	CN32 Δ*crp*Δ*Sputcn32_1291*Δ*Sputcn32_3328* carrying pBBR1MCS-2-P$_{aacC1}$-*Sputcn32_1291- Sputcn32_3328*; Km^r	(1)
WT/BpfD-FLAG	WT with a C-terminal 3×Flag-tagged BpfD	This study
WT/BpfD-FLAG/p*dgcQ*	WT with a C-terminal 3×Flag-tagged BpfD and carrying pBBR1MCS-2-P$_{aacC1}$-*dgcQ*$_{\mathrm{MG1655}}$-*lrbA*-D54E; Km^r	This study
WT/BpfG-HA	WT with a BpfG that 1×HA inserted after residue 221 aa (663 bp) in the full-length protein of 235 aa transformant	This study
WT/BpfG-HA/p*dgcQ*	WT with a BpfG that 1×HA inserted after residue 221 aa (663 bp) in the full-length protein of 235 aa transformant and carrying pBBR1MCS-2-P$_{aacC1}$-*dgcQ*$_{\mathrm{MG1655}}$; Km^r	This study
Δ*lrbR*/ P$_{aacC1}$-*lrbR*-D56E	Δ*lrbR* with a knocked-in *lrbR*-D56E driven by P$_{aacC1}$	This study
WT/p*lrbA*	WT carrying pBBR1MCS-2-P$_{aacC1}$-*lrbA*; Km^r	This study
Δ*crp*/p*lrbA*	Δ*crp* carrying pBBR1MCS-2-P$_{aacC1}$-*lrbA*; Km^r	This study
Plasmids		
pET-28a(+)	Vector for heterologous protein expression in *E. coli*; Km^r	Novagen
pK19*mobsacB*	Suicide plasmid for CN32; *sacB* (modified from *B. subtilis*) *lacZ*; Km^r	(2)
pBBR1MCS-2	Broad host range vector; Km^r	(3)
pBBR1MCS-2-P$_{aacC1}$	Broad host range vector with a constitutive promoter P$_{aacC1}$; Km^r	(4)
pGEX-4T-1	Vector for heterologous protein expression in *E. coli*; Amp^r	GE healthcare
pMV-Flag	Vector containing 3×FLAG fragment, Amp^r	Lab stock

Table S2. Primers used in this study

Primer	Sequence (5' to 3')	Target gene
lrbR-QF	TGATTTTGGTGCAGCCCATTCCTC	*lrbR* qRT-PCR
lrbR-QR	CGCAGCTTGACATCTTGGTTCAAT	*lrbR* qRT-PCR
lrbA-*lrbR*-inter-EMSA-F	AGGGTGATCATCCACTATCAATAT	EMSA probe primer
lrbA-*lrbR*-inter-EMSA-R	GATCCTCTAATACAATGACCCTAAT	EMSA probe primer
lrbR-Foot-F[b]	TGTAAAACGACGGCCAGTTGATACACATTCTAACCGTTTG	DNase I footprinting probe primer
lrbR-Foot-R	CAGGAAACAGCTATGACCGATCCTCTAATACAATGACCCTAAT	DNase I footprinting probe primer
lrbR-ChIP-F	TTAGGTAAGTAATAGTGGGTTACC	ChIP-qPCR
lrbR-ChIP-R	AATATATACATACGTTACTGCACG	ChIP-qPCR
lrbA-QF	TTCCACTCGTGAGATGGTCGTCCT	*lrbA* qRT-PCR
lrbA-QR	TTACTTAATAGCATACGGTCGGCA	*lrbA* qRT-PCR
lrbS-QF	GTGGCACTGGCCTAGGTTTAGCCA	*lrbS* qRT-PCR
lrbS-QR	GATACAAGGGCAGACGCATACTGA	*lrbS* qRT-PCR
bpfA-QF	ACCATCGCACCCAATGAGTT	*bpfA* qRT-PCR
bpfA-QR	CTGCATCGCTGTCATTGTCG	*bpfA* qRT-PCR
1235-comF	ATTCTTGGATCCACTGGTCAATCACGGTAATAATTA	*1235* complemented
1235-comR	CACACTGAATTCCTTACGACATGCTGGCTACGACAT	*1235* complemented
1235-GGAAF-F[a]	CTCGTATTGGTGGCGCTGCTTTTGCTATT	*1235*-GGAAF amplification
1235-GGAAF-R[a]	CAAATACATCACCGTTACGGATTTGGGAT	*1235*-GGAAF amplification
lrbA-comF	CGGAATTCACCCACTATTACTTACCTAACC	*lrbA*-D54E amplification
lrbA-ER	CCGCTCGAGTCATATGATGCTGTTTCGCTTCGC	*lrbA*-D54E amplification or heterologous expression of LrbA protein in *E. coli*
lrbA-EF	CGGGATCCATGAAAAGAAAAATATTGATAGTGGATG	Heterologous expression of LrbA protein in *E. coli*
lrbA-D54A-F[a]	GATGCAGTTATTCTAGCAATAGGTATTCCT	*lrbA*-D54A knock-in
lrbA-D54-R[a]	GGGAGATAAATCTTTAACGAGTTTCAGTGC	*lrbA*-D54A knock-in
lrbA-D54-SF	CATAAGAAACCCTACACACATGCT	*lrbA*-D54A knock-in
lrbA-D54-SR	GTGGTTGTTTTAGCACTCAAGATC	*lrbA*-D54A knock-in
lrbR-EF	CGGGATCCATGGGTAAGATTAGGGTCATTGTA	Heterologous expression of LrbA protein in *E. coli*
lrbR-ER1	CCCAAGCTTTCCGTTATAGAGAGTTATTATTAGCCATTG	Heterologous expression of LrbA protein in *E. coli*
lrbR-ER2	CGGAATTCTCCGTTATAGAGAGTTATTATTAGCCATTG	Heterologous expression of LrbA protein in *E. coli*
CRP-R84L-F[a]	GAGCTAAACAAGCATGTGAAATTGCAGAA	*crp*-R84L knock-in
CRP-R84L-R[a]	GAACCCAAGCGGTTAATTCTGCTTG	*crp*-R84L knock-in

crp-UF	GTTGGATACACCAGTGCGAACAGAC	*crp* deletion
crp-DR	TCTAAACTAAGACTTCTATCAAGTT	*crp* deletion
crp-InF	CCAATCTCTTGACGAGTGATCTTG	*crp* deletion
crp-InR	AAGGTTCTGTTGCCGTATTGATTAA	*crp* deletion
crp-OF	CCAGCATGATATGTTCAAGATCTT	*crp* deletion
crp-OR	GCAGCACTAAAATCACCAATTTCT	*crp* deletion
cyaA-UF	TGTAGATGGCTCTAAGATTGTGATC	*cyaA* deletion
cyaA-DR	AGGATGGATCAGCAAGACCTATTTC	*cyaA* deletion
cyaA-InF	AATCCTGAACCAGTTGCTGCATT	*cyaA* deletion
cyaA-InR	ACCTTGGTGATGTCCATGAGTTAC	*cyaA* deletion
cyaA-OF	AATGGTTGGATCTCAGTGAGTTGTG	*cyaA* deletion
cyaA-OR	CTTGGCATCCACCTTCTAAGCGAG	*cyaA* deletion
cyaB-UF	CGCAATACACAGCAACACGGAGTAT	*cyaB* deletion
cyaB-UR	ATGCACCACTGTTATTGTTAGATG	*cyaB* deletion
cyaB-InF	CATACATGGCTAATAAGGATTCATC	*cyaB* deletion
cyaB-InR	GATAACCTTGAGCATGATTGTTAC	*cyaB* deletion
cyaB-OF	TTTCAAAGTGATACGAGGGGATTG	*cyaB* deletion
cyaB-OR	CCAAGCCATGTTGATCCTCTCCAC	*cyaB* deletion
cyaC-UF	AGGCTCCTTACCGAGATATAGGAC	*cyaC* deletion
cyaC-DR	CACTAGCACTTTGGTGATGATTTTC	*cyaC* deletion
cyaC-InF	TAAGGACGGCATCACCCATGTAAC	*cyaC* deletion
cyaC-InR	AAACCATATGTCTACGCTAAGGCA	*cyaC* deletion
cyaC-OF	CTGAATGGCGATTCTGCTCACACTC	*cyaC* deletion
cyaC-OR	TTTCATTAGGTTAAAGGCATTCAC	*cyaC* deletion
lrbA-UF	GGCCATCCATTGTCATATCAAAG	*lrbA* deletion or LrbA C-terminal 3×Flag knock-in
lrbA-UR	AATATATACATACGTTACTGCACG	*lrbA* deletion or LrbA C-terminal 3×Flag knock-in
lrbA-InF	CTAACAGCACAACCCTCTGACCAT	*lrbA* deletion or LrbA C-terminal 3×Flag knock-in
lrbA-InR	AATAGCAAGTTGTTGTAGGACGAC	*lrbA* deletion or LrbA C-terminal 3×Flag knock-in
lrbA-OF	TGCATCTAGTCGACGAATGTAGTG	*lrbA* deletion or LrbA C-terminal 3×Flag knock-in
lrbA-OR	CCGTTATAGAGAGTTATTATTAGCC	*lrbA* deletion or LrbA C-terminal 3×Flag knock-in
lrbR-UF	AATAGTGGGTTACCACAATTTG	*lrbR* deletion
lrbR-UR	AGTCAGATATATCCTGATTAGTG	*lrbR* deletion
lrbR-InF	CGTATATTAGATAAATTCTCAGATC	*lrbR* deletion
lrbR-InR	GTGATTTCAAGAGTCACTAGCTGAG	*lrbR* deletion
lrbR-OF	ATAATCTACTAGCTCTGCATCGG	*lrbR* deletion
lrbR-OR	CTTATCAAGCTACTGAGCGACG	*lrbR* deletion

bpfA-UF	AAGCCTTAATACCAAGCGATAGAG	*bpfA* deletion
bpfA-DR	AATGGATCAACTATAACTCGCTGC	*bpfA* deletion
bpfA-INF	ACCAATGGCAATGTCTTTAGCATC	*bpfA* deletion
bpfA-INR	TGTAGGTAAAGCTGTCACTGCCAT	*bpfA* deletion
bpfA-OF	TTAGCACGCGTTGAGCTCGATAA	*bpfA* deletion
bpfA-OR	AAATGACATTATCAGCAACCCGAT	*bpfA* deletion
bpfD-UF	GCTTAGGTTATGTGCAATTTACTG	*bpfD* deletion
bpfD-DR	TGCCAGTCACTATCACTTGAATAT	*bpfD* deletion
bpfD-INF	TCAGTGGTTTATCAATCTCAATCT	*bpfD* deletion
bpfD-INR	CAAGATCCTTCAGTTCAGTGGTTC	*bpfD* deletion
bpfD-OF	CTCAACTCTGGTTAAACATTATGG	*bpfD* deletion
bpfD-OR	AAGGTAATACAGATTGACGGTGAT	*bpfD* deletion
bpfG-UF	GACAATTTACTCTGTCGATGACAA	*bpfG* deletion
bpfG-DR	CACATAACCTAAGCTTGCGACTAC	*bpfG* deletion
bpfG-INF	ATCTGAAGCAAGTACGTTAGATGA	*bpfG* deletion
bpfG-INR	ACGGTCAACACCTAATCTGTGGTT	*bpfG* deletion
bpfG-OF	CTTTTCGCTGGAGGAAGAGATCTT	*bpfG* deletion
bpfG-OR	TCTTGGTTAATTGTACGGATCAATG	*bpfG* deletion
3×Flag-F[a]	GGAGGTGGCGATTACAAGGATGAC	C-teminal 3×Flag knock-in
3×Flag-R	CGGGATCCTTTATCGTCATCATCTTTGTAGTC	C-teminal 3×Flag knock-in
lrbS-Flag-ConF	AATGATGAATTCTGCCTATTTAAGCCTATTAGTTTA	LrbS C-teminal 3×Flag knock-in
lrbS-Flag-ConR	AATATCCTGCAGTGATAGAGTCACCTTGAACCTTAG	LrbS C-teminal 3×Flag knock-in
lrbS-Flag-KinF	CGGGATCCTAAATCGCTCTAATGTTTTTGGTGATCTAT	LrbS C-teminal 3×Flag knock-in
lrbS-Flag-KinR[a]	CGCTGCTAAGTGGTTTTTTATCTCATCG	LrbS C-teminal 3×Flag knock-in
lrbS-Flag-SF	CTAGAACACCCCCAACAAGCATTT	LrbS C-teminal 3×Flag knock-in
lrbS-Flag-SR	TAGGCTGAGCATTGCTTTAGTATA	LrbS C-teminal 3×Flag knock-in
lrbS-Flag-OF	TATACAGCCAACGCACAACTTGAA	LrbS C-teminal 3×Flag knock-in
lrbS-Flag-OR	TTAAACACAATAGAATCCCACAGT	LrbS C-teminal 3×Flag knock-in
lrbR-Flag/HA-ConF	CATCTTCTGCAGTCTCCTCAGCTAGTGACTCTTGAA	LrbR C-teminal 3×Flag or 1×HA knock-in
lrbR-Flag/HA-ConR	TCAGTAGAATTCACTATATCGAGACCCATTAGCAAT	LrbR C-teminal 3×Flag or 1×HA knock-in
lrbR-Flag-KinF	CGGGATCCTAACGGAAGTAGACGAAAAGACATAA	LrbR C-teminal 3×Flag knock-in
lrbR-Flag-KinR[a]	TAGAGAGTTATTATTAGCCATTGAAA	LrbR C-teminal 3×Flag knock-in
lrbR-Flag/HA-SF	AATATTGAACCAAGATGTCAAGCT	LrbR C-teminal 3×Flag or 1×HA knock-in
lrbR-Flag/HA-SR	ACAATATTGAGCTTCACCATTGGA	LrbR C-teminal 3×Flag or 1×HA knock-in
lrbR-Flag/HA-OF	GGGAACAGCTATCTCTTGCATATA	LrbR C-teminal 3×Flag or 1×HA knock-in
lrbR-Flag/HA-OR	GCCGCAAGATGCCTTTCTACATAA	LrbR C-teminal 3×Flag or 1×HA knock-in

lrbR-HA-KinF[a]	GTCCCAGACTACGCTTAACGGAAGTAGACGAAAAGACATAA	LrbR C-teminal 1×HA knock-in
lrbR-HA-KinR[a]	GTCGTATGGGTAGCCACCTCCTTCTAGATTTTCTGGTGGCGCAATAAATCC	LrbR C-teminal 1×HA knock-in
lrbA-Flag-SF	CCGTATGCTATTAAGTAATAAAAC	LrbA C-teminal 3×Flag knock-in
lrbA-Flag-SR	TAGCTTTACTTCTCCTACAGTAGA	LrbA C-teminal 3×Flag knock-in
lrbA-Flag-OF	ATGCACTGAAACTCGTTAAAGATT	LrbA C-teminal 3×Flag knock-in
lrbA-Flag-OR	AGGTGAAACTTTATCTGAAATGTA	LrbA C-teminal 3×Flag knock-in
crp-Flag/HA-ConF	ACGTACGAATTCCGCTTGGGTTCGAGCTAAACAAGC	CRP C-teminal 3×Flag or1×HA knock-in
crp-Flag/HA-ConR	GATTAACTGCAGCGATTTGGCCGCGCTGAACTTTAA	CRP C-teminal 3×Flag or1×HA knock-in
crp-Flag-KinF	CGGGATCCTAAGTTAGTCTTCAGCTTGATTTAAGCCT	CRP C-teminal 3×Flag knock-in
crp-Flag-KinR[a]	ACGGGTACCGTATACCACTATGGTTTTG	CRP C-teminal 3×Flag knock-in
crp-Flag/HA-SF	GCAAATCAAGATCACTCGTCAAGA	CRP C-teminal 3×Flag or1×HA knock-in
crp-Flag/HA-SR	ATTATTGTTGGATACACCAGTGCG	CRP C-teminal 3×Flag or1×HA knock-in
crp-Flag/HA-OF	AGGTAAAGAGATGATCCTTTCTTA	CRP C-teminal 3×Flag or1×HA knock-in
crp-Flag/HA-OR	ATTGGGTTATCTTCAACGAGTAGC	CRP C-teminal 3×Flag or1×HA knock-in
crp-HA-KinF[a]	GTCCCAGACTACGCTTAAGTTAGTCTTCAGCTTGATTTAAGCCTG	CRP C-teminal 1×HA knock-in
crp-HA-KinR[a]	GTCGTATGGGTAGCCACCTCCACGGGTACCGTATACCACTATGGTTTTG	CRP C-teminal 1×HA knock-in
BpfA-Flag-SF	TGATGGTCCAGATAACGATACAGG	BpfA 3×Flag knock-in
BpfA-Flag-SR	ATCCATTACCAGCAACCATACGAA	BpfA 3×Flag knock-in
BpfA-Flag-OF	AAGCCTATGATGAGCAAGGTAATT	BpfA 3×Flag knock-in
BpfA- Flag-OR	TGCCACCATTTACAATATCCGCTT	BpfA 3×Flag knock-in
BpfD-Flag-SF	ATTACGATGAAGCACAGCATAACA	BpfD C-teminal 3×Flag knock-in
BpfD-Flag-SR	TATAGATAGGGTGGAGAGTCGTAA	BpfD C-teminal 3×Flag knock-in
BpfD-Flag-OF	TACATTGAATTATTATCGCTTGGTGC	BpfD C-teminal 3×Flag knock-in
BpfD-Flag-OR	GACCAAATTCTTCGCATAACTAGC	BpfD C-teminal 3×Flag knock-in
BpfG-HA-SF	TTAACGGTAAACAACTATGGCTTA	BpfG 1×HA knock-in
BpfG-HA-SR	CGTTGTTTAGATCGGACTCCATTT	BpfG 1×HA knock-in
BpfG-HA-OF	CGCTAGCAGATCGTTTTAACATCG	BpfG 1×HA knock-in
BpfG-HA-OR	CGAGAATACGTATGGTTTCATCTC	BpfG 1×HA knock-in
D*0099*-5F	TATTTAGAATTCATGTGATTGGTATTATTGCTGGCA	Sputcn32_0099 deletion
D*0099*-5R	AAAAAAAAGCTTAGTGCCAACTAAAGCCTTAAATTC	*Sputcn32_0099* deletion
D*0099*-3F	CAAAAAAAGCTTGCTGACGGTGATAATAATGACAAA	*Sputcn32_0099* deletion
D*0099*-3R	GGAGCGCTGCAGAGCAAACCATCGGCATCTAAATCG	*Sputcn32_0099* deletion

D*0099*-UF	CAACTGCAAGGTTTGCAGCAAGAT	*Sputcn32_0099* deletion
D*0099*-DR	GTTATTTGCGATTTGAGTCAGGAA	*Sputcn32_0099* deletion
D*0099*-InF	AGGATAGCCGCATTCACAATTTGG	*Sputcn32_0099* deletion
D*0099*-InR	CAGGATATTCTGTCGGATCGATTC	*Sputcn32_0099* deletion
D*0099*-OF	TGAGGCTCTGAACTGATTAGATAT	*Sputcn32_0099* deletion
D*0099*-OR	TTCGTGTCGATAACCATACTAGGC	*Sputcn32_0099* deletion
D*0327*-5F	GTATTCGAATTCAACCTATTTATCCTTCGACGCGAT	*Sputcn32_0327* deletion
D*0327*-5R	GAAAAAGGATCCCACATCGCCTAGCATCAAACTTGG	*Sputcn32_0327* deletion
D*0327*-3F	AAAGCTGGATCCAAAACCTTGGATAAGTGCTAGCGG	*Sputcn32_0327* deletion
D*0327*-3R	TTTATCCTGCAGTTGATGGTAAGCAACTAATTCAGT	*Sputcn32_0327* deletion
D*0327*-UF	GCACTGCAAATGATGAGATAGCTT	*Sputcn32_0327* deletion
D*0327*-DR	ATACCTATGGAATGGGATGTGACT	*Sputcn32_0327* deletion
D*0327*-InF	TTAAGTGAAAACGGTTGGGTAAGC	*Sputcn32_0327* deletion
D*0327*-InR	ACTACAAGCTCTTAACCGTCGCAA	*Sputcn32_0327* deletion
D*0327*-OF	GTTACACCACTCACATTAAATACC	*Sputcn32_0327* deletion
D*0327*-OR	ACAATAACGAGTTGACAAATTTGG	*Sputcn32_0327* deletion
D*0384*-5F	GGAAATGAATTCACCTGTGAAATGCTCCTTGTTATG	*Sputcn32_0384* deletion
D*0384*-5R	TCGTAAGGATCCCAAGTCCTGCTGAATTTCTGAGTA	*Sputcn32_0384* deletion
D*0384*-3F	CGAAATGGATCCCAAGTGGTATTGAATCATCCATGA	*Sputcn32_0384* deletion
D*0384*-3R	AAAGTTCTGCAGCAAACAACTACGACCACATTGATC	*Sputcn32_0384* deletion
D*0384*-UF	GCACAATTGAGATACATTTATGCA	*Sputcn32_0384* deletion
D*0384*-DR	TGGGTTCCTAAGTACAATCCTGAC	*Sputcn32_0384* deletion
D*0384*-InF	GTCAGAGAAGAGTCGAAATTATTA	*Sputcn32_0384* deletion
D*0384*-InR	CAGATACTAAACGACTATTGAGAT	*Sputcn32_0384* deletion
D*0384*-OF	CATTATCAAGATGGTGGTCAAAGG	*Sputcn32_0384* deletion
D*0384*-OR	CCCAAGCACTGTACACACTAGATC	*Sputcn32_0384* deletion
D*0414*-5F	GCGTTTCTGCAGACAACAAGAATTAGTCCACAACGT	*Sputcn32_0414* deletion
D*0414*-5R	TCGCCAGGATCCTCAGTGAACAAGCTTGAAACGTAA	*Sputcn32_0414* deletion
D*0414*-3F	ACTGCCGGATCCCGCATAACCACTAAAATTATCGCT	*Sputcn32_0414* deletion
D*0414*-3R	CAACATGAATTCTACCTGCAAGTATGATCAAATCAG	*Sputcn32_0414* deletion
D*0414*-UF	CTTGAGTTTATTGATGATGACGAA	*Sputcn32_0414* deletion
D*0414*-DR	GAAAACACGTTATATCCTCTCATT	*Sputcn32_0414* deletion
D*0414*-InF	CAATCATAGACTCAAGTTCGCGAT	*Sputcn32_0414* deletion
D*0414*-InR	GGATCCACATCGAAATGCTAGATT	*Sputcn32_0414* deletion
D*0414*-OF	GCGAACTGAAAATCTCTGATACTG	*Sputcn32_0414* deletion
D*0414*-OR	TACCATCTACGCCTTCAGGATTCA	*Sputcn32_0414* deletion
D*0555*-5F	GCGTTTCTGCAGACAACAAGAATTAGTCCACAACGT	*Sputcn32_0555* deletion
D*0555*-5R	TCGCCAGGATCCTCAGTGAACAAGCTTGAAACGTAA	*Sputcn32_0555* deletion
D*0555*-3F	ACTGCCGGATCCCGCATAACCACTAAAATTATCGCT	*Sputcn32_0555* deletion
D*0555*-3R	CAACATGAATTCTACCTGCAAGTATGATCAAATCAG	*Sputcn32_0555* deletion
D*0555*-UF	CTTGAGTTTATTGATGATGACGAA	*Sputcn32_0555* deletion
D*0555*-DR	GAAAACACGTTATATCCTCTCATT	*Sputcn32_0555* deletion

D*0555*-InF	CAATCATAGACTCAAGTTCGCGAT	*Sputcn32_0555* deletion
D*0555*-InR	GGATCCACATCGAAATGCTAGATT	*Sputcn32_0555* deletion
D*0555*-OF	GCGAACTGAAAATCTCTGATACTG	*Sputcn32_0555* deletion
D*0555*-OR	TACCATCTACGCCTTCAGGATTCA	*Sputcn32_0555* deletion
D*0601*-5F	CTTCTAGAATTCGCAGGGTTACATATTGACACAGGT	*Sputcn32_0601* deletion
D*0601*-5R	TTCACGGGATCCACTAGGCCAATGGATAATAGGTTG	*Sputcn32_0601* deletion
D*0601*-3F	GCCCCAGGATCCAAGTAAATGGTTCGGTGGATTGAT	*Sputcn32_0601* deletion
D*0601*-3R	CTTAAGCTGCAGCTGGAAGCTGTAATACATGTCATC	*Sputcn32_0601* deletion
D*0601*-UF	AGTAAGCATAGAAGATAGAGTCGA	*Sputcn32_0601* deletion
D*0601*-DR	GAACTGATGCAGACGATGGATACT	*Sputcn32_0601* deletion
D*0601*-InF	GTGTTTGAGCGGATTAATGAAGAT	*Sputcn32_0601* deletion
D*0601*-InR	GGATCAAGGTCATATCGAGTTTTA	*Sputcn32_0601* deletion
D*0601*-OF	ATCAGTGCAAGGTTCTAGCTTCTA	*Sputcn32_0601* deletion
D*0601*-OR	CCTTCAATGCCAATAACGTGACCG	*Sputcn32_0601* deletion
D*1039*-5F	AACCCAGAATTCATTACCAATTAGCTCCTGGAGACT	*Sputcn32_1039* deletion
D*1039*-5R	GATACCGGATCCGTCGATAATATCACGGAGCAATAA	*Sputcn32_1039* deletion
D*1039*-3F	ACGATAGGATCCATCTAAGCGGTCTATGGTCTTACG	*Sputcn32_1039* deletion
D*1039*-3R	CATCAACTGCAGGATGGCATATTAATGGCGTCAATT	*Sputcn32_1039* deletion
D*1039*-UF	GGTTGATGAGAGATCCAAGCGTTA	*Sputcn32_1039* deletion
D*1039*-DR	ATTAACACTGAGTACCGATATGGA	*Sputcn32_1039* deletion
D*1039*-InF	ATGCCCATAAGTGTCATTGATGGT	*Sputcn32_1039* deletion
D*1039*-InR	AGGTAGACTCAGTCGACTATGGCT	*Sputcn32_1039* deletion
D*1039*-OF	GCGATAAGCCTATGTCTCTTTCTC	*Sputcn32_1039* deletion
D*1039*-OR	CCGAAGCGAGTGATATACCAAATG	*Sputcn32_1235* deletion
D*1235*-5F	ATTTGCGAATTCTTGCGTTTCTCACTTAGCAATCTC	*Sputcn32_1235* deletion
D*1235*-5R	TTTACCGGATCCAACATTCTTCTCATACTCTGCTAA	*Sputcn32_1235* deletion
D*1235*-3F	GCCAGCGGATCCATGTCGTAGCCAGCATGTCGTAAG	*Sputcn32_1235* deletion
D*1235*-3R	TTAACTCTGCAGCTGCACTACCCATGACGAGCAATA	*Sputcn32_1235* deletion
D*1235*-UF	GAGTGGTGATGGAGACTATAAGGC	*Sputcn32_1235* deletion
D*1235*-DR	GCACAGAAGTGAGTAAACTCGACG	*Sputcn32_1235* deletion
D*1235*-InF	TTCAGCTAGAAATCAGTCATGTGC	*Sputcn32_1235* deletion
D*1235*-InR	ACATATTAGTGTATTCACGCTCTT	*Sputcn32_1235* deletion
D*1235*-OF	AGACACCTATGAACAAATGGTTAA	*Sputcn32_1235* deletion
D*1235*-OR	GTCTCAAGTTGGTGTCGATAACTC	*Sputcn32_1235* deletion
D*1253*-5F	CTTAAAGAATTCCGGTTGTACTTACCCTGATATTAA	*Sputcn32_1253* deletion
D*1253*-5R	CATCCAGGATCCAGGGGTACTCATAATAGTGATACCA	*Sputcn32_1253* deletion
D*1253*-3F	GCGCCAGGATCCGCGTAAGGCCTTAACTCGAAAATT	*Sputcn32_1253* deletion
D*1253*-3R	GTCGCACTGCAGACCAATACACGCAACTTACCTAGC	*Sputcn32_1253* deletion
D*1253*-UF	ACTGGCATTCTGTTGCTCAAGTTT	*Sputcn32_1253* deletion
D*1253*-DR	TAAGTGGCAGAACAGTAATTCAAG	*Sputcn32_1253* deletion
D*1253*-InF	AGGTCTTATTGCCTTCGGATCCTA	*Sputcn32_1253* deletion
D*1253*-InR	GGCCAATGCGGTGGATATAATCTT	*Sputcn32_1253* deletion

D*1253*-OF	ATGTGGATTGGTGTTGCCGTGATA	*Sputcn32_1253* deletion
D*1253*-OR	TATTGCCAACGGTAAATTGACTAC	*Sputcn32_1253* deletion
D*1741*-5F	GCATTAGAATTCTCAATGAATTACCACTTAGTCAGC	*Sputcn32_1741* deletion
D*1741*-5R	GTGATGGGATCCCCAATAAGTAACTAGGCGTGTTGA	*Sputcn32_1741* deletion
D*1741*-3F	TTGTAAGGATCCTAATTCTCGTGACATGCAGTGACC	*Sputcn32_1741* deletion
D*1741*-3R	CTGTTTCTGCAGTAAGTTATCAAAATTCATTCCCGC	*Sputcn32_1741* deletion
D*1741*-UF	AACTACGATTTGTGGTACAAGCCG	*Sputcn32_1741* deletion
D*1741*-DR	TAAGAACACTGACTAGGATGAAAT	*Sputcn32_1741* deletion
D*1741*-InF	GCGGTACAGTTTAATTCCATCACG	*Sputcn32_1741* deletion
D*1741*-InR	GAATTGGACGTACGAGAAATGCGC	*Sputcn32_1741* deletion
D*1741*-OF	ATCAAGATGCGGGTTTAGATTTAC	*Sputcn32_1741* deletion
D*1741*-OR	CATGCATCATCACCACTTCATCTG	*Sputcn32_1741* deletion
D*1800*-5F	GTACGCCTGCAGTACCCGTTGATACAGTTAATTCTG	*Sputcn32_1800* deletion
D*1800*-5R	CCGTTTACTAGTACCATCATCACTCAACAGTAGGAC	*Sputcn32_1800* deletion
D*1800*-3F	GAAACAACTAGTGAGGCTCAGTTGAAGTTTGTGATG	*Sputcn32_1800* deletion
D*1800*-3R	AGGCGAGAATTCTGATTGTGAACTGACGAATAATCG	*Sputcn32_1800* deletion
D*1800*-UF	AGATCACGATGTTAGCTTAGATTG	*Sputcn32_1800* deletion
D*1800*-DR	GATTATCTGTGATACCAATACCTT	*Sputcn32_1800* deletion
D*1800*-InF	TACCTTGATCTTACTGTAACGGTA	*Sputcn32_1800* deletion
D*1800*-InR	TTCCAATACTAACCGAACTGTAAA	*Sputcn32_1800* deletion
D*1800*-OF	GAGCAACTTGGTAAATCTTGGGAA	*Sputcn32_1800* deletion
D*1800*-OR	CTGAACACGATATTTCACTAATGT	*Sputcn32_1800* deletion
D*1851*-5F	GCTCAGCTGCAGGCTCAACACCCATCAAATTGGCTA	*Sputcn32_1851* deletion
D*1851*-5R	CAGCCCGGATCCCAAGCGATCACTAGCAGAGATCAA	*Sputcn32_1851* deletion
D*1851*-3F	ATTTGAGGATCCTTAGATAGATTGAGCTATTTGTGCGTT	*Sputcn32_1851* deletion
D*1851*-3R	TCAGGGGAATTCGAGCCATTTACAGTAAACTATCAA	*Sputcn32_1851* deletion
D*1851*-UF	GTTGCCATAGACATACCAATAGCG	*Sputcn32_1851* deletion
D*1851*-DR	CCTTACATCTGACAATGCACATCC	*Sputcn32_1851* deletion
D*1851*-InF	AATCACTCTGATACCTATTCGCCT	*Sputcn32_1851* deletion
D*1851*-InR	TAGATAAACTAGTGGTGATCAGTG	*Sputcn32_1851* deletion
D*1851*-OF	CAATACACTCATCCTCACGAATAA	*Sputcn32_1851* deletion
D*1851*-OR	ACTGTTAATCCGTATGTCACTGGT	*Sputcn32_1851* deletion
D*1917*-5F	CTGATTGAATTCTACCCATTGAGCAAACAGACATCA	*Sputcn32_1917* deletion
D*1917*-5R	ATGGGCGGATCCAAACCATAGCGTTAACAATGGGAA	*Sputcn32_1917* deletion
D*1917*-3F	GAGCAAGGATCCGAGTGCTAAGACTAACCCAAGTAA	*Sputcn32_1917* deletion
D*1917*-3R	CCACGTCTGCAGACCATCGAGGATCACAATTTGTTT	*Sputcn32_1917* deletion
D*1917*-UF	TTGTCTTGAATGCCGTGCCAGA	*Sputcn32_1917* deletion
D*1917*-DR	TCATCACCAGATAGCACTATGGCT	*Sputcn32_1917* deletion
D*1917*-InF	ATCATCAGCTCCTTGAATCCATGG	*Sputcn32_1917* deletion
D*1917*-InR	CACTCTTACGGATCAATTCAGCAC	*Sputcn32_1917* deletion
D*1917*-OF	GTTGTTGTGATCATGGTTTACTTG	*Sputcn32_1917* deletion
D*1917*-OR	TTGCTGCCGATTTCAGATACGAAC	*Sputcn32_1917* deletion

D*2096*-5F	AAATCCGAATTCCCAATCATTAGCCAGAGTCATATC	*Sputcn32_1917* deletion
D*2096*-5R	GGCGCAGGATCCATTACAGTATCGATCGGTCTTGCC	*Sputcn32_2096* deletion
D*2096*-3F	AGTCCCGGATCCCGCAAAATATTCATACCAAACCGTAT	*Sputcn32_2096* deletion
D*2096*-3R	TGAAACCTGCAGATGCTGATATTGAGGTGAAGTGGT	*Sputcn32_2096* deletion
D*2096*-UF	GAAGATGTGGCGTTGTGTATTGTG	*Sputcn32_2096* deletion
D*2096*-DR	TAATGAATGTGAAGACTGAGGTGT	*Sputcn32_2096* deletion
D*2096*-InF	GACATAAGCTAATACTTTATCGCC	*Sputcn32_2096* deletion
D*2096*-InR	TCTTGACTTAAACCGTGCGATTGA	*Sputcn32_2096* deletion
D*2096*-OF	AATAGAGTTGTTACTTGATGGTGT	*Sputcn32_2096* deletion
D*2096*-OR	ATACGGTTATGGATGTGCTGAAAC	*Sputcn32_2096* deletion
D*2106*-5F	GGGCCAGAATTCTCAGTAACGAGCGTAATTTCTATG	*Sputcn32_2106* deletion
D*2106*-5R	ACTGAGGGATCCTGTTTTAAGGTTAATCATTGGCCA	*Sputcn32_2106* deletion
D*2106*-3F	CGTTTTGGATCCTCCTAACCTCATCACTAAACTCGG	*Sputcn32_2106* deletion
D*2106*-3R	CACAGCCTGCAGCAAATACGGCTTGTACGTCTTCTG	*Sputcn32_2106* deletion
D*2106*-UF	TGGCACTGATTGTCTCCTAAAACG	*Sputcn32_2106* deletion
D*2106*-DR	CACTAGACTCCTTATGCCATGCTA	*Sputcn32_2106* deletion
D*2106*-InF	GTCGATCCACAAACCTTAGCCATT	*Sputcn32_2106* deletion
D*2106*-InR	ATTCATCATTAATTCCACAGACAG	*Sputcn32_2106* deletion
D*2106*-OF	GCGTTGGCAATACTGGTTATACCG	*Sputcn32_2106* deletion
D*2106*-OR	CGAGGATACGCTCTGAAATCGCTT	*Sputcn32_2106* deletion
D*2456*-5F	CAAGTGGAATTCCCGAATCAACTAGAGAATTAGTCA	*Sputcn32_2456* deletion
D*2456*-5R	GACTTTGGATCCCAAGAGAATGTTAGGCATAGTATTGT	*Sputcn32_2456* deletion
D*2456*-3F	GCGATAGGATCCAAGGAATCCGAGTTTACTCTACTT	*Sputcn32_2456* deletion
D*2456*-3R	TAAATTCTGCAGGTACTGTTAGCATTTTGAGGTGAT	*Sputcn32_2456* deletion
D*2456*-UF	ATGGCATCATCGGATAATATTAAG	*Sputcn32_2456* deletion
D*2456*-DR	ACTTCTAATGGCTTGAATTTAACG	*Sputcn32_2456* deletion
D*2456*-InF	AGCAAGTCAGAATGGTCTATATCA	*Sputcn32_2456* deletion
D*2456*-InR	CATGATCTATAATGTTGCTCTTAG	*Sputcn32_2456* deletion
D*2456*-OF	TTTATCCTTAGTCTCGACGCCAAG	*Sputcn32_2456* deletion
D*2456*-OR	AATGCGCGCTAATGAATGAGGCTC	*Sputcn32_2456* deletion
D*2671*-5F	TGCCATGAATTCTCCCTATAATAACTATGTAATGGG	*Sputcn32_2671* deletion
D*2671*-5R	GACTTTGGATCCTACCACATCTTCAGTTACTAAATCT	*Sputcn32_2671* deletion
D*2671*-3F	ATTAACGGATCCATTCCATTGCGTTATTACTAAGACA	*Sputcn32_2671* deletion
D*2671*-3R	GATAAACTGCAGGATGATCGCTGCACTGTGTTAGAT	*Sputcn32_2671* deletion
D*2671*-UF	TTGGCCACTTTACGACCACAGCAA	*Sputcn32_2671* deletion
D*2671*-DR	ATAAGAGTGAGTACAATAGCTAACA	*Sputcn32_2671* deletion
D*2671*-InF	CTAATGCTCATTTAACCTATCAAGC	*Sputcn32_2671* deletion
D*2671*-InR	ACATCACAATACTCAGTGGTACTT	*Sputcn32_2671* deletion
D*2671*-OF	GAGTGGTACCTTGAGCCTTATTAC	*Sputcn32_2671* deletion
D*2671*-OR	ACCTGATAATAGTTCAAATAAAGGG	*Sputcn32_2671* deletion
D*2800*-5F	TAACCAGAATTCACCAGCAAGATCGACTAAAGGGAT	*Sputcn32_2800* deletion
D*2800*-5R	CATTATGGATCCGCGACGTAATTTGCCACTCAATCT	*Sputcn32_2800* deletion

D*2800*-3F	ACTCAGGGATCCCCCAGTAAGCTTATCTTTACCCAT	*Sputcn32_2800* deletion
D*2800*-3R	GCCCTCCTGCAGGTGTTACCTACCTTGCCAGAAGTG	*Sputcn32_2800* deletion
D*2800*-UF	ATTATTGATGTGCGTACTCAGTTT	*Sputcn32_2800* deletion
D*2800*-DR	ACTATGTTGTCAACTCGATCAGCA	*Sputcn32_2800* deletion
D*2800*-InF	ACTAACCTATCCCTAATCTCGAGC	*Sputcn32_2800* deletion
D*2800*-InR	TTCTACACGGGATCTAGACAGTGA	*Sputcn32_2800* deletion
D*2800*-OF	CGATGAATACTACGAACGCTATCA	*Sputcn32_2800* deletion
D*2800*-OR	TCTCATCACAGCCAACTGATATAC	*Sputcn32_2800* deletion
D*2830*-5F	TACCAGGAATTCCAACCACTAAGTTCAATTGGCTCG	*Sputcn32_2830* deletion
D*2830*-5R	TAGGGTGGATCCTTGTCTCGCTAAGTTGTCATCAAG	*Sputcn32_2830* deletion
D*2830*-3F	ACAATCGGATCCACAAGTATCGACAGCAGAGCATAG	*Sputcn32_2830* deletion
D*2830*-3R	AGGTGACTGCAGTGAGTTGACCTTAGACATTCATTG	*Sputcn32_2830* deletion
D*2830*-UF	CAAGCTTTATGACGGGTCGCTTTA	*Sputcn32_2830* deletion
D*2830*-DR	AAGTTAGGTCTACGTCGTTAATCT	*Sputcn32_2830* deletion
D*2830*-InF	CGTGTACACAAACGTAGCTTTGCA	*Sputcn32_2830* deletion
D*2830*-InR	TGACGGATTAGATCTTCGGCACTA	*Sputcn32_2830* deletion
D*2830*-OF	AAACTCAGCAATCGCGTTAATAAC	*Sputcn32_2830* deletion
D*2830*-OR	TGAGCTTAATTTCATCAGTCTTGA	*Sputcn32_2830* deletion
D*3018*-5F	CTTTGTGAATTCGGAACGTCTTCATGGCATGATCTC	*Sputcn32_3018* deletion
D*3018*-5R	TTAAGAGGATCCTCTATCTTTGTTGATACGATACTG	*Sputcn32_3018* deletion
D*3018*-3F	AAAAATGGATCCTCGTTCTGCTCCTGCACTAACAAA	*Sputcn32_3018* deletion
D*3018*-3R	CGGGTCCTGCAGGGCTATCCATAATGATTCGATTGA	*Sputcn32_3018* deletion
D*3018*-UF	GAGTCTATTCGTGAAGTCTCTGAA	*Sputcn32_3018* deletion
D*3018*-DR	GGAGATCTGCGTGAATAATGATGA	*Sputcn32_3018* deletion
D*3018*-InF	TTTAGCAGAATCAACTCGTTCGAG	*Sputcn32_3018* deletion
D*3018*-InR	TTGCCCTATTAGCTTAAACGAGTA	*Sputcn32_3018* deletion
D*3018*-OF	CAAGCCCTTAATGATATTGCCGAA	*Sputcn32_3018* deletion
D*3018*-OR	GCATCAATAATGGTTTCATTGAAA	*Sputcn32_3018* deletion
D*3085*-5F	CCTTATGAATTCCCATTCTGTTGATGCTGACTTTGG	*Sputcn32_3085* deletion
D*3085*-5R	CATCACGGATCCAGTGATGCATCTTAAATTGCTTAT	*Sputcn32_3085* deletion
D*3085*-3F	TGCATCGGATCCGGTATCTAACACATTGAGAGCTCG	*Sputcn32_3085* deletion
D*3085*-3R	GGTGTGCTGCAGCCTGGGTATCTTGAGAGCAGTGAT	*Sputcn32_3085* deletion
D*3085*-UF	AAACAATGGCGCCTACTCGTATTT	*Sputcn32_3085* deletion
D*3085*-DR	GAACATGCAGAGCGGGTACTGAAC	*Sputcn32_3085* deletion
D*3085*-InF	CGGAATCTAACAGATCCTCTAAGC	*Sputcn32_3085* deletion
D*3085*-InR	ATTATCTATCGCGCAGAATACCTT	*Sputcn32_3085* deletion
D*3085*-OF	GATACTTATGCCCGATATTGTGAT	*Sputcn32_3085* deletion
D*3085*-OR	GCGTATTACCTGTGATGCAAGTCA	*Sputcn32_3085* deletion
D*3168*-5F	GAAACCCTGCAGGATTTAGTCTGGCAGCCATTTCGC	*Sputcn32_3168* deletion
D*3168*-5R	GCTGAGGGATCCCGATTACGCGAAATTGTTGCTTGT	*Sputcn32_3168* deletion
D*3168*-3F	TCCAGTGGATCCCGTTGTAATCAAGCTCTGCATACT	*Sputcn32_3168* deletion
D*3168*-3R	AACACCGAATTCTGGAGAGGCTTTCGTTGAGATTAA	*Sputcn32_3168* deletion

D*3168*-UF	TCAACTATCTGGTATTTACTGACT	*Sputcn32_3168* deletion
D*3168*-DR	TATTCAAGATTGGATTAATGCCCA	*Sputcn32_3168* deletion
D*3168*-InF	GACCATAGCTATCATTAATACGTT	*Sputcn32_3168* deletion
D*3168*-InR	TGGTATTTAGTCAATCTCTATCGT	*Sputcn32_3168* deletion
D*3168*-OF	AATACATGAATTTGAACTCAGCCA	*Sputcn32_3168* deletion
D*3168*-OR	ACTTATTCAACAGTTAACGGAGGC	*Sputcn32_3168* deletion
D*3269*-5F	TAAAGCGAATTCTAGCGTTAACCGTACAAGCACTCG	*Sputcn32_3269* deletion
D*3269*-5R	GACCGCGGATCCTTGATAACGGATTTTAAGTGTCGC	*Sputcn32_3269* deletion
D*3269*-3F	AAAGCCGGATCCAAACAACTTGCCTCACTTGCTATC	*Sputcn32_3269* deletion
D*3269*-3R	AGAGCACTGCAGAAGCTAAAGTGTTGATCGTCGAGA	*Sputcn32_3269* deletion
D*3269*-UF	CAAGTGCTATCGTGATACTCAACA	*Sputcn32_3269* deletion
D*3269*-DR	TACAAATCGCTAAGGATGCTTTAG	*Sputcn32_3269* deletion
D*3269*-InF	GATAACATCACTGTCACGACCAAT	*Sputcn32_3269* deletion
D*3269*-InR	CTCATCAATCACGATATAGCCTTG	*Sputcn32_3269* deletion
D*3269*-OF	CATGGTTTACCAGTTGGCTTTCAG	*Sputcn32_3269* deletion
D*3269*-OR	GATTTCAGATATCTACAGCACCAT	*Sputcn32_3269* deletion
D*3306*-5F	ATGACAGAATTCATATCTGTACTCATCTGTGACGAT	*Sputcn32_3306* deletion
D*3306*-5R	GGTTTGGGATCCTACCATATCAATGAGCCAGTGTAG	*Sputcn32_3306* deletion
D*3306*-3F	ATAGGCGGATCCAAGCATATTAGTAGTAGCTCAAGT	*Sputcn32_3306* deletion
D*3306*-3R	TTGTCGCTGCAGGGCAATATTCTTATCGATCGGAAA	*Sputcn32_3306* deletion
D*3306*-UF	CTGGTAACGTGCTAATTGGTGCAT	*Sputcn32_3306* deletion
D*3306*-DR	TAGTACACTACAGTTACGACCTTT	*Sputcn32_3306* deletion
D*3306*-InF	TTGACTTATATGCTCGCTATAACG	*Sputcn32_3306* deletion
D*3306*-InR	CTCATACACTATGGTCGACTCAGC	*Sputcn32_3306* deletion
D*3306*-OF	AATAAGGATCACCAGTCAGCATGA	*Sputcn32_3306* deletion
D*3306*-OR	GGGCACGGTTAGCTTAGGTAAGAA	*Sputcn32_3306* deletion
D*3390*-5F	CTTTGTGAATTCAACACCATAAGTTAATAACACGAC	*Sputcn32_3390* deletion
D*3390*-5R	GAGCGTGGATCCGCAGATATGGCAATGTATGATGTT	*Sputcn32_3390* deletion
D*3390*-3F	TTCTGCGGATCCTTGAAGTTGCTGAATTAACGCTGT	*Sputcn32_3390* deletion
D*3390*-3R	AAAGACCTGCAGACTGAGCTTACATTTACCGCAAGT	*Sputcn32_3390* deletion
D*3390*-UF	CTCAACACCAATTCCATTACGAAT	*Sputcn32_3390* deletion
D*3390*-DR	CGACTAAGACACCTGATTCATTAG	*Sputcn32_3390* deletion
D*3390*-InF	TCGCTACATGTATCAGTAAGCTAT	*Sputcn32_3390* deletion
D*3390*-InR	TACAAGTCTTCGAAGATCACGTTA	*Sputcn32_3390* deletion
D*3390*-OF	ACTGACTGTAGTAAATGCCACCAT	*Sputcn32_3390* deletion
D*3390*-OR	TTCAGATTTAGTGGCATGATTGGT	*Sputcn32_3390* deletion
D*3648*-5F	CCGGACGAATTCATTGGCAGCTTTAACGCTACTATG	*Sputcn32_3648* deletion
D*3648*-5R	GCCAGCAAGCTTACTGTCCATAAATAGACTCGTGAT	*Sputcn32_3648* deletion
D*3648*-3F	AAACAAAAGCTTCTGATGCAATAAACCGAGTCGCAC	*Sputcn32_3648* deletion
D*3648*-3R	TGCAATCTGCAGGGCATTACACTATGGTCAGTCAAT	*Sputcn32_3648* deletion
D*3648*-UF	TTGCGTCCATTCACCTACAAACTT	*Sputcn32_3648* deletion
D*3648*-DR	AAATTTATCGCTGATATCGAATCC	*Sputcn32_3648* deletion

D*3648*-InF	TTCACTCACCGAATGATCCTACCA	*Sputcn32_3648* deletion
D*3648*-InR	CCAGCCAATAAGTAGGTGTTGTCA	*Sputcn32_3648* deletion
D*3648*-OF	CAAATCGAATTTATGAATCCAGCA	*Sputcn32_3648* deletion
D*3648*-OR	GCGCCGTACTGAGCAATTCAAGCC	*Sputcn32_3648* deletion
D*3856*-5F	AAGAGACTGCAGCTCACAATTACAACAGTCGTGAGC	*Sputcn32_3856* deletion
D*3856*-5R	CGTTTTGGATCCATTCAGCGTAACTTATAGCCTTGG	*Sputcn32_3856* deletion
D*3856*-3F	TTTGGCGGATCCGGCTAATATTGCTTGAATGCTGGA	*Sputcn32_3856* deletion
D*3856*-3R	AAGCAGGAATTCCAGTCGATTAAATGCCATAACTGC	*Sputcn32_3856* deletion
D*3856*-UF	TCAAGTGTAAAACTGATATTTGCA	*Sputcn32_3856* deletion
D*3856*-DR	TTCTCATAACCTTAGCCTATGTCG	*Sputcn32_3856* deletion
D*3856*-InF	ACCGTTAGTCCTAATTCATGTGAC	*Sputcn32_3856* deletion
D*3856*-InR	TTAGAATGGCGGTAAATTTATCGG	*Sputcn32_3856* deletion
D*3856*-OF	AGTCCAACCAAGATTTACGACATA	*Sputcn32_3856* deletion
D*3856*-OR	GTGAAGCGTTAGTTAAATCGAGAT	*Sputcn32_3856* deletion
D*3917*-5F	TGAACACTGCAGCGATTTAAATACCTCAGAGCAGCA	*Sputcn32_3917* deletion
D*3917*-5R	ATAGAGGGATCCTCAGCACAGCAACAACAGATATTT	*Sputcn32_3917* deletion
D*3917*-3F	CACCTCGGATCCTTGCTCTATACGCTCAATATCCTG	*Sputcn32_3917* deletion
D*3917*-3R	TATGGCGAATTCTGTTCGCCATTTAAAGTGGTACGC	*Sputcn32_3917* deletion
D*3917*-UF	TGAGTACCCATTGCCAATTGAAAG	*Sputcn32_3917* deletion
D*3917*-DR	AGAGTGATCAAATACGCAGTGAGC	*Sputcn32_3917* deletion
D*3917*-InF	CGGATACTGATTTAAGCAGCTTAA	*Sputcn32_3917* deletion
D*3917*-InR	AAATTCTCTCTGCAGAAGTGTTAT	*Sputcn32_3917* deletion
D*3917*-OF	TGGTTGTCTGTTACACTCTAGCAA	*Sputcn32_3917* deletion
D*3917*-OR	CGCTCAACGGATAAAAGGTACTCC	*Sputcn32_3917* deletion
D*0814*-5F	GTGGAAGAATTCAGGTATGTCAACACTTATGGCCAA	*Sputcn32_0814* deletion
D*0814*-5R	TGCAAGGGATCCCAAGATAGGCAGTAGCAAGATGAC	*Sputcn32_0814* deletion
D*0814*-3F	TTAGTCGGATCCGAGCAGATCTAACCTAGTCGTGAT	*Sputcn32_0814* deletion
D*0814*-3R	AAAAACCTGCAGTGGCGGTTAATGCGGCAATCAATC	*Sputcn32_0814* deletion
D*0814*-UF	ATGCAGTCTATTCAACCCTAATGT	*Sputcn32_0814* deletion
D*0814*-DR	GAGAATAACGTTGAAACTGGTCAC	*Sputcn32_0814* deletion
D*0814*-InF	GCTAAGTTAAGTGAGTTTATGAGG	*Sputcn32_0814* deletion
D*0814*-InR	CTCTAGGCACTTCTAATAACATCA	*Sputcn32_0814* deletion
D*0814*-OF	GCGGTAAGTATCTGCATTGGTGGT	*Sputcn32_0814* deletion
D*0814*-OR	AACATCATTCCTTGTCTATGGCAA	*Sputcn32_0814* deletion
D*3141*-5F	GCTACCGAATTCTTGCCTCCACTTATGCTTGCCAAC	*Sputcn32_3141* deletion
D*3141*-5R	GCGATTGGATCCCGAGATACCTATTTAGAGCATTGA	*Sputcn32_3141* deletion
D*3141*-3F	ATCATCGGATCCAACGACTAATATTGTTGCCTTATC	*Sputcn32_3141* deletion
D*3141*-3R	CTCCCCCTGCAGGTTATCGCTATGACCGCAAATGCC	*Sputcn32_3141* deletion
D*3141*-UF	CGTTAACGGTGGATCTGGAAACTA	*Sputcn32_3141* deletion
D*3141*-DR	TTTGATGAAGCGGTGGACTTGATT	*Sputcn32_3141* deletion
D*3141*-InF	TCGCAATGACTTCTGGCAGGATTT	*Sputcn32_3141* deletion
D*3141*-InR	GCTTACATGTGGTACGTATGAGTC	*Sputcn32_3141* deletion

D*3141*-OF	ATTCCGTTACAATCTAGCCTCTCA	*Sputcn32_3141* deletion
D*3141*-OR	TTAGATAAACTGTCTCAGCAAAGT	*Sputcn32_3141* deletion
D*1344*-5F	GCTTAAGAATTCTCGCTAAGATTACAAGTGTTTGCT	*Sputcn32_1344* deletion
D*1344*-5R	CCCAACGGATCCGATAAGACTTCGACTAGTTGACAT	*Sputcn32_1344* deletion
D*1344*-3F	ATGGTAGGATCCCGAAATGCGATCAATGAGTTTGAG	*Sputcn32_1344* deletion
D*1344*-3R	ATGTTGCTGCAGCAGACTAATGATGGGCTACAACTA	*Sputcn32_1344* deletion
D*1344*-UF	GCTACAGTTAGTGCTCAATGAACA	*Sputcn32_1344* deletion
D*1344*-DR	TTGAAGAAGGCTCAGTGATTTCTA	*Sputcn32_1344* deletion
D*1344*-InF	TTTCAGTTACAAGATGATCAAGGC	*Sputcn32_1344* deletion
D*1344*-InR	CGCAAAGGTGAGTAAAGATCTTGC	*Sputcn32_1344* deletion
D*1344*-OF	TCTTAATGACAGATTGTGCCGATG	*Sputcn32_1344* deletion
D*1344*-OR	GCCTTAGATTCACCTGGATTATTG	*Sputcn32_1344* deletion
D*1988*-5F	CAGATAGGATCCTTTAGCACACTATGCGGATAGTCT	*Sputcn32_1988* deletion
D*1988*-5R	AATTGGTCTAGATCACTGTATCGTCGAATTCCATAA	*Sputcn32_1988* deletion
D*1988*-3F	TCCCGCTCTAGAATGATCACTTTCCATGACAACATC	*Sputcn32_1988* deletion
D*1988*-3R	CAGGACAAGCTTACACTTCATTCAAGTAGCGATGGT	*Sputcn32_1988* deletion
D*1988*-UF	GATGTGAAATCAGCGATGCGGTAC	*Sputcn32_1988* deletion
D*1988*-DR	TGGCTCAGCTTCACAGTATTTCGC	*Sputcn32_1988* deletion
D*1988*-InF	ATCTCATCTTGCTTTAGGTTAGAT	*Sputcn32_1988* deletion
D*1988*-InR	CGCTATGGATGCAATTAGTGGTCA	*Sputcn32_1988* deletion
D*1988*-OF	AGTTATCAGTCAGCGGTCGAGCAT	*Sputcn32_1988* deletion
D*1988*-OR	TTATCTACGCTTGGAGACATTACG	*Sputcn32_1988* deletion
D*2362*-5F	TTTTTCGAATTCAACATACTCTGAGCATATCAAAGG	*Sputcn32_2362* deletion
D*2362*-5R	GCGCTGGGATCCCAGGCTTGAATCTTATCTATTAAG	*Sputcn32_2362* deletion
D*2362*-3F	TGCTTTGGATCCTGCCACGCAATATGACTCCTTTAA	*Sputcn32_2362* deletion
D*2362*-3R	AACCCCCTGCAGTTAAAGGTCAGACTTGGGTATTAA	*Sputcn32_2362* deletion
D*2362*-UF	GTTCATCAATATGACGCTTACATC	*Sputcn32_2362* deletion
D*2362*-DR	GCTCCATTATTATCTTGCCGATTG	*Sputcn32_2362* deletion
D*2362*-InF	AACCTGAACCATCAATAAATTCAT	*Sputcn32_2362* deletion
D*2362*-InR	AGAGTGAACAACCTAGTACAGATA	*Sputcn32_2362* deletion
D*2362*-OF	GGACACTAGTGGTCTAACAATAAT	*Sputcn32_2362* deletion
D*2362*-OR	CACTTTGCCAGTTTAGATGCGCTG	*Sputcn32_2362* deletion
D*lrbR*-P_{aacC1}-*lrbR*5F	CGGAATTCCTATATCTAGAATAACTGCATCGG	P_{aacC1}-*lrbR* knock-in
D*lrbR*-P_{aacC}-*lrbR*5R	CATGCCATGGCGTTGCTGCTCCATAACATCAAAC	P_{aacC1}-*lrbR* knock-in
D*lrbR*-P_{aacC1}-*lrbR*3F	CATGCCATGGGGTGTTTATTGATTTCTTAAAGG	P_{aacC1}-*lrbR* knock-in
D*lrbR*-P_{aacC1}-*lrbR*3R	AACTGCAGATGGCCTGAAGATAGCGAACAAAT	P_{aacC1}-*lrbR* knock-in
D*lrbR*-P_{aacC1}-*lrbR*UF	GGTTTTCATGGCTTGTTATGACTG	P_{aacC1}-*lrbR* knock-in
D*lrbR*-P_{aacC1}-*lrbR*DR	AGTCAGATATATCCTGATTAGTGG	P_{aacC1}-*lrbR* knock-in
1235-Flag-ConF	ACCGCGGAATTCCGTGGTGGTGATTTTTGTGTAATC	1235 C-teminal 3×Flag knock-in
1235-Flag-ConR	GTTCCCCTGCAGACAATTTACTGGGATTAAGCTCAA	1235 C-teminal 3×Flag knock-in
1235-Flag-KinF	CGGGATCCTAGCCAGCATGTCGTAAGAGTGTGGACGGA	1235 C-teminal 3×Flag knock-in
1235-Flag-KinR	GCTCTAGACGACATGCTGGCAAAATGATTCCCCAAACT	1235 C-teminal 3×Flag knock-in

3×Flag (*1235*)-F	GC<u>TCTAGA</u>GGAGGTGGCGATTACAAGGATGAC	1235 C-teminal 3×Flag knock-in
1235-Flag-SF	CACCATTTTAACATTACTGCGAGT	1235 C-teminal 3×Flag knock-in
1235-Flag-SR	GCACAGAAGTGAGTAAACTCGACG	1235 C-teminal 3×Flag knock-in
1291-Flag-SF	ATTTGTCCTCTTACTACCCGATGT	1291 C-teminal 3×Flag knock-in
1291-Flag-SR	AGCAGTTACGATACAGTTGATACA	1291 C-teminal 3×Flag knock-in
3328-Flag-SF	CACTCGATTATTAGCCGCATTACT	3328 C-teminal 3×Flag knock-in
3328-Flag-SR	CTGGGTTTGATCGAGTTGATATTC	3328 C-teminal 3×Flag knock-in

[a] 5' phosphorylated primer. [b] 5'-FAM-labeled primer. Underline indicated restriction endonuclease sites.

Table S3. Reagent used in this study

REAGENT	SOURCE	IDENTIFIER
Antibodies		
Monoclonal ANTI-FLAG® M2 antibody produced in mouse	Sigma-Aldrich	Cat#F1804; RRID: AB_262044
Anti-Flag M2 antibody [DYKDDDDK-Tag (3B9) mAb]	Abmart	Cat#M20008S; RRID: AB_2713960
Mouse IgG	Abmart	Cat#B30010S; RRID: AB_3674223
Anti GST-tag mouse monoclonal antibody	CWBio Biosciences	Cat#CW0084; RRID: AB_2892808
Anti His-tag mouse monoclonal antibody	CWBio Biosciences	Cat#CW0286; RRID: AB_2736993
Anti-HA-tag antibody (HRP conjugated)	Abmart	Cat#M20021S; RRID: AB_2936257
HA-tag (26D11) mAb	Abmart	Cat#M20003S; RRID: AB_2864345
Anti HA-tag mouse monoclonal antibody	CWBio Biosciences	Cat#CW0092; RRID: AB_28929809
Goat anti-mouse IgG, HRP conjugated	CWBio Biosciences	Cat#CW0102; RRID: AB_2736997
Goat anti-mouse IgG HRP (light chain specific)	Abmart	Cat#M21004S; RRID: AB_3674224
Goat anti-mouse IgG-Fc secondary antibody (HRP)	SinoBiological	Cat#SSA006; RRID: AB_2892807
Chemicals, peptides, and recombinant proteins		
Yeast extract	Oxoid	Cat#LP0021
Tryptone	Oxoid	Cat#LP0042
Sodium chloride	Sinopharm Chemical Reagent Co., Ltd (SCRC)	Cat#10019318
Sodium lactate	TCI	Cat#S0928
HEPES	Solarbio Life Sciences	Cat#H8090
Potassium chloride	SCRC	Cat#10016318
Ammonium chloride	SCRC	Cat#10001518
Sodium dihydrogen phosphate dihydrate	SCRC	Cat#20040718
Disodium hydrogen phosphate dodecahydrate	SCRC	Cat#10020318
Sodium hydroxide	SCRC	Cat#10019718
Calcium chloride dihydrate	SCRC	Cat#20011160
Lithium chloride	SCRC	Cat#20022961
Sodium bicarbonate	SCRC	Cat#10018960
Ethylenediamine tetraacetic acid disodium salt dihydrate	SCRC	Cat#10009717
Manganese (II) chloride tetrahydrate	SCRC	Cat#20026118

Tween-20	SCRC	Cat#30189328
β-mercaptoethanol	SCRC	Cat#80076918
Crystal violet	SCRC	Cat#71012314
Bromophenol blue	SCRC	Cat#71008060
Formaldehyde solution	Macklin	Cat#F809702
Glycine	BioFroxx	Cat#1275
Deoxycholic acid, sodium salt	Amresco	Cat#0613-50G
Sodium dodecylsulfate	Solarbio Life Sciences	Cat#S8010
Bovine serum albumin	Solarbio Life Sciences	Cat#A8020
Salmon sperm DNA	Sigma-Aldrich	Cat#D1626
Kanamycin sulfate	Macklin	Cat#K6115
Ampicillin sodium	Macklin	Cat#A6265
TRIzon Reagent	CWBio Biosciences	Cat#CW0580S
Trichloromethane	SCRC	Cat#10006818
Isopropanol	SCRC	Cat#80109218
Ethanol	SCRC	Cat#10009218
Methanol	SCRC	Cat#10014118
M-MLV reverse transcriptase	Promega	Cat#M1701
Recombinant ribonuclease inhibitor	Takara	Cat#2313A
dNTP mix (10 mM each)	ThermoFisher Scientific	Cat#R0192
SYBR Green Master Mix	Biosharp Life Sciences	Cat#BL705A
isopropyl-β-D-thiogalactopyranoside (IPTG)	Solarbio Life Sciences	Cat#I8070
Ni Sepharose 6FF	Solarbio Life Sciences	Cat#P2010
Imidazole	SCRC	Cat#30104916
Glutathione-sepharose resin	Solarbio Life Sciences	Cat#P2020
L-Glutathione, reduced	Solarbio Life Sciences	Cat#G8180
Adenosine 3',5'-cyclic monophosphate sodium salt monohydrate (cAMP)	Sigma-Aldrich	Cat#A6885
Cyclic-di-GMP sodium salt	Sigma-Aldrich	Cat#SML1228
Lysozyme	ThermoFisher Scientific	Cat#90082
Lysozyme	Solarbio Life Sciences	Cat#L8120
DNase I, RNase-free	ThermoFisher Scientific	Cat#EN0521
Skim milk	Solarbio Life Sciences	Cat#D8340
Nonidet P-40 (NP-40)	Solarbio Life Sciences	Cat#N8030
Protease inhibitor cocktail	CWBio Biosciences	Cat#CW2200
PhosSTOP™ Phosphatase inhibitor cocktail tablet	Roche	Cat#04906845001
cOmplete™ EDTA-free protease inhibitor cocktail tablet	Roche	Cat#04693132001

Phos-tag™ Acrylamide	Wako	Cat# 300-93523
Recombinant Protein G-Sepharose™ 4B	Invitrogen	Cat#101242
Anti-FLAG Affinity Gel	Bimake	Cat#B23101
1 kb Ladder DNA marker	Biomed	Cat#MD114
BM2000+ DNA marker	Biomed	Cat#MD102
PageRuler™ pre-stained protein ladder	ThermoFisher Scientific	Cat#26616
HiMark™ pre-stained protein standard	ThermoFisher Scientific	Cat#LC5699
Multicolor Prestained Protein Ladder	Epizyme Biotech	Cat#WJ101
Wide-View™ Prestained Proein Size Marker III	Wako	Cat#230-02461
KOD-Plus-Neo DNA polymerase	Toyobo	Cat#KOD-401
2×M5 HiPer plus Taq HiFi PCR mix	Mei5Bio	Cat#MF002-plus-10
*Eco*RI-HF	New England Biolabs	Cat#R3101S
*Pst*I-HF	New England Biolabs	Cat#R3140S
*Bam*HI-HF	New England Biolabs	Cat#R3136S
*Hin*dIII-HF	New England Biolabs	Cat#R3104S
*Spe*I-HF	New England Biolabs	Cat#R3133S
*Xba*I	New England Biolabs	Cat#R0145S
*Xho*I	New England Biolabs	Cat#R0146S
T4 DNA ligase	ThermoFisher Scientific	Cat#EL0011
Proteinase K	Tiangen Biotech	Cat#RT403-01
Critical commercial assays		
TIANamp Bacteria DNA Kit	Tiangen Biotech	Cat#DP302-02
TIANprep Mini Plasmid Kit	Tiangen Biotech	Cat#DP103-02
Universal DNA Purification Kit	Tiangen Biotech	Cat#DP214-02
TURBO DNA-free Kit	ThermoFisher Scientific	Cat#AM1907
DIG Gel Shift Kit, 2nd generation	Roche	Cat#03353591910
Quick Start™ Bradford protein 1×dye reagent	Bio-Rad	Cat#5000205
B-PER Bacterial protein extraction reagent with enzymes	ThermoFisher Scientific	Cat#90078
Cyclic AMP ELISA Kit	Cayman Chemical	Cat#581001
Cyclic di-GMP ELISA Kit	Cayman Chemical	Cat#501780
Lactic Acid (LA) Content Assay Kit	Solarbio Life Sciences	Cat#BC2230
eECL Western Blot Kit	CWBio	Cat#CW0049
SuperFemto ECL Chemiluminescence Kit	Vazyme	Cat#E423-01
Other		
96-well tissue culture treated plates	NEST	Cat#701001
50-mL centrifuge tube	NEST	Cat#602052
Random primer (hexadeoxyribonucleotide mix: pd(N)6)	Takara	Cat#3801

Nylon membrane, positive charged	Roche	Cat#11417240001
PVDF western blotting membranes	Roche	Cat#03010040001
Medical X-ray film	Fuji	Cat#4741023951

Table S4. Oligonucleotides used in this study

Oligonucleotides	Sequence (5' to 3)	Usage
lrbA-Flag-SD-*lrbS*	CGGAATTCCATAAGAAACCCTACACACATGCTTTTAACTTGCTCCATTACAAACCAAAAG<br>ACCTCAACTATAATATGAATATGTGGCATAGAATAAATGATACAAATATATACATACGTT<br>ACTGCACGTAAAACTTACAACTTTAGATGTACACAGACACATAGTTCTACTATTTTATGA<br>TCATTCTAAAACTGAAATGGCTGAATAGCGTGTAGGGAAATTCCGAAATAAGAATGCTTT<br>TCACTTCAATAACATACAAACGGTTAGAATGTGTATCAAATTGTGGTAACCCACTATTAC<br>TTACCTAACCCTTGAATACTTCTTAACTCTGGAACGACATGAATGAAAAGAAAAATATTG<br>ATAGTGGATGATCACCCTGTGGTTGTTTTAGCACTCAAGATCATACTAGAACAAAATGGA<br>TTTGAGGTAATAGCAGACACTAATAATGGAGTTGATGCACTGAAACTCGTTAAAGATTTA<br>TCTCCCGATGCAGTTATTCTAGATATAGGTATTCCTCAACTTGATGGATTAGAAGTAATT<br>GAAAGGTCTAGAAAATTAGCAAATCCGCCACCAATATTAGTACTAACAGCACAACCCTCT<br>GACCATTTTGTCGTCCGCTGCATTCAAGCGGGCGCTTCTGGTTTTGTCTCCAAACAAAAA<br>GATATGACTGAGGTCACAGGCGCACTTCGAGCGATATTATCAGGCCATTCCTACTTTCCA<br>ATTTTTGGTAATAATATTATTACTCAATCGCACCAACAAGAAGCAGAACTGATAAAAAAG<br>CTTTCCACTCGTGAGATGGTCGTCCTACAACAACTTGCTATTGGATTATCAAATAAAGAA<br>ATTGCCGACCGTATGCTATTAAGTAATAAAACCATTAGTACTTATAAGACGAGACTCCTT<br>GAAAAGCTTAATGCTAAGACATTAGTAGATTTAATTGAAATAGCGAAGCGAAACAGCATC<br>ATAGGAGGTGGCGATTACAAGGATGACGACGATAAGGACTATAAGGACGATGATGACAAG<br>GACTACAAAGATGATGACGATAAATGATAATGCTAAGACATTAGTAGATTTAATTGAAAT<br>AGCGAAGC**GAAACAG**CATCATATGAAGATCATATTGTCATTAATTTTTTCTTTATTCTTT<br>ATGCTATTGGCTTCAATACCACTCAAAGCCAAAGAAGATGTACCGCTTACGCTATATGGT<br>CACTCTACTGTAGGAGAAGTAAAGCTAGATCTCTCAAAAGAGCAACAAGATTGGCTTCAA<br>CAACATGGTAAGATCCGTGTTGGTATCACAACACCTGATTACCCGCCCTTTGATATGACA<br>ATGGATGGCCATAGCAAATACTATGAAGGGCTAAGTGCCGATTATCTACAAATACTCTCA<br>GAAATATTAAAGGTGAAAATAGAGTTACACTTTTTTGATTCGCGCCCTAAAGCAATAGAT<br>GCCATCAAAAATAATGATGTCGATATGCTGACCACGGCCAATCGTTATGAAGAGTTTTAT<br>GGTCTAGAACTGAGCCAGCATTATGTTCTGCAGCC	knock-in<br>*lrbA*-Flag

Underline indicates *flag* sequences. Boxes indicate start and stop codons. Bold indicates predicted SD sequences.

Supporting information references

1. C. Liu *et al.*, cAMP and c-di-GMP synergistically support biofilm maintenance through the direct interaction of their effectors. *Nat. Commun.* **13**, 1493 (2022).
2. A. Schäfer *et al.*, Small mobilizable multi purpose cloning vectors derived from the *Escherichia coli* plasmids pK18 and pK19: selection of defined deletions in the chromosome of *Corynebacterium glutamicum*. *Gene* **145**, 69-73 (1994).
3. M. E. Kovach *et al.*, Four new derivatives of the broad-host-range cloning vector pBBR1MCS, carrying different antibiotic-resistance cassettes. *Gene* **166**, 175-176 (1995).
4. C. Liu *et al.*, Sodium lactate negatively regulates *Shewanella putrefaciens* CN32 biofilm formation via a three-component regulatory system (LrbS-LrbA-LrbR). *Appl. Environ. Microbiol,* **83**, e00712-17 (2017).
5. W. Liu *et al.*, ROK family regulator NagC promotes prodigiosin biosynthesis independent of *N*-acetylglucosamine in *Serratia* sp. ATCC 39006. *Appl. Environ. Microbiol.* **90**, e00891-24 (2024).
6. A. Kaczmarczyk *et al.*, Precise timing of transcription by c-di-GMP coordinates cell cycle and morphogenesis in *Caulobacter*. *Nat. Commun.* **11**, 816 (2020).
7. C. Wu *et al.*, Oxygen promotes biofilm formation of *Shewanella putrefaciens* CN32 through a diguanylate cyclase and an adhesin. *Sci. Rep.* **3**, 1945 (2013).
8. F. M. Rossmann *et al.*, The GGDEF domain of the phosphodiesterase PdeB in *Shewanella putrefaciens* mediates recruitment by the polar landmark protein HubP. *J. Bacteriol.* **201**, e0053418 (2019).
